# Selective and Reversible 1,3-Dipolar Cycloaddition of 2-(2-Oxoindoline-3-ylidene)acetates with Nitrones in the Synthesis of Functionalized Spiroisoxazolidines

**DOI:** 10.3390/ijms232012639

**Published:** 2022-10-20

**Authors:** Dmitriy D. Karcev, Mariia M. Efremova, Alexander P. Molchanov, Nikolai V. Rostovskii, Mariya A. Kryukova, Alexander S. Bunev, Dmitry A. Khochenkov

**Affiliations:** 1 Institute of Chemistry, Saint Petersburg State University, Universitetsky pr. 26, 198504 Saint Petersburg, Russia; 2 Medicinal Chemistry Center, Togliatti State University, Belorusskaya st. 14, 445020 Togliatti, Russia; 3 Blokhin National Medical Center of Oncology, Kashirskoe Shosse 24, 115478 Moscow, Russia

**Keywords:** 1,3-dipolar cycloaddition, spiroisoxazolidines, nitrones, regioselectivity, diastereoselectivity

## Abstract

The 1,3-dipolar cycloaddition of 2-(2-oxoindoline-3-ylidene)acetates with functionalized aldo- and ketonitrones proceeds with good selectivity to provide new highly functionalized 5-spiroisoxazolidines. A characteristic feature of these reactions is reversibility that allows for the control of the diastereoselectivity of cycloaddition. The reduction of obtained adducts using zinc powder in acetic acid leads to 1,3-aminoalcohols or spirolactones. For a number of the spiro compounds obtained, anticancer activity was found.

## 1. Introduction

In recent years, spiroheterocyclic scaffolds have attracted increased interest in the search for promising candidates for the development of new medicines [1,2,3,4]. Moreover, spirocyclic motifs can often be found in natural compounds [5]. It is known that spiro derivatives of indolin-2-one are privileged structures in medicinal chemistry [6,7,8], due to their wide range of biological properties, including anti-inflammatory [9], anticancer [10,11], and antimycobacterial activity [12]. In addition, this structural fragment is common to indolinone alkaloids [13].

**Scheme 1:**
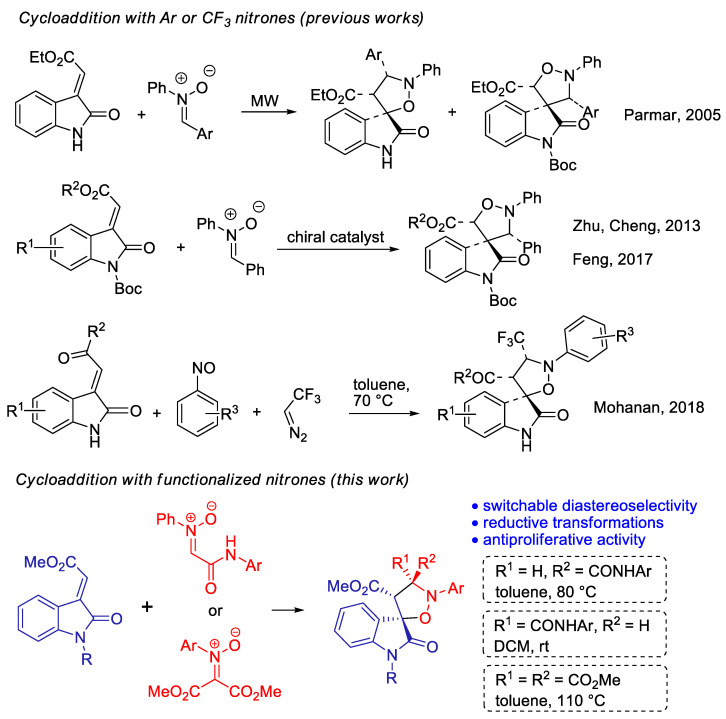
The 1,3-dipolar cycloaddition of 2-(2-oxoindoline-3-ylidene)acetates with nitrones [31,33,35,36].

In the last decade, the number of methodologies for the synthesis of spiroindolin-2-one derivatives has been growing rapidly [14,15]. One of the most effective among them is based on 1,3-dipolar cycloaddition reactions [7,16,17]. The most investigated cycloadditions using indolin-2-one-based dipolarophiles are reactions with azomethine ylides, which make it possible to obtain spiropyrazolines in high yields and selectivity [18,19,20,21,22,23,24]. Promising biological properties were noted for the obtained cycloadducts [25,26,27,28,29,30]. However, the cycloaddition reactions of nitrones to indolin-2-one-based dipolarophiles are represented by only a few examples [31,32,33,34,35,36,37] (Scheme 1). It was found that such reactions can proceed with different regio- and stereoselectivity, depending not only on the structure of the starting compounds but also on the reaction conditions. The prediction of selectivity in the case of 2-(2-oxoindoline-3-ylidene)acetates is complicated due to the reversibility of the nitrone cycloaddition reactions [34,37]. Previously, the reactions with aldonitrones that did not contain additional functional groups were most studied. However, it is known that the transition from aldo- to ketonitrones or the introduction of additional functional groups in the α-position of the nitrone can dramatically affect the regio- and stereoselectivity of the 1,3-dipolar cycloaddition reactions [38,39,40].

Isoxazolidine derivatives, the products of the cycloaddition of nitrones to the C=C bond, are of considerable interest due to the wide range of their biological properties [41,42,43,44,45,46,47,48,49,50,51]. Moreover, due to the ease of the N–O bond cleavage in the isoxazolidine ring under reductive conditions, these compounds can be used for the synthesis of 1,3-aminoalcohols [52,53,54], biologically valuable β-lactams [55,56,57,58,59], and amino acids [60]. The presence of additional functional groups in the isoxazolidine ring significantly expands the possibilities of using these substrates in organic synthesis [61,62].

In this work, the regio- and stereoselectivity of the 1,3-dipolar cycloaddition reactions of functionalized aldo- and ketonitrones with 2-(2-oxoindoline-3-ylidene)acetates were investigated for the first time. As a result, a series of novel 5-spiroisoxazolidines were synthesized with controllable diastereoselectivity. The introduction of the ester group at the C3 of isoxazolidines makes it possible to redirect the reduction from 1,3-aminoalcohols to spirolactones. In addition, the anticancer activity of the obtained functionalized spiroisoxazolidine derivatives was evaluated.

## 2. Results and Discussion

### 2.1. Synthesis and Structural Characterization

First, we studied the reactions of 2-(2-oxoindoline-3-ylidene)acetates **1** with *C*-carbamoyl aldonitrones **2**. A short optimization of the reaction conditions was carried out for the reaction of **1a** with nitrone **2a** (Table 1). We obtained the mixtures of isomeric cycloadducts with a predominance of 5-spiroisoxazolidines **3** in all variants of the tested reaction conditions. The stereoselectivity of the reactions was found to be dependent on the conditions. Heating in toluene at 80 °C was optimal for obtaining diastereomer **3a** as the main product (Table 1, Entry 2). At a higher temperature, **3a** remained the main reaction product, but its yield decreased due to the considerable tarring of the reaction mixture (Table 1, Entry 3). We obtained diastereomer **3′a** as the main product of the reactions conducted at room temperature in dichloromethane (DCM). In this case, we had to change the solvent from toluene to DCM due to the low solubility of compounds **1a** and **2a** in toluene (Table 1, Entry 4).

It was found that the reactions of 2-(2-oxoindoline-3-ylidene)acetates **1a,b** with *C*-carbamoyl nitrones **2a–d** proceed regio- and stereoselectively in toluene at 80 °C, predominantly obtaining 5-spiroisoxazolidines **3** (Table 2). In most experiments, the minor products **3′** and **4** could not be separated chromatographically, and in the table their yields are presented as summary.

At the same time, it was possible to selectively obtain diastereomers **3′** when carrying out the reaction in DCM at room temperature (Table 3). In this case, we observed no signals of regioisomeric products **4** in the ^1^H NMR spectra of the crude reaction mixtures. The compound **3′e** was also obtained as a major product (yield 54%) in toluene at 55 °C for 7 h. 

**Figure 1:**
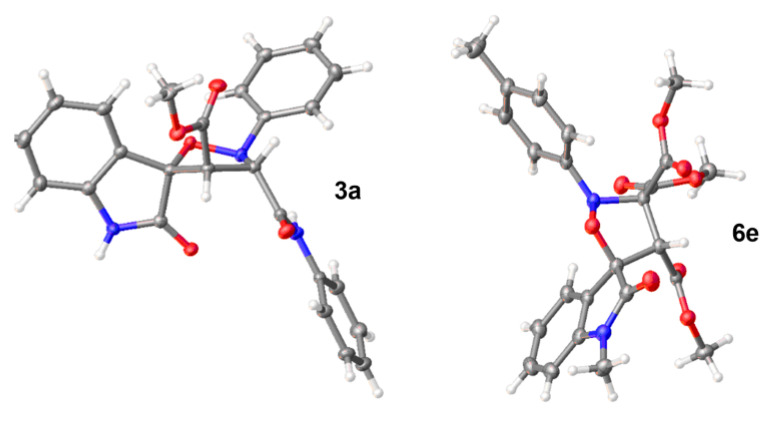
X-ray structures of products **3a** and **6e**.

To confirm the stereochemistry of adducts **3** and **4**, X-ray analysis data for compound **3a** (Figure 1) and ^1^H-^1^H NOESY NMR spectra for compounds **3h**, **3′h** (Figures S1 and S2 in SI) and **4c** (Figure S6 in SI) were used.

**Figure 2:**
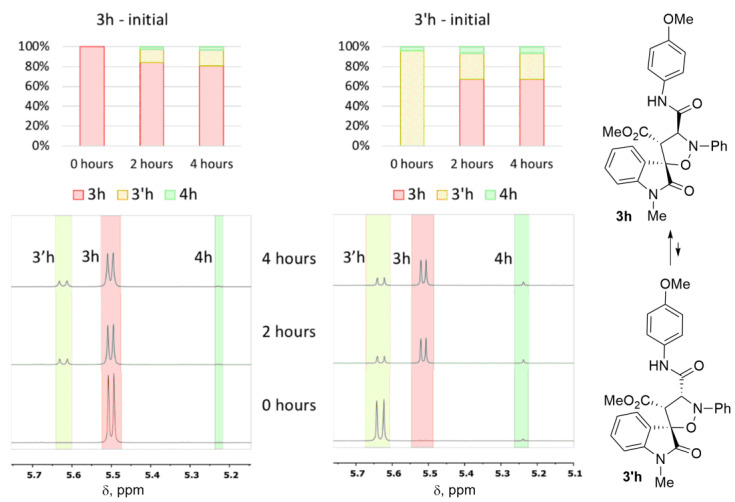
^1^H NMR monitoring of thermal interconversion between isoxazolidines **3h** and **3′h**.

Reversibility was observed for the 1,3-dipolar cycloaddition reactions of nitrones [50,63]. In our case, the ratio of isomeric compounds **3** and **3′** depends on the reaction temperature. Furthermore, the isolated compounds **3′** were noticed to undergo transformation when kept in a CDCl_3_ solution overnight. The signals of reagents **1** and **2** along with the signals of stereoisomers **3** appeared in the ^1^H NMR spectrum of the sample. In this regard, we further inspected the reversibility of the cycloaddition reactions of *C*-carbamoyl nitrones **2** under the reaction conditions. ^1^H NMR spectra were recorded before and after heating individual compounds **3h** and **3′h** at 80 °C for 4 h (two times for 2 h) in C_6_D_6_. After heating, the spectra of both samples contained the signals of all three isomers **3h**, **3′h**, and **4h**, as well as the signals of reagents **1b** and **2d**, and the ratio of the compounds **3h**:**3′h**:**4h**:**2d**:**1b** was 5:1:0.05:1:1 for **3h** and 3:1:0.3:1.6:1.6 for **3′h** (Figure 2). In both experiments, the product **3h** was the main component of the mixture and the ratio of compounds **2d**:**1b** was 1:1. The obtained results provide evidence for the studied reaction to proceed reversibly under the applied conditions.

Next, we investigated the reactions of dipolarophiles **1a,b** with ketonitrones **5a–c** containing two ester groups (Table 4). As noted in the introduction, the regioselectivity of their cycloadditions can often be different compared to aldonitrones. The reaction with more sterically hindered *C,C*-bis(methoxycarbonyl)nitrones **5** required more harsh conditions, and the cycloaddition was carried out at 110 °C. The reactions proceeded to obtain only 5-spiro regioisomers **6a–f** in good-to-high yields. The signals of regioisomeric products were not observed in the ^1^H NMR spectra of crude reaction mixtures. The regioselectivity of the reaction in this case can be explained by steric factors. The structure of the cycloadducts **6** was further confirmed using X-ray analysis data for compound **6e** (Figure 1). 

Compounds **6** showed greater stability than compounds **3** and **3′**: the ^1^H NMR spectrum of **6** did not change when the sample was kept in a CDCl_3_ solution for two days at room temperature. However, the noticeable reversibility was indicated under the used cycloaddition reaction conditions. When pure compound **6b** was heated in toluene at 110 °C for 2 h, the ^1^H NMR spectrum and TLC showed the presence of the starting compounds **1a** and **5b** in the solution along with the signals of compound **6b**. Notably, isomerization or cycloreversion was not observed for solutions of compounds **3h** and **6b** in DMSO-d_6_ at room temperature within two days.

### 2.2. Transformations of the Cycloadducts

**Scheme 2:**
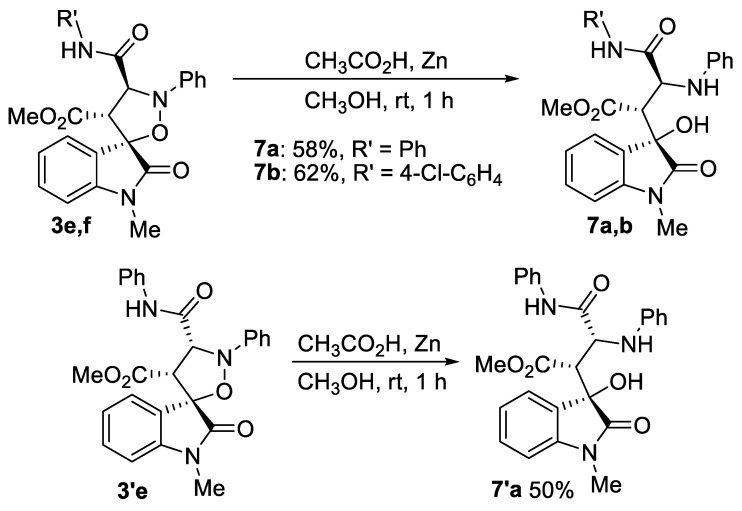
Synthesis of 1,3-aminoalcohols **7** from **3** and **3′**.

Next, we studied the possibility of selective opening of the isoxazolidine ring under the action of zinc in acetic acid. The selective conversion to the corresponding amino alcohols was shown for both stereoisomeric adducts with aldonitrones **3** and **3′**, at room temperature for 1 h (Scheme 2). Notably, the reduction of diastereomeric isoxazolidines **3e** and **3′e** made it possible to obtain diastereomeric amino alcohols **7a** and **7′a,** correspondingly.

The relative configuration of the stereocenters in **7a** and **7′a** was confirmed by ^1^H-^1^H NOESY spectra (see Figures S3 and S4 in SI) and the comparison of these data with those of isoxazolidines **3e** and **3′e**. 

**Scheme 3:**
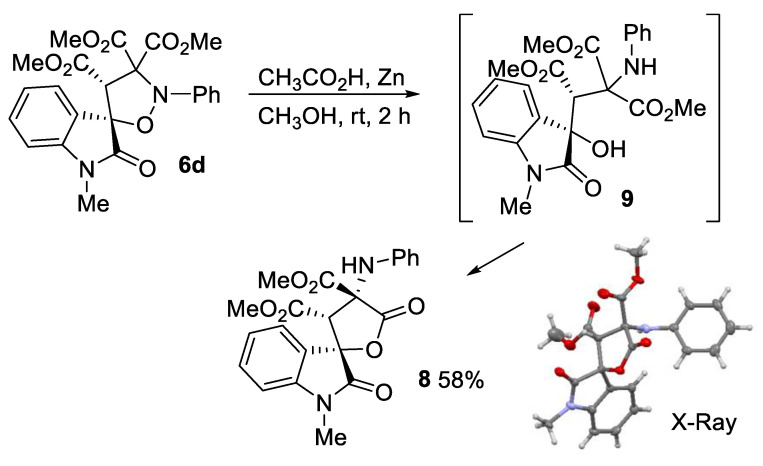
Synthesis of spirolactone **8** from **6d**.

A similar reaction of cycloadduct **6d** gave spirolactone **8** (Scheme 3). Presumably, in this case, the amino alcohol **9** is formed at the first stage, which is converted to lactone **8** in an acidic medium. The structure of compound **8** was confirmed by ^1^H-^1^H NOESY spectra (see Figure S5 in SI) and X-ray analysis data (Scheme 3). 

### 2.3. Antiproliferative Activity

**Figure 3:**
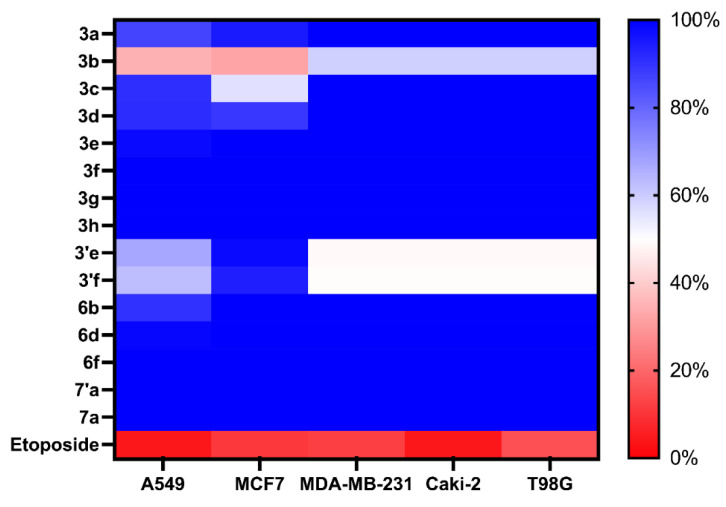
Cytotoxic activity (cell viability, %) of selected compounds against several cancer cell lines at 50 μM concentration.

Given the high potential of spiro derivatives of indolin-2-one in medicinal chemistry, it was of keen interest to test the bioactivity potential of the novel synthesized spiro compounds **3**, **3′**, and **6**, as well as amino alcohols **7**. They were evaluated for antiproliferative activity on several tumor cell lines: A549 (lung carcinoma), MCF7 (breast cancer), MDA-MB-231 (triple-negative breast cancer), Caki-2 (kidney clear cell carcinoma), and T98G (glioblastoma multiforme) (Figure 3). The highest cytotoxicity was observed for compound **3b**, against the MCF7 (breast cancer) and A549 (lung carcinoma) cell lines. At a concentration of 50 μM, a 32% inhibition for MCF7 was achieved relative to the control (etoposide). Compounds **3a,d–h**, **6**, and **7** showed no inhibitory activity on all tested cell lines.

## 3. Materials and Methods

### 3.1. General Information

All the cycloaddition reactions were performed in anhydrous solvents under an argon atmosphere. Toluene was distilled over sodium. Reaction progress was monitored using thin layer chromatography (TLC) on precoated Silufol UV–254 plates. ^1^H and ^13^C NMR spectra were recorded in CDCl_3_, benzene-*d*_6_, DMSO-*d*_6_ using a Bruker Avance 400 spectrometer (see ^1^H and ^13^C NMR spectra in SI). HRMS spectra were obtained with a Bruker-maXis (QTOF). Xcalibur, Eos diffractometer was used for X-ray analysis. (*E*)-methyl 2-(2-oxoindolin-3-ylidene)acetates **1a,b** and nitrones **2a–d**, **5a–c** were prepared using known procedures [61,64,65]. 

### 3.2. Synthetic Methods and Analytic Data of Compounds

#### 3.2.1. General Procedure for Obtaining Cycloadducts **3**, **3′**, and **4**

A mixture of nitrone **2a–d** (1.5 eqv.) and dipolarophile **1a,b** (1 eqv.) was stirred in toluene (10 mL) at 80 °C for 14 h. The solvent was removed under reduced pressure. Products were separated by column chromatography (silica gel, hexane:ethyl acetate 3:1).

*rac-(3R,3′R,4′S)-methyl 2-oxo-2′-phenyl-3′-(phenylcarbamoyl)spiro[indoline-3,5′-isoxazolidine]-4′-carboxylate* (**3a**), *rac-(3R,3′S,4′S)-methyl 2-oxo-2′-phenyl-3′-(phenylcarbamoyl)spiro[indoline-3,5′-isoxazolidine]-4′-carboxylate* (**3′a**) and *rac-(3R,3′S,5′R)-methyl 2-oxo-2′-phenyl-3′-(phenylcarbamoyl)spiro[indoline-3,4′-isoxazolidine]-5′-carboxylate* (**4a**) were obtained from (2-oxoindoline-3-ylidene)acetate **1a** (102 mg, 0.5 mmol) and nitrone **2a** (180 mg, 0.75 mmol). 

Isomer **3a**. Yield 155 mg (70%). Colorless solid, m.p. 178–180 °C. ^1^H NMR (400 MHz, CDCl_3_): δ 9.41 (s, 1H, NH), 8.85 (s, 1H, NH), 7.67 (d, *J* = 7.7 Hz, 2H_Ar_), 7.39–7.27 (m, 4H_Ar_), 7.26–7.19 (m, 1H_Ar_), 7.19–7.04 (m, 4H_Ar_), 6.94–6.75 (m, 3H_Ar_), 5.12 (d, *J* = 5.8 Hz, 1H, CH), 4.28 (d, *J* = 5.8 Hz, 1H, CH), 3.37 (s, 3H, CO_2_CH_3_). ^13^C NMR (101 MHz, CDCl_3_): δ 175.4 (C), 168.5 (C), 167.6 (C), 148.7 (C), 141.4 (C), 137.6 (C), 131.4 (CH), 129.2 (2CH), 129.2 (2CH), 126.5 (CH), 124.8 (CH), 123.7 (CH), 123.4 (C), 123.2 (CH), 120.1 (2CH), 115.8 (2CH), 111.0 (CH), 84.7 (C-O), 70.9 (CH), 59.8 (CH), 52.7 (CH_3_). HRMS (ESI): (M + Na)^+^, found 466.1379. [C_25_H_21_N_3_O_5_Na]^+^ calculated 466.1373. Appropriate crystals for X-ray analysis were obtained from hexane/ethyl acetate solution. Crystallographic data for **3a** have been deposited with the Cambridge Crystallographic Data Centre, no. CCDC 2177789.

Isomers **3′a** and **4a**. Yield 40 mg (18%). Ratio: 4:1. Yellowish solid. ^1^H NMR (400 MHz, CDCl_3_): **3′a**: δ 9.00 (s, 1H, NH), 8.20 (s, 1H, NH), 5.34 (d, *J* = 7.9 Hz, 1H, CH), 4.25 (d, *J* = 7.9 Hz, 1H, CH), 3.64 (s, 3H, CH_3_), the aromatic signals of the isomers overlap. **4a**: δ 9.12 (s, 1H, NH), 8.54 (s, 1H, NH), 5.12 (s, 1H, CH), 4.95 (s, 1H, CH), 3.41 (s, 3H, CH_3_), The aromatic signals of the isomers overlap. ^13^C NMR (101 MHz, DMSO-*d_6_*): **3′a**: δ 174.2 (C), 167.5 (C), 166.7 (C), 150.0 (C), 143.0 (C), 138.3 (C), 131.0 (CH), 128.6 (2CH), 128.6 (2CH), 126.5 (CH), 123.9 (CH), 123.4 (C), 122.1 (CH), 121.9 (CH), 120.3 (2CH), 114.6 (2CH), 110.2 (CH), 83.5 (C-O), 68.9 (CH), 56.6 (CH), 51.8 (CO_2_CH_3_). HRMS (ESI): (M + H)^+^, found 444.1559. [C_25_H_21_N_3_O_5_H]^+^ calculated 444.1554.

*rac-(3R,3′R,4′S)-methyl 3′-((4-chlorophenyl)carbamoyl)-2-oxo-2′-phenylspiro[indoline-3,5′-isoxazolidine]-4′-carboxylate* (**3b**)**,** *rac-(3R,3′S,4′S)-methyl 3′-((4-chlorophenyl)carbamoyl)-2-oxo-2′-phenylspiro[indoline-3,5′-isoxazolidine]-4′-carboxylate* (**3′b**) and *rac-(3R,3′S,5′R)-methyl 3′-((4-chlorophenyl)carbamoyl)-2-oxo-2′-phenylspiro[indoline-3,4′-isoxazolidine]-5′-carboxylate* (**4b**) were obtained from (2-oxoindoline-3-ylidene)acetate **1a** (102 mg, 0.5 mmol) and nitrone **2b** (206 mg, 0.75 mmol). 

Isomer **3b**. Yield 167 mg (70%). Yellowish solid, m.p. 184–186 °C. ^1^H NMR (400 MHz, DMSO-*d_6_*): δ 10.95 (s, 1H, NH), 10.60 (s, 1H, NH), 7.76–7.69 (m, 2H_Ar_), 7.45–7.36 (m, 2H_Ar_), 7.33–7.22 (m, 3H_Ar_), 7.10–6.97 (m, 3H_Ar_), 6.91 (d, *J* = 7.7 Hz, 1H_Ar_), 6.86–6.77 (m, 1H_Ar_), 6.62 (d, *J* = 7.7 Hz, 1H_Ar_), 5.17 (d, *J* = 7.5 Hz, 1H, CH), 4.40 (d, *J* = 7.5 Hz, 1H, CH), 3.25 (s, 3H, CO_2_CH_3_). ^13^C NMR (101 MHz, DMSO-*d_6_*): δ 172.4 (C), 167.5 (C), 167.0 (C), 149.2 (C), 142.3 (C), 137.2 (C), 130.9 (CH), 128.7 (2CH), 128.6 (2CH), 127.8 (C), 125.6 (CH), 124.7 (C), 122.7 (CH), 121.7 (CH), 121.7 (2CH), 115.2 (2CH), 110.5 (CH), 83.9 (C-O), 69.0 (CH), 59.4 (CH), 52.3 (CO_2_CH_3_). HRMS (ESI): (M + Na)^+^, found 500.0990. [C_25_H_20_ClN_3_O_5_Na]^+^ calculated 500.0984.

Isomers **3′b** and **4b**. Yield: 38 mg (16%). Ratio: 1.7:1. Yellow oil. ^1^H NMR (400 MHz, CDCl_3_): **3′b**: δ 9.00 (s, 1H, NH), 7.86 (s, 1H, NH) 5.30 (d, *J* = 7.9 Hz, 1H, CH), 4.22 (d, *J* = 7.9 Hz, 1H, CH), 3.61 (s, 3H, CO_2_CH_3_), the aromatic signals of the isomers overlap. **4b**: δ 9.09 (s, 1H, NH), 8.18 (s, 1H, NH) 5.08 (s, 1H, CH), 4.90 (s, 1H, CH), 3.39 (s, 3H, CO_2_CH_3_), the aromatic signals of the isomers overlap. ^13^C NMR (101 MHz, DMSO-*d_6_*): **3′b**: δ 174.2 (C), 167.5 (C), 167.0 (C), 150.0 (C), 143.0 (C), 137.3 (C), 131.1 (CH), 128.6 (2CH), 128.6 (2CH), 127.7 (C), 126.5 (CH), 123.4 (C), 122.2 (CH), 122.0 (2CH), 121.9 (CH), 114.6 (2CH), 110.3 (CH), 83.5 (C-O), 69.0 (CH), 56.6 (CH), 51.8 (CO_2_CH_3_). HRMS (ESI): (M + Na)^+^, found 500.0990. [C_25_H_20_ClN_3_O_5_Na]^+^ calculated 500.0984.

*rac-(3R,3′R,4′S)-methyl 2-oxo-2′-phenyl-3′-(p-tolylcarbamoyl)spiro[indoline-3,5′-isoxazolidine]-4′-carboxylate (***3c**), *rac-(3R,3′S,4′S)-methyl 2-oxo-2′-phenyl-3′-(p-tolylcarbamoyl)spiro[indoline-3,5′-isoxazolidine]-4′-carboxylate (***3′c**) and *rac-(3R,3′S,5′R)-methyl 2-oxo-2′-phenyl-3′-(p-tolylcarbamoyl)spiro[indoline-3,4′-isoxazolidine]-5′-carboxylate* (**4c**) were obtained from (2-oxoindoline-3-ylidene)acetate **1a** (28 mg, 0.133 mmol) and nitrone **2c** (52 mg, 0.204 mmol). 

Isomer **3c**. Yield 35 mg (57%). Yellowish solid, m.p. 178–181 °C. ^1^H NMR (400 MHz, CDCl_3_): δ 9.33 (s, 1H, NHCO), 9.02 (s, 1H, NHCO), 7.55 (d, *J* = 7.9 Hz, 2H_Ar_), 7.36–7.27 (m, 2H_Ar_), 7.25–7.18 (m, 1H_Ar_), 7.17–7.04 (m, 5H_Ar_), 6.92–6.80 (m, 2H_Ar_), 6.77 (d, *J* = 7.9 Hz, 1H_Ar_), 5.12 (d, *J* = 5.8 Hz, 1H, CH), 4.29 (d, *J* = 5.8 Hz, 1H, CH), 3.36 (s, 3H, CO_2_CH_3_), 2.33 (s, 3H, H_3_C-C_6_H_4_). ^13^C NMR (101 MHz, CDCl_3_): δ 175.4 (C), 168.6 (C), 167.4 (C), 148.8 (C), 141.4 (C), 135.0 (C), 134.4 (C), 131.3 (CH), 129.6 (2CH), 129.2 (2CH), 126.5 (CH), 123.6 (CH), 123.4 (C), 123.1 (CH), 120.1 (2CH), 115.7 (2CH), 111.0 (CH), 84.8 (C-O), 70.8 (CH), 59.8 (CH), 52.7 (CO_2_CH_3_), 21.1 (H_3_C-C_6_H_4_). HRMS (ESI): (M + Na)^+^, found 480.1536. [C_26_H_23_N_3_O_5_Na]^+^ calculated 480.1530.

Isomers **3′c** and **4c**. Yield: 5 mg (8%). Ratio: 1.2:1. Beige solid. ^1^H NMR (400 MHz, CDCl_3_): **3′c**: δ 8.92 (s, 1H, NHCO), 7.56 (s, 1H, NHCO), 5.49 (d, *J* = 7.9 Hz, 1H, CH), 4.42 (d, *J* = 7.9 Hz, 1H, CH), 3.62 (s, 3H, CO_2_CH_3_), 2.33 (s, 3H, H_3_C-C_6_H_4_), the aromatic signals of the isomers overlap. **4c**: δ 9.00 (s, 1H, NHCO), 7.97 (s, 1H, NHCO), 5.09 (s, 1H, CH), 4.90 (s, 1H, CH), 3.39 (s, 3H, CO_2_CH_3_), 2.27 (s, 3H, H_3_C-C_6_H_4_), the aromatic signals of the isomers overlap. ^13^C NMR (101 MHz, CDCl_3_): **3′c**: δ 173.3 (C), 167.6 (C), 165.4 (C), 150.2 (C), 141.1 (C), 134.7 (C), 134.7 (C), 131.6 (CH), 129.8 (2CH), 129.6 (2CH), 124.9 (C), 124.7 (CH), 124.0 (CH), 122.9 (CH), 120.4 (2CH), 114.6 (2CH), 110.4 (CH), 83.8 (C-O), 69.6 (CH), 59.9 (CH), 52.5 (CO_2_CH_3_), 21.1 (H_3_C-C_6_H_4_). **4c**: δ 174.0 (C), 168.1 (C), 164.6 (C), 149.6 (C), 141.9 (C), 135.0 (C), 133.7 (C), 130.1 (CH), 129.7 (2CH), 128.9 (2CH), 125.1 (CH), 124.6 (C), 123.7 (CH), 123.0 (CH), 120.1 (2CH), 114.5 (2CH), 110.6 (CH), 85.8 (C-O), 78.5 (CH), 64.8 (CH), 53.2 (CO_2_CH_3_), 21.0 (H_3_C-C_6_H_4_). HRMS (ESI): (M + Na)^+^, found 480.1536. [C_26_H_23_N_3_O_5_Na]^+^ calculated 480.1530.

Isomer **3′c**. Yield 10 mg (16%). Pale yellow solid, m.p. 163–166 °C (recrystallized from Et_2_O). ^1^H NMR (400 MHz, DMSO-*d_6_*): ^1^H NMR (400 MHz, DMSO-*d*_6_) δ 10.71 (s, 1H, NHCO), 10.04 (s, 1H_,_ NHCO), 7.61–7.48 (m, 3H_Ar_), 7.38–7.22 (m, 3H_Ar_), 7.17–7.09 (m, 2H_Ar_), 7.04–6.85 (m, 5H_Ar_), 5.06 (d, *J* = 8.4 Hz, 1H, CH), 4.30 (d, *J* = 8.4 Hz, 1H, CH), 3.39 (s, 3H, CO_2_CH_3_), 2.27 (s, 3H, H_3_C-C_6_H_4_). ^13^C NMR (101 MHz, DMSO-*d_6_*) δ 174.2 (C), 167.5 (C), 166.4 (C), 150.0 (C), 143.0 (C), 135.8 (C), 133.0 (C), 131.0 (CH), 129.0 (2CH), 128.6 (2CH), 126.5 (CH), 123.5 (C), 122.1 (CH), 121.9 (CH), 120.4 (2CH), 114.6 (2CH), 110.2 (CH), 83.5 (C-O), 69.0 (CH), 56.5 (CH), 51.8 (CO_2_CH_3_), 20.5 (H_3_C-C_6_H_4_). HRMS (ESI): (M + Na)^+^, found 480.1537. [C_26_H_23_N_3_O_5_Na]^+^ calculated 480.1530.

*rac-(3R,3′R,4′S)-methyl 3′-((4-methoxyphenyl)carbamoyl)-2-oxo-2′-phenylspiro[indoline-3,5′-isoxazolidine]-4′-carboxylate* (**3d**), *rac-(3R,3′S,4′S)-methyl 3′-((4-methoxyphenyl)carbamoyl)-2-oxo-2′-phenylspiro[indoline-3,5′-isoxazolidine]-4′-carboxylate* (**3′d**) and *rac-(3R,3′S,5′R)-methyl 3′-((4-methoxyphenyl)carbamoyl)-2-oxo-2′-phenylspiro[indoline-3,4′-isoxazolidine]-5′-carboxylate* (**4d**) were obtained from (2-oxoindoline-3-ylidene)acetate **1a** (102 mg, 0.5 mmol) and nitrone **2d** (203 mg, 0.75 mmol). 

Isomer **3d.** Yield 164 mg (69%). Beige solid, m.p. 167–170 °C. ^1^H NMR (400 MHz, CDCl_3_): δ 9.28 (s, 1H, NH), 8.83 (s, 1H, NH), 7.56 (d, *J* = 7.9 Hz, 2H_Ar_), 7.35–7.27 (m, 2H_Ar_), 7.25–7.19 (m, 1H_Ar_), 7.16–7.03 (m, 3H_Ar_), 6.92–6.84 (m, 3H_Ar_), 6.84–6.76 (m, 2H_Ar_), 5.12 (d, *J* = 5.5 Hz, 1H, CH), 4.28 (d, *J* = 5.5 Hz, 1H, CH), 3.79 (s, 3H, OCH_3_), 3.36 (s, 3H, CO_2_CH_3_). ^13^C NMR (101 MHz, CDCl_3_): δ 175.3 (C), 168.5 (C), 167.3 (C), 156.8 (C), 148.8 (C), 141.4 (C), 131.3 (CH), 130.8 (C), 129.2 (2CH), 126.5 (CH), 123.6 (CH), 123.5 (C), 123.1 (CH), 121.7 (2CH), 115.7 (2CH), 114.3 (2CH), 111.0 (CH), 84.8 (C-O), 70.7 (CH), 59.9 (CH), 55.6 (OCH_3_), 52.8 (CO_2_CH_3_). HRMS (ESI): (M + H)^+^, found 474.1665. [C_26_H_23_N_3_O_6_H]^+^ calculated 474.1660.

Isomers **3′d** and **4d**. Yield: 45 mg (19%). Ratio: 1.7:1. Beige solid. ^1^H NMR (400 MHz, CDCl_3_): **3′d**: δ 8.87 (s, 1H, NH), 8.04 (s, 1H, NH), 5.29 (d, *J* = 7.9 Hz, 1H, CH), 4.21 (d, *J* = 7.9 Hz, 1H, CH), 3.80 (s, 3H, OCH_3_), 3.61 (s, 3H, CO_2_CH_3_), the aromatic signals of the isomers overlap. **4d**: δ 8.96 (s, 1H, NH), 5.08 (s, 1H, CH), 4.89 (s, 1H, CH), 3.74 (s, 3H, OCH_3_), 3.39 (s, 3H, CO_2_CH_3_), the aromatic signals of the isomers overlap. ^13^C NMR (101 MHz, CDCl_3_): **3′d**: δ 174.6 (C), 168.6 (C), 166.9 (C), 157.0 (C), 151.1 (C), 141.8 (C), 131.9 (CH), 130.3 (C), 129.1 (2CH), 126.2 (CH), 123.5 (CH), 122.8 (CH), 122.2 (2CH), 121.1 (C), 114.3 (2CH), 114.0 (2CH), 110.8 (CH), 84.8 (C-O), 71.0 (CH), 57.4 (CH), 55.6 (OCH_3_), 52.6 (CO_2_CH_3_). HRMS (ESI): (M + H)^+^, found 474.1665. [C_26_H_23_N_3_O_6_H]^+^ calculated 474.1660.

*rac-(3R,3′R,S-methyl 1-methyl-2-oxo-2′-phenyl-3′-(phenylcarbamoyl)spiro[indoline-3,5′-isoxazolidine]-4′-carboxylate* (**3e**), *rac-(3R,3′S,4′S)-methyl 1-methyl-2-oxo-2′-phenyl-3′-(phenylcarbamoyl)spiro[indoline-3,5′-isoxazolidine]-4′-carboxylate* (**3′e**) and *rac-(3R,3′S,5′R)-methyl 1-methyl-2-oxo-2′-phenyl-3′-(phenylcarbamoyl)spiro[indoline-3,4′-isoxazolidine]-5′-carboxylate* (**4e**) were obtained from (2-oxoindoline-3-ylidene)acetate **1b** (109 mg, 0.5 mmol) and nitrone **2a** (180 mg, 0.75 mmol). 

Isomer **3e**. Yield 169 mg (74%). Colorless solid, m.p. 155–157 °C. ^1^H NMR (400 MHz, CDCl_3_): δ 9.55 (s, 1H, NH), 7.69 (d, *J* = 7.9 Hz, 2H_Ar_), 7.40–7.27 (m, 5H_Ar_), 7.17–7.10 (m, 3H_Ar_), 7.10–7.03 (m, 1H_Ar_), 7.00–6.90 (m, 2H_Ar_), 6.87 (d, *J* = 7.9 Hz, 1H_Ar_), 5.05 (d, *J* = 5.8 Hz, 1H, HC), 4.20 (d, *J* = 5.8 Hz, 1H, HC), 3.37 (s, 3H, CO_2_CH_3_), 3.27 (s, 3H, NCH_3_). ^13^C NMR (101 MHz, CDCl_3_): δ 173.4 (C), 168.7 (C), 167.6 (C), 148.6 (C), 144.3 (C), 137.8 (C), 131.4 (CH), 129.2 (2CH), 129.1 (2CH), 126.1 (CH), 124.7 (CH), 123.7 (CH), 123.2 (CH), 122.9 (C), 120.0 (2CH), 116.0 (2CH), 109.0 (CH), 83.9 (C-O), 71.1 (CH), 59.8 (CH), 52.7 (CO_2_CH_3_), 26.8 (NCH_3_). HRMS (ESI): (M + H)^+^, found 458.1713. [C_26_H_23_N_3_O_5_H]^+^ calculated 458.1711.

Isomers **3′e** and **4e**. Yield: 30 mg (13%). Ratio: 10:1. Yellow oil. ^1^H NMR (400 MHz, CDCl_3_): **3′e**: δ 8.96 (s, 1H, NH), 7.59 (d, *J* = 7.8 Hz, 2H_Ar_), 6.86 (d, *J* = 7.8 Hz, 1H_Ar_), 5.34 (d, *J* = 7.8 Hz, 1H, CH), 4.18 (d, *J* = 7.8 Hz, 1H, CH), 3.61 (s, 3H, CO_2_CH_3_), 3.12 (s, 3H, NCH_3_), the aromatic signals of the isomers overlap. **4e**: δ 9.05 (s, 1H, NH), 5.09 (s, 1H, CH), 4.90 (s, 1H, CH), 3.36 (s, 3H, CH_3_), 3.31 (s, 3H, CH_3_), the aromatic signals of the isomers overlap. ^13^C NMR (101 MHz, CDCl_3_): **3′e**: δ 173.3 (C), 168.6 (C), 167.1 (C), 151.0 (C), 144.8 (C), 137.2 (C), 131.9 (CH), 129.2 (2CH), 129.1 (2CH), 125.7 (CH), 125.0 (CH), 123.4 (CH), 122.9 (CH), 120.8 (C), 120.3 (2CH), 114.1 (2CH), 109.1 (CH), 84.7 (C-O), 70.9 (CH), 57.7 (CH), 52.5 (CO_2_CH_3_), 26.5 (NCH_3_). HRMS (ESI): (M + H)^+^, found 458.1714. [C_26_H_23_N_3_O_5_H]^+^ calculated 458.1711.

*rac-(3R,3′R,4′S)-methyl 3′-((4-chlorophenyl)carbamoyl)-1-methyl-2-oxo-2′-phenylspiro[indoline-3,5′-isoxazolidine]-4′-carboxylate* (**3f**), *rac-(3R,3′S,4′S)-methyl 3′-((4-chlorophenyl)carbamoyl)-1-methyl-2-oxo-2′-phenylspiro[indoline-3,5′-isoxazolidine]-4′-carboxylate* (**3′f**) and *rac-(3S,3′R,5′S)-methyl 3′-((4-chlorophenyl)carbamoyl)-1-methyl-2-oxo-2′-phenylspiro[indoline-3,4′-isoxazolidine]-5′-carboxylate* (**4f**) were obtained from (2-oxoindoline-3-ylidene)acetate **1b** (109 mg, 0.5 mmol) and nitrone **2b** (206 mg, 0.75 mmol). 

Isomer **3f.** Yield 160 mg (65%). Colorless solid, m.p. 153–155 °C. ^1^H NMR (400 MHz, CDCl_3_): δ 9.63 (s, 1H, NHCO), 7.68–7.60 (m, 2H_Ar_), 7.40–7.34 (m, 1H_Ar_), 7.34–7.27 (m, 4H_Ar_), 7.13–7.05 (m, 3H_Ar_), 7.01–6.92 (m, 2H_Ar_), 6.88 (d, *J* = 7.8 Hz, 1H_Ar_), 5.03 (d, *J* = 5.6 Hz, 1H, CH), 4.16 (d, *J* = 5.6 Hz, 1H, CH), 3.38 (s, 3H, CO_2_CH_3_), 3.27 (s, 3H, NCH_3_). ^13^C NMR (101 MHz, CDCl_3_): δ 173.6 (C), 168.7 (C), 167.7 (C), 148.4 (C), 144.3 (C), 136.4 (C), 131.5 (CH), 129.6 (C), 129.2 (2CH), 129.1 (2CH), 126.1 (CH), 123.8 (CH), 123.3 (CH), 122.8 (C), 121.30 (2CH), 116.1 (2CH), 109.1 (CH), 83.9 (C-O), 71.0 (CH), 59.7 (CH), 52.7 (CO_2_CH_3_), 26.8 (NCH_3_). HRMS (ESI): (M + Na)^+^, found 514.1139. [C_26_H_22_ClN_3_O_5_Na]^+^ calculated 514.1140.

Isomers **3′f** and **4f**. Yield: 42 mg (17%). Ratio: 5:1. Yellowish solid. ^1^H NMR (400 MHz, CDCl_3_): **3′f**: δ 8.98 (s, 1H, NHCO), 7.58–7.51 (m, 2H_Ar_), 7.48–7.39 (m, 1H_Ar_), 7.36–7.27 (m, 5H_Ar_), 7.13–6.98 (m, 4H_Ar_), 6.87 (d, *J* = 7.9 Hz, 1H_Ar_), 5.33 (d, *J* = 7.8 Hz, 1H, CH), 4.18 (d, *J* = 7.8 Hz, 1H, CH), 3.60 (s, 3H, CO_2_CH_3_), 3.11 (s, 3H, NCH_3_). **4f**: δ 9.08 (s, 1H, NHCO), 5.08 (s, 1H, CH), 4.89 (s, 1H, CH), 3.35 (s, 3H, CH_3_), 3.31 (s, 3H, CH_3_), the aromatic signals of the isomers overlap. ^13^C NMR (101 MHz, CDCl_3_): **3′f**: δ 173.2 (C), 168.7 (C), 167.3 (C), 151.0 (C), 144.8 (C), 135.8 (C), 132.0 (CH), 130.1 (C), 129.2 (2CH), 129.2 (2CH), 125.6 (CH), 123.5 (CH), 123.0 (CH), 121.6 (2CH), 120.7 (C), 114.0 (2CH), 109.2 (CH), 84.8 (C-O), 70.8 (CH), 57.7 (CH), 52.5 (CO_2_CH_3_), 26.5 (NCH_3_). HRMS (ESI): (M + Na)^+^, found 514.1139. [C_26_H_22_ClN_3_O_5_Na]^+^ calculated 514.1140

*rac-(3R,3′R,4′S)-methyl 1-methyl-2-oxo-2′-phenyl-3′-(p-tolylcarbamoyl)spiro[indoline-3,5′-isoxazolidine]-4′-carboxylate* (**3g**), *rac-(3R,3′S,4′S)-methyl 1-methyl-2-oxo-2′-phenyl-3′-(p-tolylcarbamoyl)spiro[indoline-3,5′-isoxazolidine]-4′-carboxylate* (**3′g**) and *rac-(3R,3′S,5′R)-methyl 1-methyl-2-oxo-2′-phenyl-3′-(p-tolylcarbamoyl)spiro[indoline-3,4′-isoxazolidine]-5′-carboxylate* (**4g**) were obtained from (2-oxoindoline-3-ylidene)acetate **1b** (109 mg, 0.5 mmol) and nitrone **2c** (191 mg, 0.75 mmol). 

Isomer **3g**. Yield 130 mg (55%). Colorless solid, m.p. 143–146 °C. ^1^H NMR (400 MHz, CDCl_3_): δ 9.44 (s, NHCO), 7.60–7.52 (m, 2H_Ar_), 7.39–7.32 (m, 1H_Ar_), 7.32–7.27 (m, 2H_Ar_), 7.17–7.03 (m, 5H_Ar_), 6.99–6.89 (m, 2H_Ar_), 6.87 (d, *J* = 7.9 Hz, 1H_Ar_), 5.05 (d, *J* = 6.0 Hz, 1H, CH), 4.21 (d, *J* = 6.0 Hz, 1H, CH), 3.37 (s, 3H, CO_2_CH_3_), 3.27 (s, 3H, NCH_3_), 2.32 (s, 3H, H_3_C-C_6_H_4_). ^13^C NMR (101 MHz, CDCl_3_): δ 173.3 (C), 168.7 (C), 167.4 (C), 148.7 (C), 144.3 (C), 135.2 (C), 134.3 (C), 131.4 (CH), 129.6 (2CH), 129.2 (2CH), 126.2 (CH), 123.6 (CH), 123.2 (CH), 123.0 (C), 120.1 (2CH), 116.0 (2CH), 108.9 (CH), 84.0 (C-O), 71.0 (CH), 59.9 (CH), 52.7 (CO_2_CH_3_), 26.8 (NCH_3_), 21.1 (H_3_C-C_6_H_4_). HRMS (ESI): (M + Na)^+^, found 494.1688. [C_27_H_25_N_3_O_5_Na]^+^ calculated 494.1686.

Isomers **3′g** and **4g**. Yield: 47 mg (20%). Ratio 2:1. Beige solid. ^1^H NMR (400 MHz, CDCl_3_): **3′g**: δ 8.91 (s, 1H, NH), 5.34 (d, *J* = 7.8 Hz, 1H, CH), 4.18 (d, *J* = 7.8 Hz, 1H, CH), 3.61 (s, 3H, CO_2_CH_3_), 3.11 (s, 3H, NCH_3_), 2.33 (s, 3H, H_3_C-C_6_H_4_), the aromatic signals of the isomers overlap. **4g**: δ 9.00 (s, 1H, NH), 6.98–6.91 (m, 1H_Ar_), 5.08 (s, 1H, CH), 4.89 (s, 1H, CH), 3.35 (s, 3H, CH_3_), 3.31 (s, 3H, CH_3_), 2.27 (s, 3H, H_3_C-C_6_H_4_), the aromatic signals of the isomers overlap. ^13^C NMR (101 MHz, CDCl_3_): **3′g**: δ 173.3 (C), 168.5 (C), 166.9 (C), 151.1 (C), 144.7 (C), 134.7 (C), 134.6 (C), 131.8 (CH), 129.6 (2CH), 129.1 (2CH), 125.7 (CH), 123.4 (CH), 122.9 (CH), 120.9 (C), 120.6 (2CH), 114.1 (2CH), 109.1 (CH), 84.7 (C-O), 70.9 (CH), 57.6 (CH), 52.5 (CO_2_CH_3_), 26.5 (NCH_3_), 21.0 (H_3_C-C_6_H_4_). **4g**: δ 171.9 (C), 165.2 (C), 164.3 (C), 150.1 (C), 144.1 (C), 134.7 (C), 133.7 (C), 130.0 (CH), 129.6 (2CH), 129.4 (2CH), 124.5 (C), 124.2 (CH), 123.8 (CH), 122.7 (CH), 120.4 (2CH), 114.4 (2CH), 108.5 (CH), 83.7 (CH), 78.0 (C), 64.3 (CH), 52.3 (CO_2_CH_3_), 27.0 (NCH_3_), 20.9 (H_3_C-C_6_H_4_). HRMS (ESI): (M + Na)^+^, found 472.1868. [C_26_H_23_N_3_O_5_Na]^+^ calculated 472.1867.

*rac-(3R,3′R,4′S)-methyl 3′-((4-methoxyphenyl)carbamoyl)-1-methyl-2-oxo-2′-phenylspiro[indoline-3,5′-isoxazolidine]-4′-carboxylate* (**3h**) and *rac-(3R,3′S,4′S)-methyl 3′-((4-methoxyphenyl)carbamoyl)-1-methyl-2-oxo-2′-phenylspiro[indoline-3,5′-isoxazolidine]-4′-carboxylate* (**3′h**) were obtained from (2-oxoindoline-3-ylidene)acetate **1b** (109 mg, 0.5 mmol) with nitrone **2c** (203 mg, 0.75 mmol). 

Isomer **3h**. Yield 111 mg (58%). Colorless solid, m.p. 145–147 °C ^1^H NMR (400 MHz, CDCl_3_): δ 9.43 (s, 1H, NH), 7.64–7.56 (m, 2H_Ar_), 7.40–7.28 (m, 3H_Ar_), 7.16–7.05 (m, 3H_Ar_), 6.99–6.86 (m, 5H_Ar_), 5.08 (d, *J* = 5.9 Hz, 1H, CH), 4.23 (d, *J* = 5.9 Hz, 1H, CH), 3.82 (s, 3H, OCH_3_), 3.39 (s, 3H, CO_2_CH_3_), 3.29 (s, 3H, NCH_3_). ^13^C NMR (101 MHz, CDCl_3_): δ 173.3 (C), 168.6 (C), 167.2 (C), 156.7 (C), 148.6 (C), 144.3 (C), 131.4 (CH), 130.9 (C), 129.1 (2CH), 126.1 (CH), 123.6 (CH), 123.2 (CH), 123.0 (C), 121.6 (2CH), 115.9 (2CH), 114.3 (2CH), 108.9 (CH), 84.0 (C-O), 70.9 (CH), 59.9 (CH), 55.6 (OCH_3_), 52.6 (CO_2_CH_3_), 26.8 (NCH_3_). HRMS (ESI): (M + Na)^+^, found 510.1638. [C_27_H_25_N_3_O_6_Na]^+^ calculated 510.1636.

Isomer **3′h**. Yield 23 mg (12%). Dark yellow solid, m.p. 119–122 °C. ^1^H NMR (400 MHz, CDCl_3_): δ 8.86 (s, 1H), 7.53–7.47 (m, 2H), 7.45–7.39 (m, 1H), 7.36–7.28 (m, 3H), 7.13–7.00 (m, 4H), 6.92–6.82 (m, 3H), 5.33 (d, *J* = 7.9 Hz, 1H), 4.18 (d, *J* = 7.8 Hz, 1H), 3.80 (s, 3H), 3.61 (s, 3H), 3.11 (s, 3H). ^13^C NMR (101 MHz, CDCl_3_) δ 173.3 (C), 168.6 (C), 166.9 (C), 157.0 (C), 151.1 (C), 144.8 (C), 131.9 (CH), 130.4 (C), 129.1 (2CH), 125.7 (CH), 123.4 (CH), 122.9 (CH), 122.1 (2CH), 120.9 (C), 114.4 (2CH), 114.1 (2CH), 109.1 (CH), 84.7 (C-O), 70.8 (CH), 57.6 (CH), 55.6 (OCH_3_), 52.5 (CO_2_CH_3_), 26.5 (NCH3). HRMS (ESI): (M + Na)^+^, found 510.1638. [C_27_H_25_N_3_O_6_Na]^+^ calculated 510.1636.

#### 3.2.2. General Procedure for Obtaining Cycloadducts **3′**

A mixture of nitrone **2** (1.5 eqv.) and dipolarophile **1** (1 eqv.) was stirred in DCM (10 mL) at room temperature for 7 h. The solvent was removed under reduced pressure. Products were separated by column chromatography (silica gel, hexane:ethyl acetate 3:1). Isomers **3a** (25 mg, 11%), **3b** (20 mg, 17%), and **3f** (34 mg, 14%) were also obtained in corresponding experiments.

*rac-(3R,3′S,4′S)-methyl 2-oxo-2′-phenyl-3′-(phenylcarbamoyl)spiro[indoline-3,5′-isoxazolidine]-4′-carboxylate* (**3′a**) was obtained from (2-oxoindoline-3-ylidene)acetate **1a** (102 mg, 0.5 mmol) and nitrone **2a** (180 mg, 0.75 mmol). Yield 163 mg (73%). Colorless solid, m.p. 165–167 °C. ^1^H NMR (400 MHz, DMSO-*d_6_*): δ 10.72 (s, 1H, NHCO), 10.14 (s, 1H, NHCO), 7.66 (d, *J* = 8.2 Hz, 2H_Ar_), 7.59 (d, *J* = 7.2 Hz, 1H_Ar_), 7.38–7.31 (m, 3H_Ar_), 7.30–7.23 (m, 2H_Ar_), 7.14–7.08 (m, 1H_Ar_), 7.05–6.93 (m, 4H_Ar_), 6.90 (d, *J* = 7.7 Hz, 1H_Ar_), 5.08 (d, *J* = 8.5 Hz, 1H, CH), 4.34 (d, *J* = 8.4 Hz, 1H, CH), 3.39 (s, 3H, CO_2_CH_3_). ^13^C NMR (101 MHz, DMSO-*d_6_*): δ 174.2 (C), 167.5 (C), 166.7 (C), 150.0 (C), 143.0 (C), 138.3 (C), 131.0 (CH), 128.6 (2CH), 128.6 (2CH), 126.5 (CH), 123.9 (CH), 123.4 (C), 122.1 (CH), 121.9 (CH), 120.3 (2CH), 114.6 (2CH), 110.2 (CH), 83.5 (C-O), 68.9 (CH), 56.6 (CH), 51.8 (CO_2_CH_3_). HRMS (ESI): (M + H)^+^, found 444.1559. [C_25_H_21_N_3_O_5_H]^+^ calculated 444.1554.

*rac-(3R,3′S,4′S)-methyl 3′-((4-chlorophenyl)carbamoyl)-2-oxo-2′-phenylspiro[indoline-3,5′-isoxazolidine]-4′-carboxylate* (**3′b**) was obtained from (2-oxoindoline-3-ylidene)acetate **1a** (51 mg, 0.25 mmol) and nitrone **2b** (103 mg, 0.375 mmol). Yield 70 mg (58%). Colorless solid, m.p. 163–165 °C. ^1^H NMR (400 MHz, DMSO-*d*_6_): δ 10.72 (s, 1H, NHCO), 10.31 (s, 1H, NHCO), 7.74–7.67 (m, 2H_Ar_), 7.59–7.53 (m, 1H_Ar_), 7.42–7.37 (m, 2H_Ar_), 7.36–7.31 (m, 1H_Ar_), 7.30–7.22 (m, 2H_Ar_), 7.06–6.94 (m, 4H_Ar_), 6.93–6.86 (m, 1H_Ar_), 5.08 (d, *J* = 8.5 Hz, 1H, CH), 4.34 (d, *J* = 8.5 Hz, 1H, CH), 3.38 (s, 3H, CO_2_CH_3_). ^13^C NMR (101 MHz, DMSO-*d_6_*): δ 174.2 (C), 167.5 (C), 167.0 (C), 150.0 (C), 143.0 (C), 137.3 (C), 131.1 (CH), 128.6 (2CH), 128.6 (2CH), 127.7 (C), 126.5 (CH), 123.4 (C), 122.2 (CH), 122.0 (2CH), 121.9 (CH), 115.6 (2CH), 110.3 (CH), 83.5 (C-O), 69.0 (CH), 56.6 (CH), 51.8 (CO_2_CH_3_). HRMS (ESI): (M + Na)^+^, found 500.0989. [C_25_H_20_ClN_3_O_5_Na]^+^ calculated 500.0984.

*rac-(3R,3′S,4′S)-methyl 3′-((4-chlorophenyl)carbamoyl)-1-methyl-2-oxo-2′-phenylspiro[indoline-3,5′-isoxazolidine]-4′-carboxylate (***3′f**) was obtained from (2-oxoindoline-3-ylidene)acetate **1b** (109 mg, 0.5 mmol) and nitrone **2b** (206 mg, 0.75 mmol). Yield 150 mg (61%). Colorless solid, m.p. (dec.) 120–123 °C. ^1^H NMR (400 MHz, CDCl_3_): δ 8.98 (s, 1H, NHCO), 7.58–7.51 (m, 2H_Ar_), 7.48–7.39 (m, 1H_Ar_), 7.36–7.27 (m, 5H_Ar_), 7.13–6.98 (m, 4H_Ar_), 6.87 (d, *J* = 7.9 Hz, 1H_Ar_), 5.33 (d, *J* = 7.8 Hz, 1H, CH), 4.18 (d, *J* = 7.8 Hz, 1H, CH), 3.60 (s, 3H, CO_2_CH_3_), 3.11 (s, 3H, NCH_3_). ^13^C NMR (101 MHz, CDCl_3_): δ 173.2 (C), 168.7 (C), 167.3 (C), 151.0 (C), 144.8 (C), 135.8 (C), 132.0 (CH), 130.1 (C), 129.2 (2CH), 129.2 (2CH), 125.6 (CH), 123.5 (CH), 123.0 (CH), 121.6 (2CH), 120.7 (C), 114.0 (2CH), 109.2 (CH), 84.8 (C-O), 70.8 (CH), 57.7 (CH), 52.5 (CO_2_CH_3_), 26.5 (NCH_3_). HRMS (ESI): (M + Na)^+^, found 514.1138. [C_26_H_22_ClN_3_O_5_Na]^+^ calculated 514.1140.

#### 3.2.3. Procedure for Obtaining Cycloadduct **3′e**

A mixture of nitrone **2a** (360 mg, 1.5 mmol) and dipolarophile **1b** (218 mg, 1 mmol) was stirred in toluene at 55 °C for 7 h. The solvent was removed under reduced pressure. Products were separated by column chromatography (silica gel, hexane:ethyl acetate 3:1). Isomer **3e** was also obtained in the reaction in 37% yield (170 mg).

*rac-(3R,3′S,4′S)-methyl 1-methyl-2-oxo-2′-phenyl-3′-(phenylcarbamoyl)spiro[indoline-3,5′-isoxazolidine]-4′-carboxylate* (**3′e**). Yield 250 mg (54%). Yellow solid, m.p. (dec.) 117–120 °C. ^1^H NMR (400 MHz, CDCl_3_): δ 8.97 (s, 1H, NH), 7.63–7.55 (m, 2H_Ar_), 7.47–7.40 (m, 1H_Ar_), 7.39–7.28 (m, 5H_Ar_), 7.20–7.00 (m, 5H_Ar_), 6.86 (d, *J* = 7.9 Hz, 1H_Ar_), 5.35 (d, *J* = 7.9 Hz, 1H, CH), 4.19 (d, *J* = 7.9 Hz, 1H, CH), 3.61 (s, 3H, CO_2_CH_3_), 3.11 (s, 3H, NCH_3_). ^13^C NMR (101 MHz, CDCl_3_): δ 173.3 (C), 168.6 (C), 167.1 (C), 151.0 (C), 144.8 (C), 137.2 (C), 131.9 (CH), 129.2 (2CH), 129.1 (2CH), 125.7 (CH), 125.0 (CH), 123.4 (CH), 122.9 (CH), 120.8 (C), 120.3 (2CH), 114.1 (2CH), 109.1 (CH), 84.7 (C-O), 70.9 (CH), 57.7 (CH), 52.5 (CO_2_CH_3_), 26.5 (NCH_3_). HRMS (ESI): (M + H)^+^, found 458.1714. [C_26_H_23_N_3_O_5_H]^+^ calculated 458.1711.

#### 3.2.4. General Procedure for Obtaining Cycloadducts **6**

A mixture of nitrone **5** (1.5 eqv.) and dipolarophile **1** (1 eqv.) was stirred in toluene at 110 °C for 14 h. The solvent was removed under reduced pressure. The products were purified by column chromatography (silica gel, hexane:ethyl acetate 3:1). 

*rac-(3R,4′S)-trimethyl 2-oxo-2′-phenylspiro[indoline-3,5′-isoxazolidine]-3′,3′,4′-tricarboxylate* (**6a**) was obtained from the reaction of **1a** (102 mg, 0.5 mmol) with nitrone **5a** (178 mg, 0.75 mmol). Yield 210 mg (95%). Colorless solid, m.p. 156–158 °C. ^1^H NMR (400 MHz, CDCl_3_): δ 8.30 (s, 1H, NH), 7.96 (d, *J* = 7.8 Hz, 1H_Ar_), 7.48 (d, *J* = 7.8 Hz, 2H_Ar_), 7.32–7.21 (m, 3H_Ar_), 7.18–7.10 (m, 1H_Ar_), 7.09–7.01 (m, 1H_Ar_), 6.90 (d, *J* = 7.8 Hz, 1H_Ar_), 5.28 (s, 1H, CH), 3.84 (s, 3H, CH_3_), 3.39 (s, 3H, CH_3_), 3.25 (s, 3H, CH_3_). ^13^C NMR (101 MHz, CDCl_3_): δ 172.6 (C), 167.0 (C), 166.6 (C), 166.3 (C), 145.5 (C), 141.0 (C), 130.7 (CH), 128.4 (2CH), 127.9 (CH), 126.5 (C), 126.2 (CH), 123.5 (CH), 121.3 (2CH), 110.1 (CH), 82.1 (C-O), 78.7 (C(CO_2_CH)_2_), 61.5 (CH), 53.2 (CO_2_CH_3_), 53.0 (CO_2_CH_3_), 52.3 (CO_2_CH_3_). HRMS (ESI): (M + H)^+^, found 441.1297. [C_22_H_20_N_2_O_8_H]^+^ calculated 441.1292.

*rac-(3R,4′S)-trimethyl 2-oxo-2′-(p-tolyl)spiro[indoline-3,5′-isoxazolidine]-3′,3′,4′-tricarboxylate* (**6b**) was obtained from the reaction of **1a** (102 mg, 0.5 mmol) with nitrone **5b** (188 mg, 0.75 mmol). Yield 150 mg (66%). Colorless solid, m.p. 98–100 °C. ^1^H NMR (400 MHz, CDCl_3_): δ 7.96 (dd, *J* = 7.7, 1.2 Hz, 1H_Ar_), 7.85 (s, 1H_,_ NH), 7.41–7.34 (m, 2H_Ar_), 7.25 (m, 1H_Ar_), 7.11–7.00 (m, 3H_Ar_), 6.85 (d, *J* = 7.7 Hz, 1H_Ar_), 5.26 (s, 1H, CH), 3.82 (s, 3H, CH_3_), 3.42 (s, 3H, CH_3_), 3.23 (s, 3H, CH_3_), 2.29 (s, 3H, H_3_C-C_6_H_4_). ^13^C NMR (101 MHz, CDCl_3_): δ 172.9 (C), 167.1 (C), 166.6 (C), 166.4 (C), 142.8 (C), 141.1 (C), 136.2 (C), 130.6 (CH), 128.9 (2CH), 127.9 (CH), 126.6 (C), 123.4 (CH), 121.6 (2CH), 110.2 (CH), 82.0 (C-O), 78.5 (C(CO_2_CH_3_)_2_), 61.5 (CH), 53.1 (CO_2_CH_3_), 53.0 (CO_2_CH_3_), 52.3 (CO_2_CH_3_), 21.1 (H_3_C-C_6_H_4_). HRMS (ESI): (M + Na)^+^, found 477.1271. [C_23_H_22_N_2_O_8_Na]^+^ calculated 477.1268.

*rac-(3R,4′S)-trimethyl 2′-(4-methoxyphenyl)-2-oxospiro[indoline-3,5′-isoxazolidine]-3′,3′,4′-tricarboxylate* (**6c**) was obtained from the reaction of **1a** (102 mg, 0.5 mmol) with nitrone **5c** (200 mg, 0.75 mmol). Yield 170 mg (72%). Pale yellow solid, m.p. 164–167 °C (recrystallized from Et_2_O). ^1^H NMR (400 MHz, CDCl_3_): δ 8.62 (s, 1H, NH), 7.95 (dd, *J* = 7.7, 1.3 Hz, 1H_Ar_), 7.50–7.43 (m, 2H_Ar_), 7.29–7.20 (m, 1H_Ar_), 7.09–7.00 (m, 1H_Ar_), 6.90 (d, *J* = 7.7 Hz, 1H_Ar_), 6.84–6.77 (m, 2H_Ar_), 5.26 (s, 1H, CH), 3.81 (s, 3H, CH_3_), 3.76 (s, 3H, CH_3_), 3.44 (s, 3H, CH_3_), 3.22 (s, 3H, CH_3_). ^13^C NMR (101 MHz, CDCl_3_): δ 172.9 (C), 167.1 (C), 166.6 (C), 166.4 (C), 158.4 (C), 141.1 (C), 138.2 (C), 130.6 (CH), 127.9 (CH), 126.6 (C), 123.9 (2CH), 123.4 (CH), 113.5 (2CH), 110.2 (CH), 82.0 (C-O), 78.5 (C(CO_2_CH)_2_), 61.4 (CH), 55.6 (CH_3_), 53.1 (CH_3_), 53.0 (CH_3_), 52.2 (CH_3_). HRMS (ESI): (M + H)^+^, found 471.1403. [C_23_H_22_N_2_O_9_H]^+^ calculated 471.1398.

*rac-(3R,4′S)-trimethyl 1-methyl-2-oxo-2′-phenylspiro[indoline-3,5′-isoxazolidine]-3′,3′,4′-tricarboxylate* (**6d**) was obtained from the reaction of **1b** (109 mg, 0.5 mmol) with nitrone **5a** (178 mg, 0.75 mmol). Yield 209 mg (92%). Colorless solid, m.p. 139–141 °C. ^1^H NMR (400 MHz, CDCl_3_): δ 7.96 (d, *J* = 7.7 Hz, 1H_Ar_), 7.46 (d, *J* = 7.7 Hz, 2H_Ar_), 7.36–7.22 (m, 3H_Ar_), 7.17–7.03 (m, 2H_Ar_), 6.81 (d, *J* = 7.8 Hz, 1H_Ar_), 5.27 (s, 1H, CH), 3.84 (s, 3H, CH_3_), 3.40 (s, 3H, CH_3_), 3.27 (s, 3H, CH_3_), 3.21 (s, 3H, CH_3_). ^13^C NMR (101 MHz, CDCl_3_): δ 170.6 (C), 167.0 (C), 166.7 (C), 166.1 (C), 145.5 (C), 144.0 (C), 130.6 (CH), 128.4 (2CH), 127.5 (CH), 126.2 (C), 126.1 (CH), 123.5 (CH), 121.2 (2CH), 108.0 (CH), 81.8 (C-O), 78.7 (C(CO_2_CH)_2_), 61.4 (CH), 53.1 (CO_2_CH_3_), 53.0 (CO_2_CH_3_), 52.2 (CO_2_CH_3_), 26.9 (NCH_3_). HRMS (ESI): (M + H)^+^, found 455.1454. [C_23_H_22_N_2_O_8_H]^+^ calculated 455.1449.

*rac-(3R,4′S)-trimethyl 1-methyl-2-oxo-2′-(p-tolyl)spiro[indoline-3,5′-isoxazolidine]-3′,3′,4′-tricarboxylate (***6e**) was obtained from the reaction of **1b** (54 mg, 0.25 mmol) with nitrone **5b** (94 mg, 0.375 mmol). Yield 90 mg (77%). Amber solid, m.p. 108–111 °C. ^1^H NMR (400 MHz, CDCl_3_): δ 7.96 (dd, *J* = 7.7, 1.3 Hz, 1H_Ar_), 7.39–7.27 (m, 3H_Ar_), 7.11–7.02 (m, 3H_Ar_), 6.79 (d, *J* = 7.7 Hz, 1H_Ar_), 5.25 (s, 1H, CH), 3.82 (s, 3H, CO_2_CH_3_), 3.43 (s, 3H, CO_2_CH_3_), 3.26 (s, 3H, CO_2_CH_3_), 3.19 (s, 3H, NCH_3_). ^13^C NMR (101 MHz, CDCl_3_): δ 170.7 (C), 167.1 (C), 166.7 (C), 166.2 (C), 144.0 (C), 142.9 (C), 136.0 (C), 130.5 (CH), 128.9 (2CH), 127.5 (CH), 126.2 (C), 123.4 (CH), 121.5 (2CH), 108.0 (CH), 81.7 (C-O), 78.6 (C(CO_2_Me)_2_), 61.4 (CH), 53.0 (CO_2_CH_3_), 53.0 (CO_2_CH_3_), 52.1 (CO_2_CH_3_), 26.9 (NCH_3_), 21.1 (H_3_C-C_6_H_4_). HRMS (ESI): (M + H)^+^, found 469.1608. [C_24_H_24_N_2_O_8_H]^+^ calculated 469.1605. Appropriate crystals for X-ray analysis were obtained from hexane/ethyl acetate solution. Crystallographic data for **6e** have been deposited with the Cambridge Crystallographic Data Centre, no. CCDC 2177790.

*rac-(3R,4′S)-trimethyl 2′-(4-methoxyphenyl)-1-methyl-2-oxospiro[indoline-3,5′-isoxazolidine]-3′,3′,4′-tricarboxylate (***6f**) was obtained from the reaction of **1b** (109 mg, 0.5 mmol) with nitrone **5c** (200 mg, 0.75 mmol). Yield 199 mg (82%). Pale orange solid, m.p. 151–153 °C. ^1^H NMR (400 MHz, CDCl_3_): δ 7.96 (dd, *J* = 7.5, 1.3 Hz, 1H_Ar_), 7.49–7.41 (m, 2H_Ar_), 7.35–7.27 (m, 1H_Ar_), 7.11–7.02 (m, 1H_Ar_), 6.84–6.75 (m, 3H_Ar_), 5.25 (s, 1H, CH), 3.80 (s, 3H, CH_3_), 3.76 (s, 3H, CH_3_), 3.44 (s, 3H, CH_3_), 3.25 (s, 3H, CH_3_), 3.18 (s, 3H, NCH_3_). ^13^C NMR (101 MHz, CDCl_3_): δ 170.7 (C), 167.2 (C), 166.7 (C), 166.3 (C), 158.3 (C), 144.0 (C), 138.3 (C), 130.6 (CH), 127.5 (CH), 126.2 (C), 123.8 (2CH), 123.4 (CH), 113.5 (2CH), 108.0 (CH), 81.6 (C-O), 78.6 (C(CO_2_CH)_2_), 61.2 (CH), 55.5 (CH_3_), 53.0 (CH_3_), 53.0 (CH_3_), 52.1 (CH_3_), 26.8 (NCH_3_). HRMS (ESI): (M + H)^+^, found 485.1558. [C_24_H_24_N_2_O_9_H]^+^ calculated 485.1555.

#### 3.2.5. General Procedure for Obtaining 1,3-Aminoalcohols **7** and **7′**

Activated zinc dust and glacial acetic acid were added to the solution of isoxazolidine **3** (or **3′**) in methanol. The mixture was stirred at room temperature for 1 h. Then, the zinc dust was filtered out. The filtrate was neutralized with saturated NaHCO_3_ solution until pH = 8. The resulting solution was extracted with DCM (2 × 100 mL). Organic layers were combined and dried with anhydrous sodium sulfate. The solvent was removed under reduced pressure. The product was purified by column chromatography (silica gel, hexane:ethyl acetate 2:1).

*rac-(2R,3S)-methyl 2-((R)-3-hydroxy-1-methyl-2-oxoindolin-3-yl)-4-oxo-3,4-bis(phenylamino)butanoate (***7a**) was obtained from the reaction of **3e** (125 mg, 0.27 mmol) with 390 mg of zinc dust and 0.9 mL of glacial acetic acid in methanol (4 mL). Yield 72 mg (58%). Colorless oil. ^1^H NMR (400 MHz, C_6_D_6_): δ 9.03 (s, 1H, NHCO), 7.59–7.51 (m, 2H_Ar_), 7.44 (d, *J* = 7.7 Hz, 1H_Ar_), 7.14–6.99 (m, 5H_Ar_), 6.96–6.85 (m, 2H_Ar_), 6.79–6.69 (m, 2H_Ar_), 6.69–6.61 (m, 2H_Ar_), 6.14 (d, *J* = 7.7 Hz, 1H_Ar_), 5.95 (s, 1H, OH), 5.84 (d, *J* = 9.7 Hz, 1H, NHPh), 5.01 (dd, *J* = 9.7, 3.5 Hz, 1H, CH), 3.92 (d, *J* = 3.5 Hz, 1H, CH), 3.12 (s, 3H, CO_2_CH_3_), 2.51 (s, 3H, NCH_3_). ^13^C NMR (101 MHz, C_6_D_6_): δ 175.6 (C), 173.7 (C), 169.7 (C), 147.0 (C), 143.5 (C), 138.6 (C), 131.5 (C), 129.8 (CH), 129.8 (2CH), 129.1 (2CH), 124.4 (CH), 124.3 (CH), 123.1 (CH), 120.2 (2CH), 120.1 (CH), 115.3 (2CH), 108.9 (CH), 76.8 (C-OH), 60.1 (CH), 52.0 (CO_2_CH_3_), 50.8 (CH), 25.8 (NCH_3_). HRMS (ESI): (M + H)^+^, found 460.1872. [C_26_H_25_N_3_O_5_H]^+^ calculated 460.1867.

*rac-(2R,3R)-methyl 2-((R)-3-hydroxy-1-methyl-2-oxoindolin-3-yl)-4-oxo-3,4-bis(phenylamino)butanoate* (**7′a**) was obtained from the reaction of **3′e** (50 mg, 0.11 mmol) with 130 mg of zinc dust and 0.3 mL of glacial acetic acid in methanol (2 mL). Yield 25 mg (50%). Pale yellow oil. ^1^H NMR (400 MHz, C_6_D_6_): δ 8.38 (s, 1H, NHCO), 7.43–7.36 (m, 2H_Ar_), 7.29 (dd, *J* = 7.8, 1.3 Hz, 1H_Ar_), 7.01–6.94 (m, 2H_Ar_), 6.88–6.69 (m, 5H_Ar_), 6.53–6.46 (m, 1H_Ar_), 5.84–5.74 (m, 3H_Ar_), 5.11 (s, 1H, OH), 4.99 (d, *J* = 9.3 Hz, 1H, NH), 4.84 (d, *J* = 2.9 Hz, 1H, CH), 4.01 (dd, *J* = 9.3, 2.9 Hz, 1H, CH), 3.36 (s, 3H, CO_2_CH_3_), 2.23 (s, 3H, NCH_3_). ^13^C NMR (101 MHz, C_6_D_6_): δ 176.0 (C), 172.2 (C), 169.9 (C), 145.9 (C), 144.6 (C), 138.3 (C), 130.2 (CH), 129.5 (2CH), 129.1 (2CH), 128.4 (C) 124.6 (CH), 124.3 (CH), 123.4 (CH), 119.7 (2CH), 118.8 (CH), 112.4 (2CH), 109.1 (CH), 76.2 (C-OH), 56.8 (CH), 53.4 (CO_2_CH_3_), 52.7 (CH), 25.5 (NCH_3_). HRMS (ESI): (M + H)^+^, found 460.1872. [C_26_H_25_N_3_O_5_H]^+^ calculated 460.1867.

*rac-(2R,3S)-methyl 4-((4-chlorophenyl)amino)-2-((R)-3-hydroxy-1-methyl-2-oxoindolin-3-yl)-4-oxo-3-(phenylamino)butanoate* (**7b**) was obtained from the reaction of **3f** (50 mg, 0.11 mmol) with 130 mg of zinc dust and 0.3 mL of glacial acetic acid in methanol (2 mL). Yield 31 mg (62%). Pale brown oil. ^1^H NMR (400 MHz, C_6_D_6_): δ 8.97 (s, 1H, NHCO), 7.42 (d, *J* = 7.8 Hz, 1H_Ar_), 7.32–7.25 (m, 2H_Ar_), 7.09–7.00 (m, 4H_Ar_), 6.97–6.87 (m, 1H_Ar_), 6.80–6.70 (m, 2H_Ar_), 6.64 (d, *J* = 7.8 Hz, 2H_Ar_), 6.14 (d, *J* = 7.8 Hz, 1H_Ar_), 5.87 (s, 2H, OH, NHPh), 4.95 (s, 1H, CH), 3.89 (d, *J* = 3.0 Hz, 1H, CH), 3.10 (s, 3H, CO_2_CH_3_), 2.50 (s, 3H, NCH_3_). ^13^C NMR (101 MHz, C_6_D_6_): δ 175.4 (C), 174.1 (C), 169.7 (C), 146.9 (C), 143.3 (C), 137.1 (C), 131.6 (C), 129.9 (CH), 129.8 (2CH), 129.3 (C), 129.2 (2CH), 124.3 (CH), 123.1 (CH), 121.3 (2CH), 120.3 (CH), 115.3 (2CH), 109.0 (CH), 76.7 (C-OH), 60.2 (CH), 52.0 (CO_2_CH_3_), 50.2 (CH), 25.8 (NCH_3_). HRMS (ESI): (M + Na)^+^, found 516.1297. [C_26_H_24_ClN_3_O_5_Na]^+^ calculated 516.1297.

#### 3.2.6. Procedure for Obtaining Lactone **8**

Activated zinc dust (130 mg) was added to the solution of isoxazolidine **6d** (50 mg, 0.11 mmol) in glacial acetic acid (2 mL). The mixture was stirred at room temperature for 2 h. Then, the zinc dust was filtered out. The filtrate was neutralized with saturated NaHCO_3_ water solution so that pH = 8. The resulting solution was extracted with DCM (2 × 100 mL). Organic layers were combined and dried over anhydrous sodium sulfate. The solvent was removed under reduced pressure. The product was purified by column chromatography (silica gel, hexane:ethyl acetate 2:1). 

*rac-(2R,3S,4S)-dimethyl 1′-methyl-2′,5-dioxo-4-(phenylamino)-4,5-dihydro-3H-spiro[furan-2,3′-indoline]-3,4-dicarboxylate* (**8**). Yield 25 mg (55%). Pale brown oil. ^1^H NMR (400 MHz, C_6_D_6_): δ 7.83 (dd, *J* = 7.7, 1.2 Hz, 1H_Ar_), 7.21–7.17 (m, 2H_Ar_), 7.12–7.03 (m, 2H_Ar_), 6.94–6.84 (m, 1H_Ar_), 6.82–6.73 (m, 1H_Ar_), 6.72–6.62 (m, 1H_Ar_), 6.08–6.00 (m, 1H_Ar_), 5.89 (s, 1H, NHPh), 4.91 (s, 1H, CH), 3.29 (s, 3H, CO_2_CH_3_), 2.81 (s, 3H, CO_2_CH_3_), 2.41 (s, 3H, NCH3). ^13^C NMR (101 MHz, C_6_D_6_): δ 172.3 (C), 169.6 (C), 167.3 (C), 167.1 (C), 145.0 (C), 144.2 (C), 131.6 (CH), 129.3 (2CH), 127.3 (CH), 123.5 (C), 123.4 (CH), 121.6 (CH), 119.1 (2CH), 108.9 (CH), 81.6 (C-O), 68.3 (C), 55.5 (CO_2_CH_3_), 51.9 (CH), 26.1 (NCH_3_). HRMS (ESI): (M + Na)^+^, found 447.1161. [C_22_H_20_N_2_O_7_Na]^+^ calculated 447.1163. Appropriate crystals for X-ray analysis were obtained from ethanol solution. Crystallographic data for **8** have been deposited with the Cambridge Crystallographic Data Centre, no. CCDC 2177791.

### 3.3. Bioactivity Assay

#### 3.3.1. Cell Culture

MCF7 breast adenocarcinoma, A549 lung cancer, MDA-MB-231 breast adenocarcinoma, Caki-2 kidney clear cell carcinoma, and T98-G glioblastoma cell lines were purchased from the American Type Culture Collection (ATCC, Manassas, VA, USA). The MCF7 cells were maintained in Advanced MEM (Gibco, Paisley, UK) supplemented with 5% fetal bovine serum (FBS, Gibco, UK), penicillin (100 UI/mL), streptomycin (100 µg/mL), insulin (0.01 mg/mL), and GlutaMax (1.87 mM, Gibco, UK). The A549 cells were maintained in F12-K media (Gibco, Grand Island, NY, USA) supplemented with 10% fetal bovine serum (FBS, Origin: Brazil, Gibco, UK), penicillin (100 UI/mL), streptomycin (100 µg/mL), and GlutaMax (2 mM, Gibco, UK). The MDA-MB-231 cells were maintained in Advanced DMEM/F12 (Gibco, USA) supplemented with 5% fetal bovine serum (FBS, Gibco, UK), penicillin (100 UI/mL), streptomycin (100 µg/mL), and GlutaMax (2.5 mM, Gibco, UK). The T98-G cells were maintained in Advanced MEM (Gibco, UK) supplemented with 5% fetal bovine serum (FBS, Origin: Brazil, Gibco, UK), penicillin (100 UI/mL), streptomycin (100 µg/mL), and GlutaMax (1.87 mM, Gibco, UK). All the cell lines were cultivated under a humidified atmosphere of 95% air/5% CO_2_ at 37 °C. Subconfluent monolayers, in the log growth phase, were harvested by a brief treatment with TrypLE Express solution (Gibco, USA) in phosphate-buffered saline (PBS, Capricorn Scientific, Germany) and washed three times with serum-free PBS. The number of viable cells was determined by trypan blue exclusion.

#### 3.3.2. Antiproliferative Assay

The effects of the synthesized compounds on cell viability were determined using the MTT colorimetric test. All the examined cell lines were diluted with the growth medium to 3.5 × 10^4^ cells per mL, and the aliquots (7 × 10^3^ cells per 200 μL) were placed in individual wells in 96-well multiplates (Eppendorf, Germany) and incubated for 24 h. The cells were treated with the synthesized compounds separately at a concentration of 50 μM and incubated for 72 h at 37 °C in a 5% CO_2_ atmosphere. Each compound was tested in triplicate. The cells were treated with 40 μL of an MTT solution (3-(4,5-dimethylthiazol-2-yl)-2,5-diphenyltetrazolium bromide, 5 mg/mL in PBS) and incubated for 8 h. The medium with the MTT was removed and DMSO (150 μL) was added to dissolve the formazan crystals. The plates were shaken for 10 min. The optical density of each well was determined at 560 nm using a GloMax Multi+ (Promega, Madison, WI, USA) microplate reader. The cytotoxicity of each compound was evaluated in three separate experiments.

## 4. Conclusions

It has been shown that the 1,3-dipolar cycloaddition of 2-(2-oxoindoline-3-ylidene)acetates with aldo- and ketonitrones is an effective method for the selective synthesis of new highly functionalized spiroisoxazolidines. The reaction of *C*-carbamoyl aldonitrones predominantly obtains 5-spiroisoxazolidines. In this case, varying the reaction conditions makes it possible to selectively obtain certain diastereomers in good yields due to the reversibility of the reaction. The reaction of *C*,*C*-bis(methoxycarbonyl) ketonitrones obtains only 5-spiroisoxazolidines in good-to-high yields. The reduction of the obtained cycloadducts can obtain aminoalcohols or spirolactones depending on the structure of the starting cycloadduct. Cytotoxicity screening against several cancer cell lines revealed that several cycloadducts exhibit antiproliferative activity.

## Figures and Tables

**Table 1:**
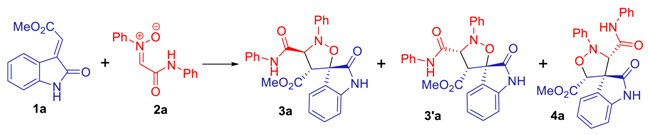
Optimization of cycloaddition reaction conditions.

Entry	Conditions	Ratio 3a:3′a:4a ^1^	Isolated Yield, %		
			3a	3′a	4a
1	CH_3_CN, 82 °C, 14 h	- ^2^	67	-	-
2	PhMe, 80 °C, 14 h	4:1:0.3	70	18	
3	PhMe, 110 °C, 7 h	- ^2^	45	-	-
4	CH_2_Cl_2_, rt, 7 h	1:4:0	11	73	-

^1^—from ^1^H NMR spectra of the crude reaction mixtures; ^2^—analysis of the ^1^H NMR spectrum of the reaction mixture is difficult due to tarring.

**Table 2:**
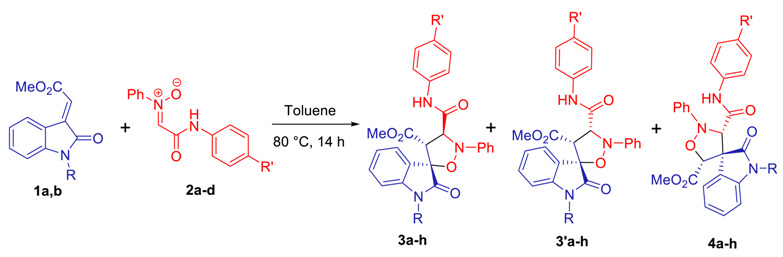
Cycloaddition of 2-(2-oxoindoline-3-ylidene)acetates **1a,b** with *C*-carbamoyl nitrones **2a-d** under heating.

Entry	Dipolarophile	R	Nitrone	R’	Products	Ratio 3a:3′a:4a ^1^	Isolated Yield, %	
							3	(3′ + 4)
1	**1a**	H	**2a**	H	**3a**, **3′a**, **4a**	4:1:0.3	70	18
2	**1a**	H	**2b**	Cl	**3b**, **3′b**, **4b**	5:1:0.2	70	16
3	**1a**	H	**2c**	Me	**3c**, **3′c**, **4c**	5:1:0.5	57	24 ^2^
4	**1a**	H	**2d**	MeO	**3d**, **3′d**, **4d**	4:1:0.3	69	19
5	**1b**	Me	**2a**	H	**3e**, **3′e**, **4e**	5:1:0.2	74	13
6	**1b**	Me	**2b**	Cl	**3f**, **3′f**, **4f**	5:1:0.2	65	17
7	**1b**	Me	**2c**	Me	**3g**, **3′g**, **4g**	3.5:1:0.3	55	20
8	**1b**	Me	**2d**	MeO	**3h**, **3′h**, **4h**	3.2:1:0.2	58	12 ^3^

^1^—from ^1^H NMR spectra of the crude reaction mixtures. ^2^—8% of the (**3′c** + **4c**) mixture and 16% of pure **3′c**. ^3^—pure **3′h**.

**Table 3:**
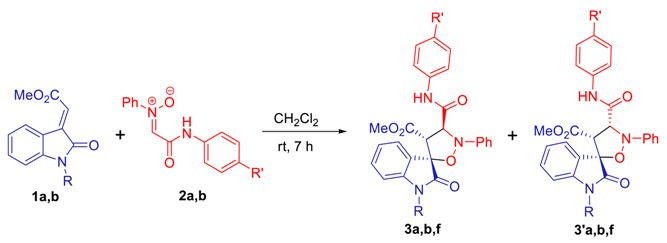
Cycloaddition of 2-(2-oxoindoline-3-ylidene)acetates **1a,b** with *C*-carbamoyl nitrones **2a,b** at rt.

Entry	Dipolarophile	R	Nitrone	R’	Products	Ratio 3a:3′a ^1^	Isolated Yield, %	
							3	3′
1	**1a**	H	**2a**	H	**3a**, **3′a**	1:4	11	73
2	**1a**	H	**2b**	Cl	**3b**, **3′b**	1:4	17	58
3	**1b**	Me	**2b**	Cl	**3f**, **3′f**	1:4	14	61

^1^—from ^1^H NMR spectra of the crude reaction mixtures.

**Table 4:**
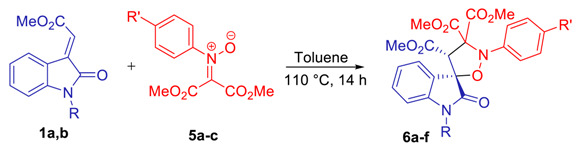
Cycloaddition of 2-(2-oxoindoline-3-ylidene)acetates **1a,b** with ketonitrones **5a–c**.

Entry	Dipolarophile	R	Nitrone	R’	Product	Isolated Yield of 6, %
1	**1a**	H	**5a**	H	**6a**	95
2	**1a**	H	**5b**	Me	**6b**	66
3	**1a**	H	**5c**	MeO	**6c**	72
4	**1b**	Me	**5a**	H	**6d**	92
5	**1b**	Me	**5b**	Me	**6e**	77
6	**1b**	Me	**5c**	MeO	**6f**	82

## Data Availability

Data are contained within the article.

## Supplementary Materials

The following supporting information can be downloaded at: https://www.mdpi.com/article/10.3390/ijms232012639/s1.
