# Supplementary material for: Selective and Reversible 1,3-Dipolar Cycloaddition of 2-(2-Oxoindoline-3-ylidene)acetates with Nitrones in the Synthesis of Functionalized Spiroisoxazolidines
Source: Int J Mol Sci. 2022 Oct 20;23(20):12639. doi: 10.3390/ijms232012639 (PMC9603865; doi:10.3390/ijms232012639)

Supplementary Information

Selective and reversible 1,3-dipolar cycloaddition of 2-(2-oxoindoline-3-ylidene)acetates with nitrones in the synthesis of functionalized spiroisoxazolidines

Dmitriy D. Karcev,[1] Mariia M. Efremova,[1] Alexander P. Molchanov,[1] Nikolai V. Rostovskii,[1*]
Mariya A. Kryukova,[1] Alexander S. Bunev,[2] Dmitry A. Khochenkov[2,3]

[1] Saint Petersburg State University, Institute of Chemistry, Universitetsky pr. 26, Saint Petersburg 198504, Russian Federation
[2] Togliatti State University, Medicinal Chemistry Center, Belorusskaya st. 14, Togliatti 445020, Russian Federation
[3] Blokhin National Medical Center of Oncology, Kashirskoe shosse 24, Moscow 115478, Russian Federation
* Correspondence: n.rostovskiy@spbu.ru

Table of contents

^{1}H and ^{13}C NMR spectra S2

Figure S1. ^{1}H-^{1}H NOESY of **3h** S35

Figure S2. ^{1}H-^{1}H NOESY of **3’h** S35

Figure S3. ^{1}H-^{1}H NOESY of **7a** S36

Figure S4. ^{1}H-^{1}H NOESY of **7'a** S36

Figure S5. ^{1}H-^{1}H NOESY of **8** S37

Figure S6. ^{1}H-^{1}H NOESY of the mixture of **3’c** and **4c** S37

^{1}H and ^{13}C NMR spectra

^{1}H NMR (400 MHz, $CDCl_3$) spectrum of compound **3a**

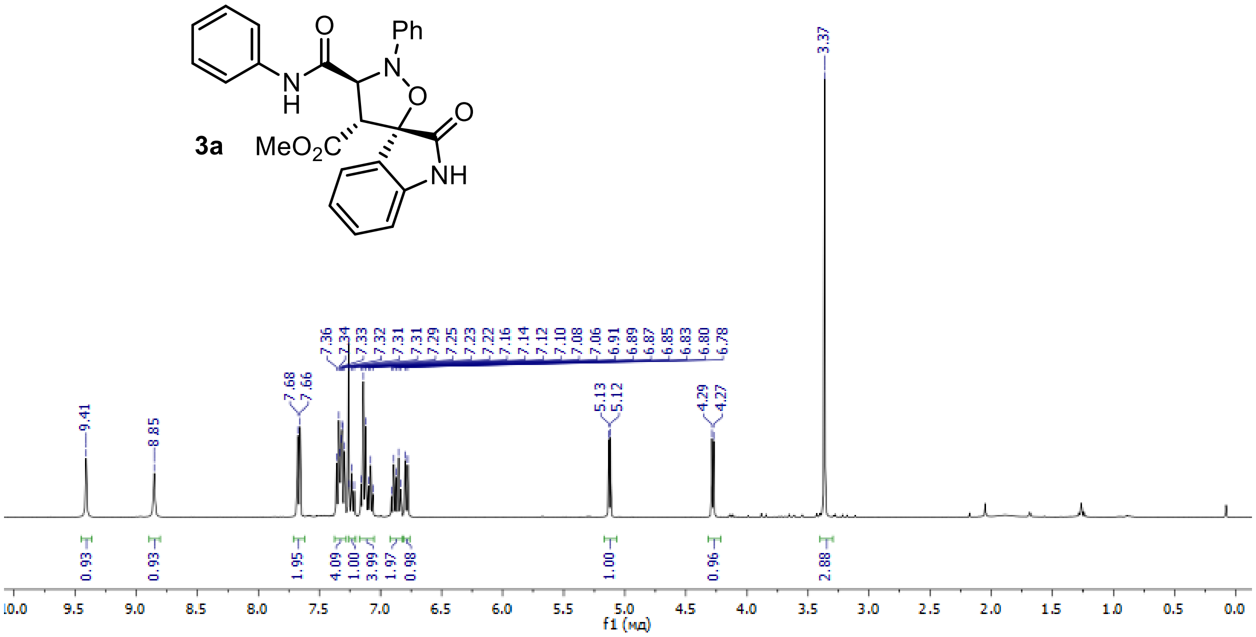

^{13}C NMR (100 MHz, $CDCl_3$) spectrum of compound **3a**

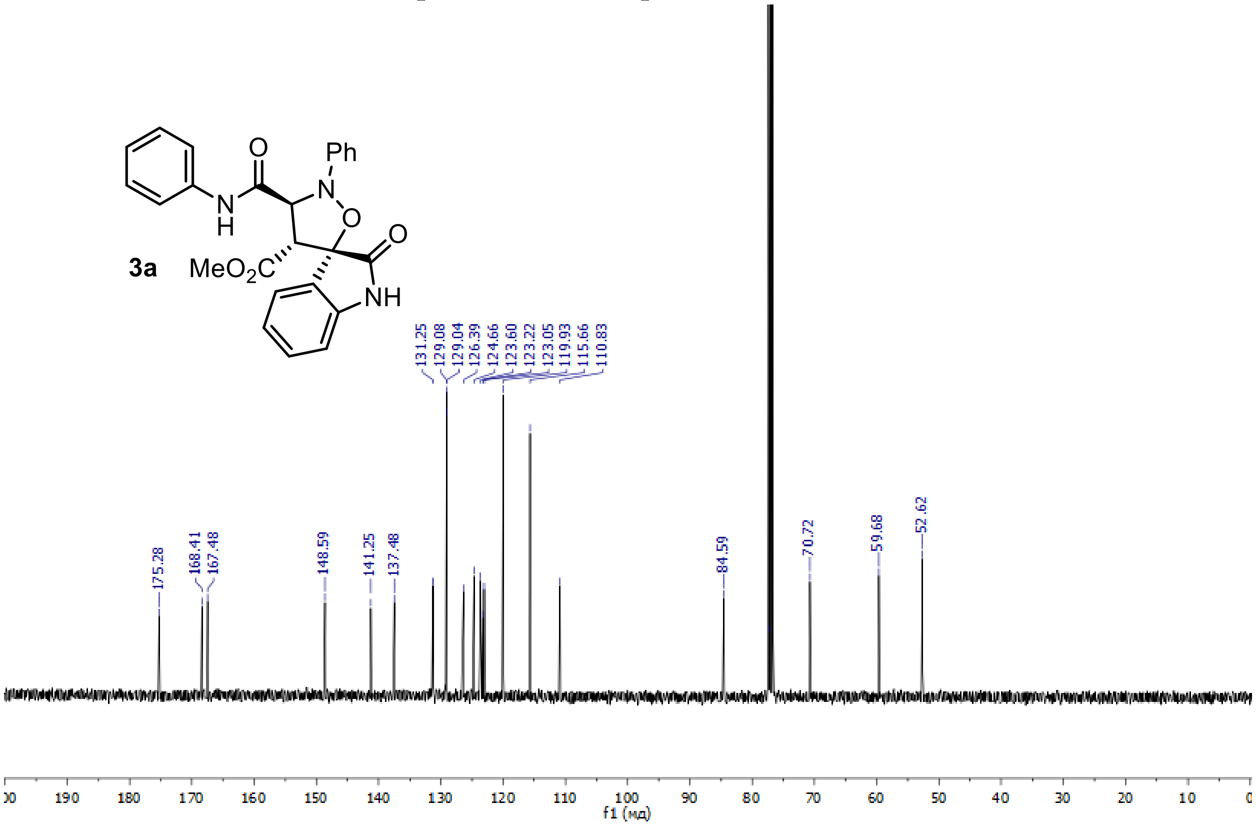

^{1}H NMR (400 MHz, DMSO-d6) spectrum of compound **3'a**

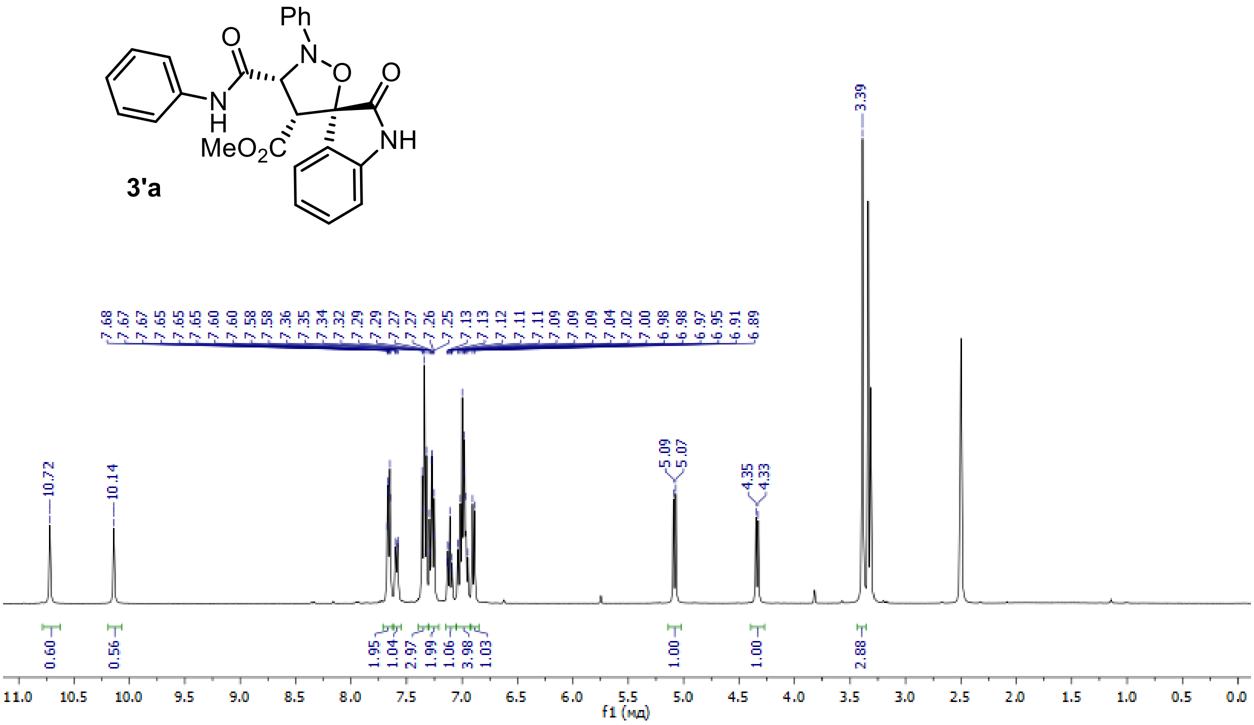

^{13}C NMR (100 MHz, DMSO-d6) spectrum of compound **3'a**

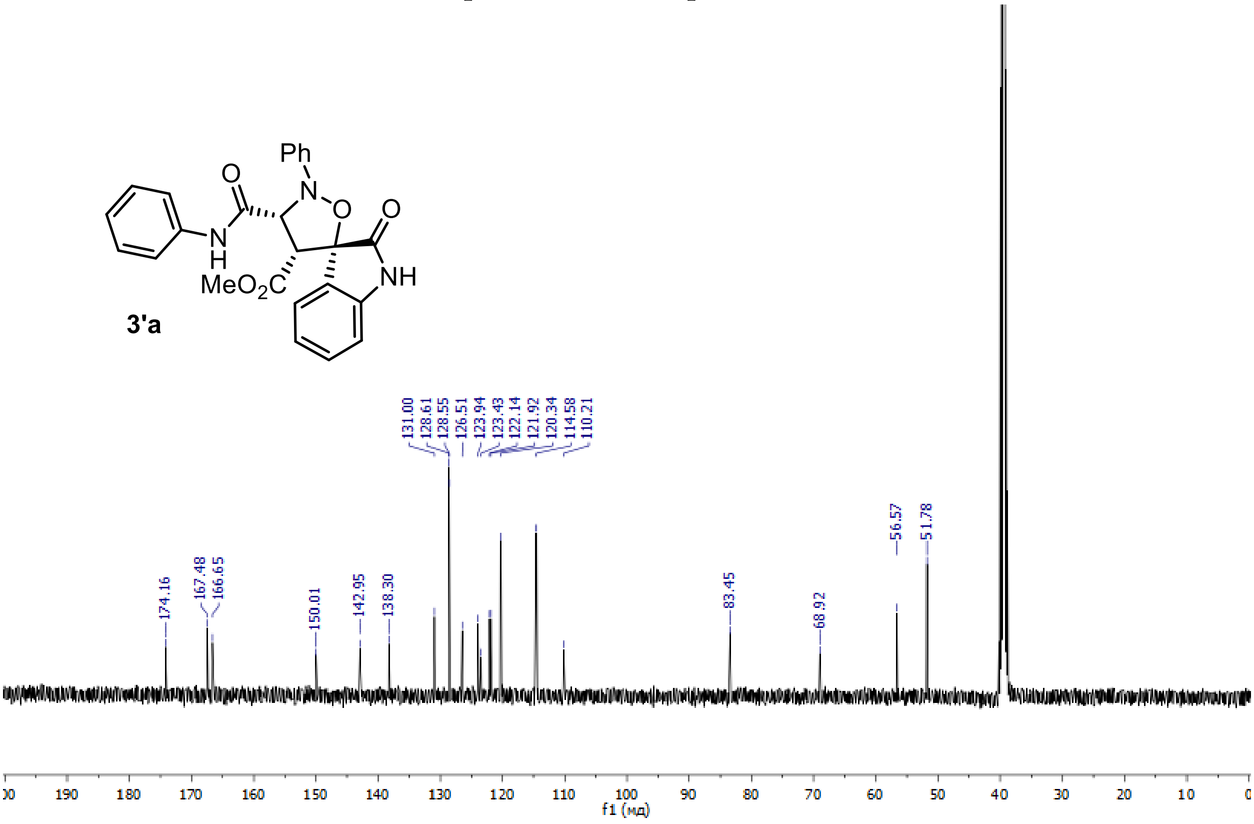

^{1}H NMR (400 MHz, $CDCl_3$) spectrum of the mixture of compounds **3'a** and **4a**

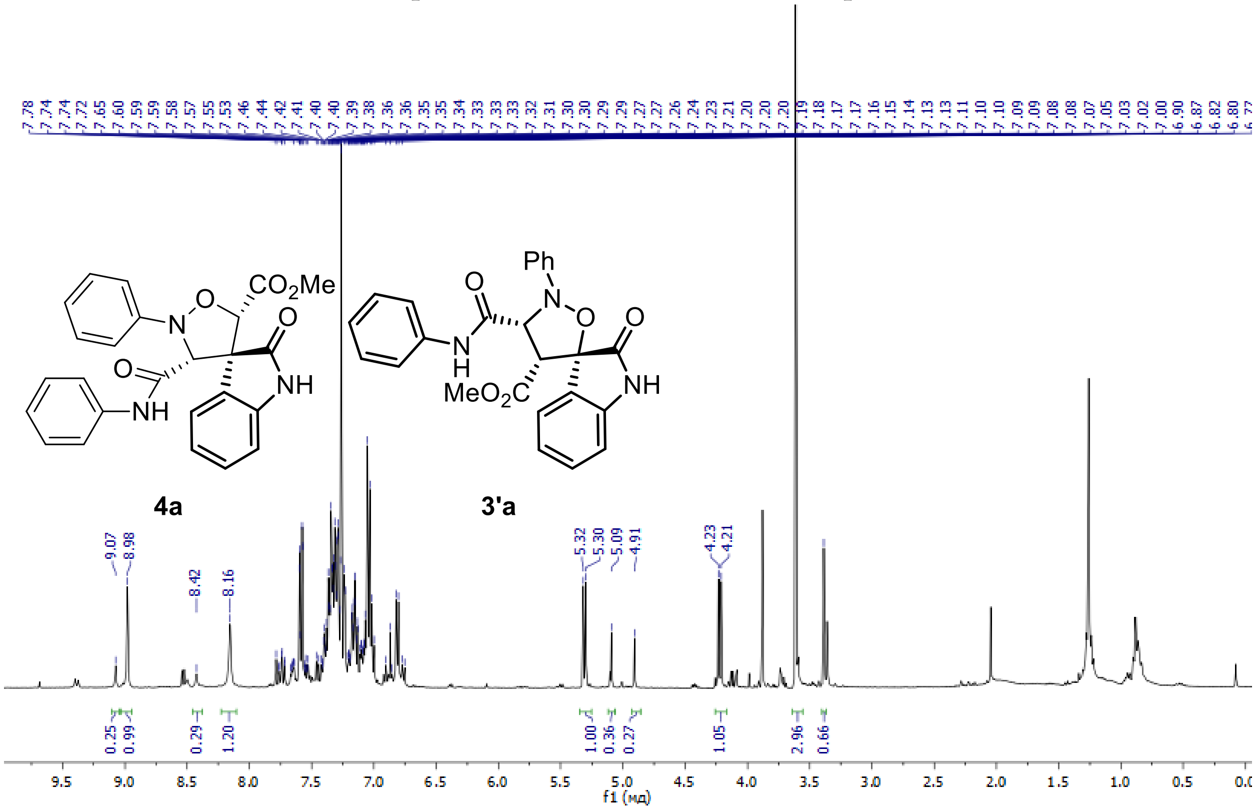

^{1}H NMR (400 MHz, DMSO-d6) spectrum of compound **3b**

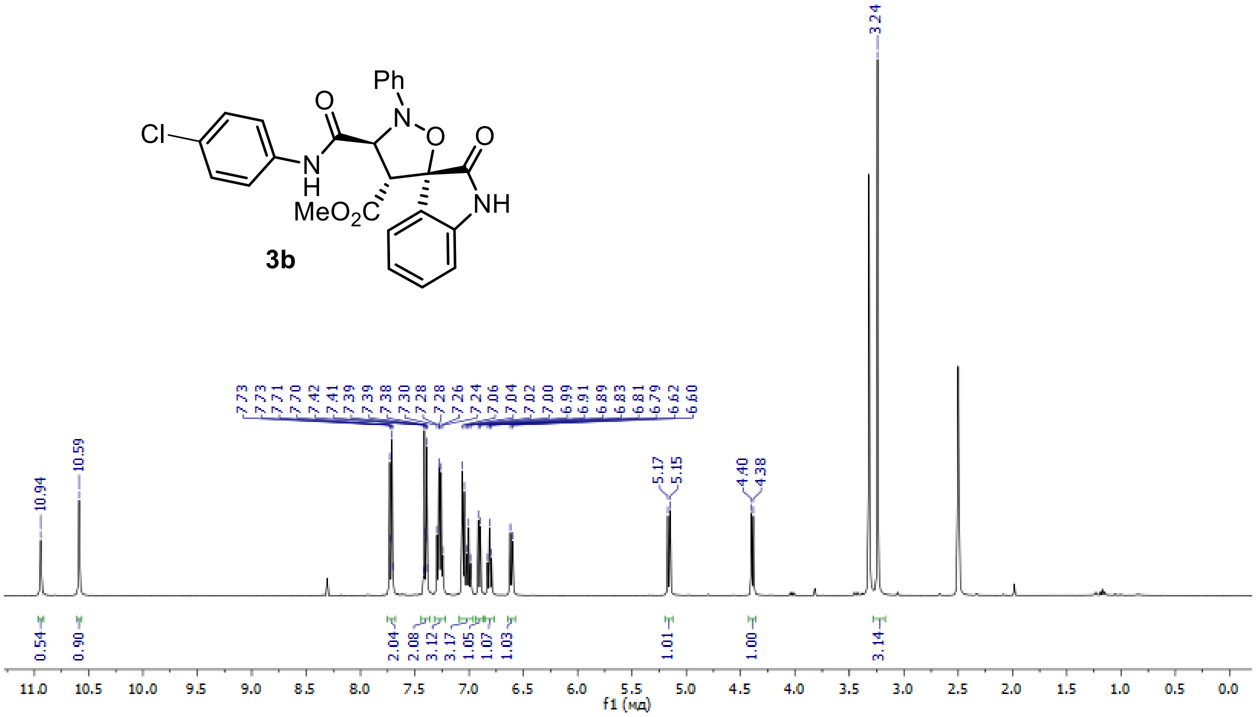

^{13}C NMR (100 MHz, DMSO-d6) spectrum of compound **3b**

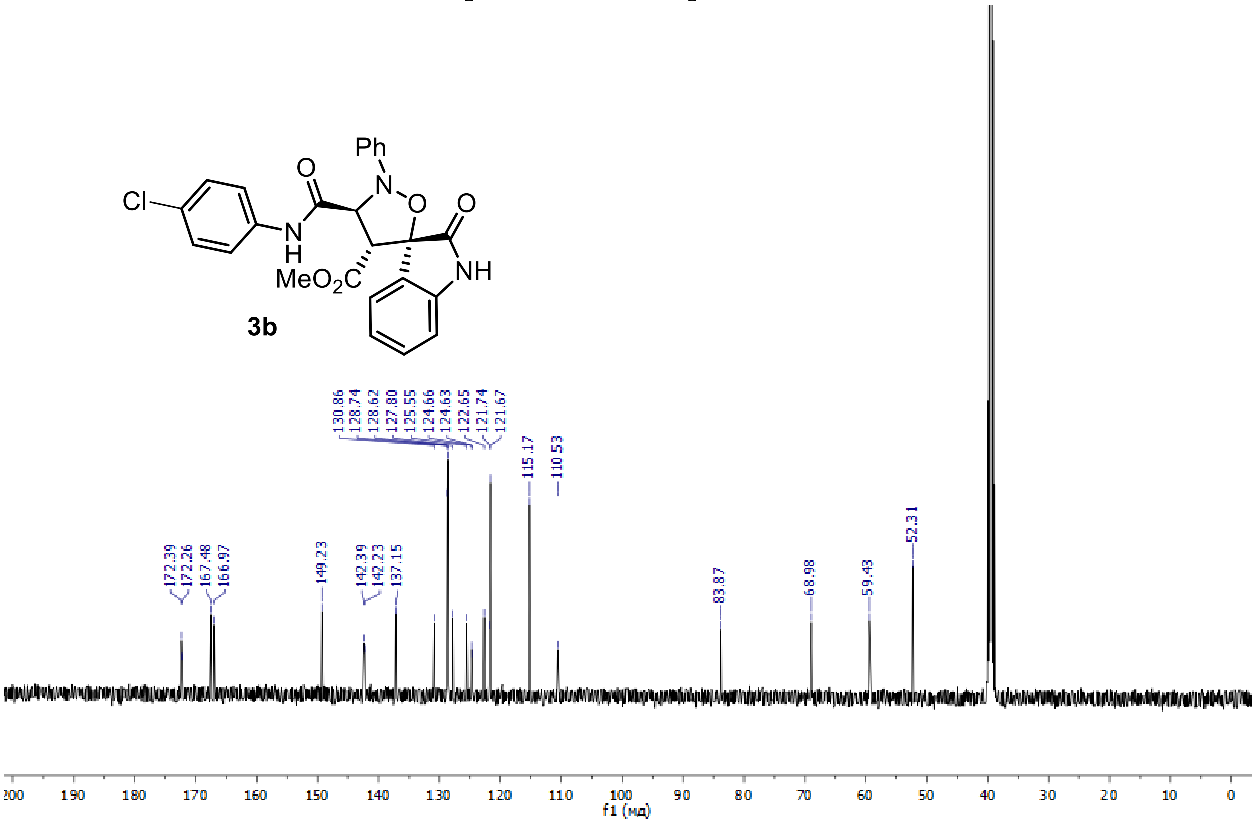

^{1}H NMR (400 MHz, DMSO-d$_6$) spectrum of compound **3'b**

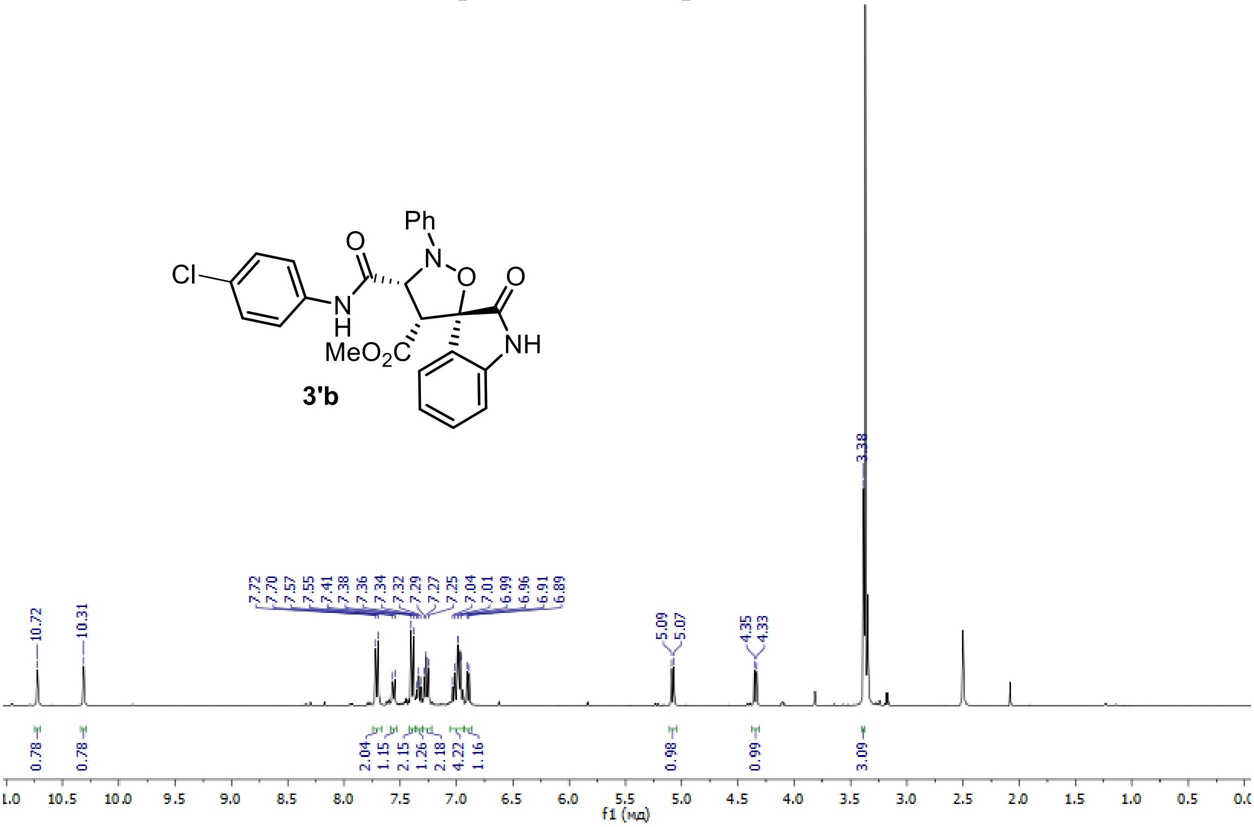

^{13}C NMR (100 MHz, DMSO-d$_6$) spectrum of compound **3'b**

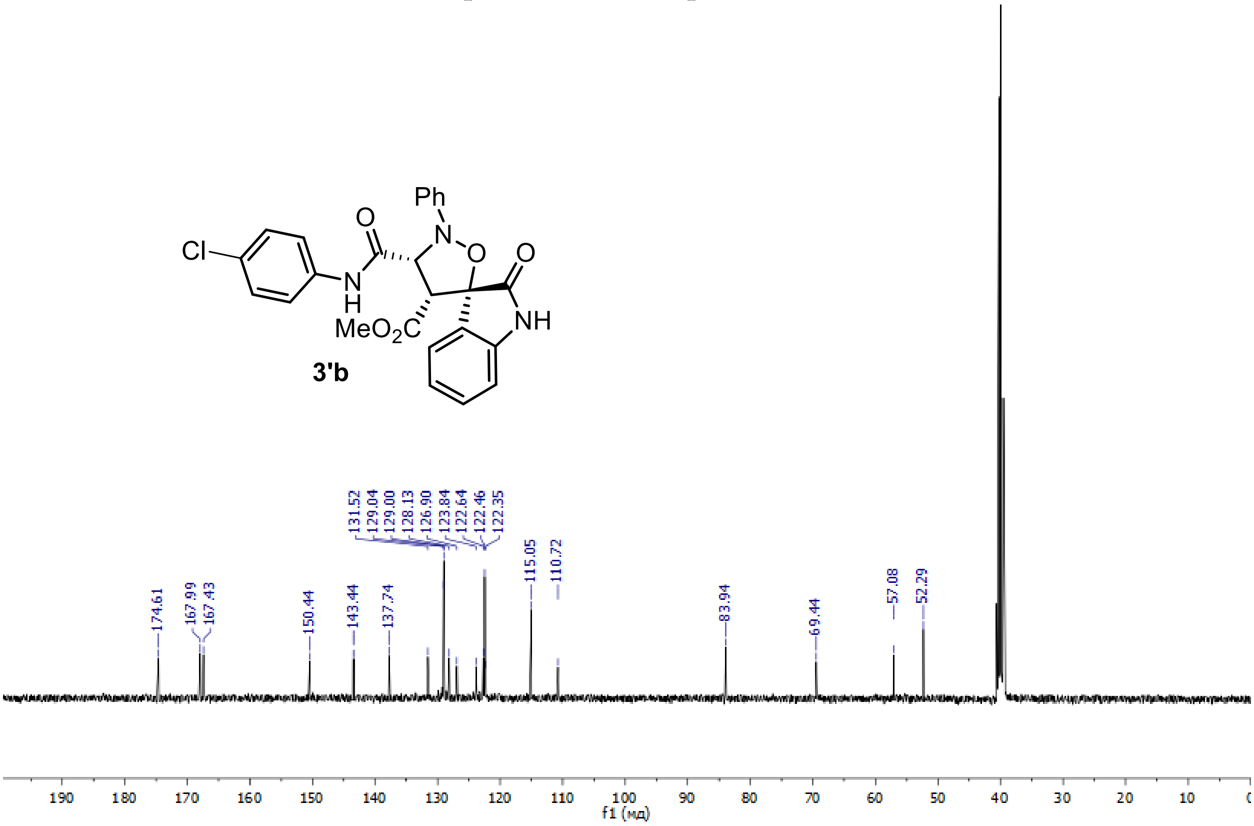

^{1}H NMR (400 MHz, $CDCl_3$) spectrum of the mixture of compounds **3'b** and **4b**

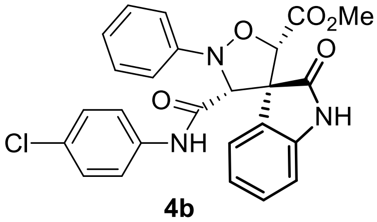

4b

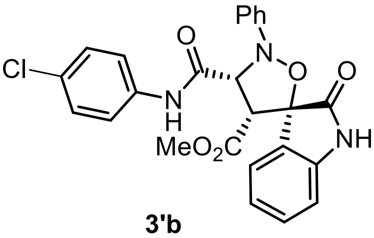

3'b

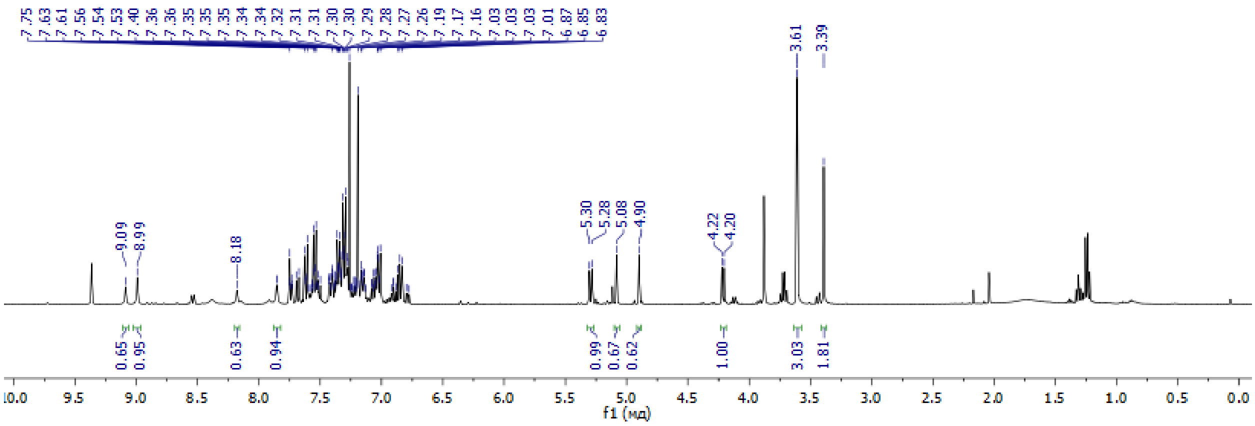

^{1}H NMR (400 MHz, $CDCl_3$) spectrum of compound **3c**

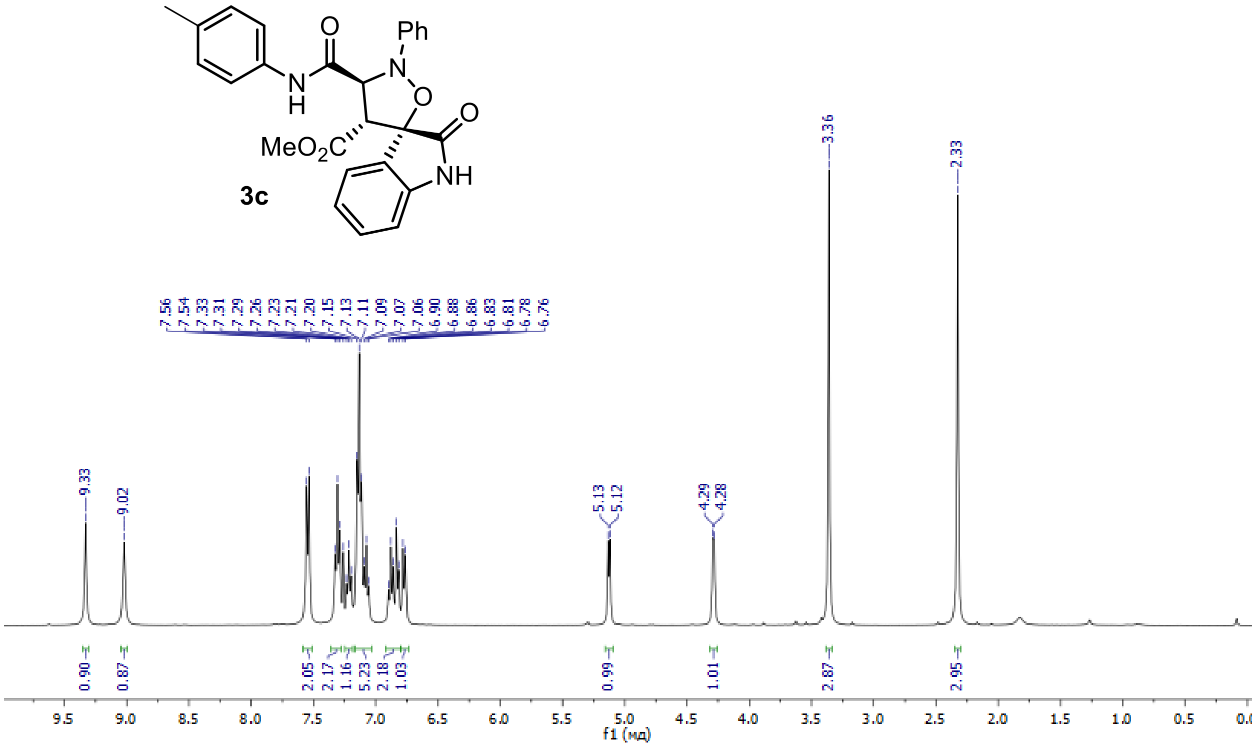

^{13}C NMR (100 MHz, $CDCl_3$) spectrum of compound **3c**

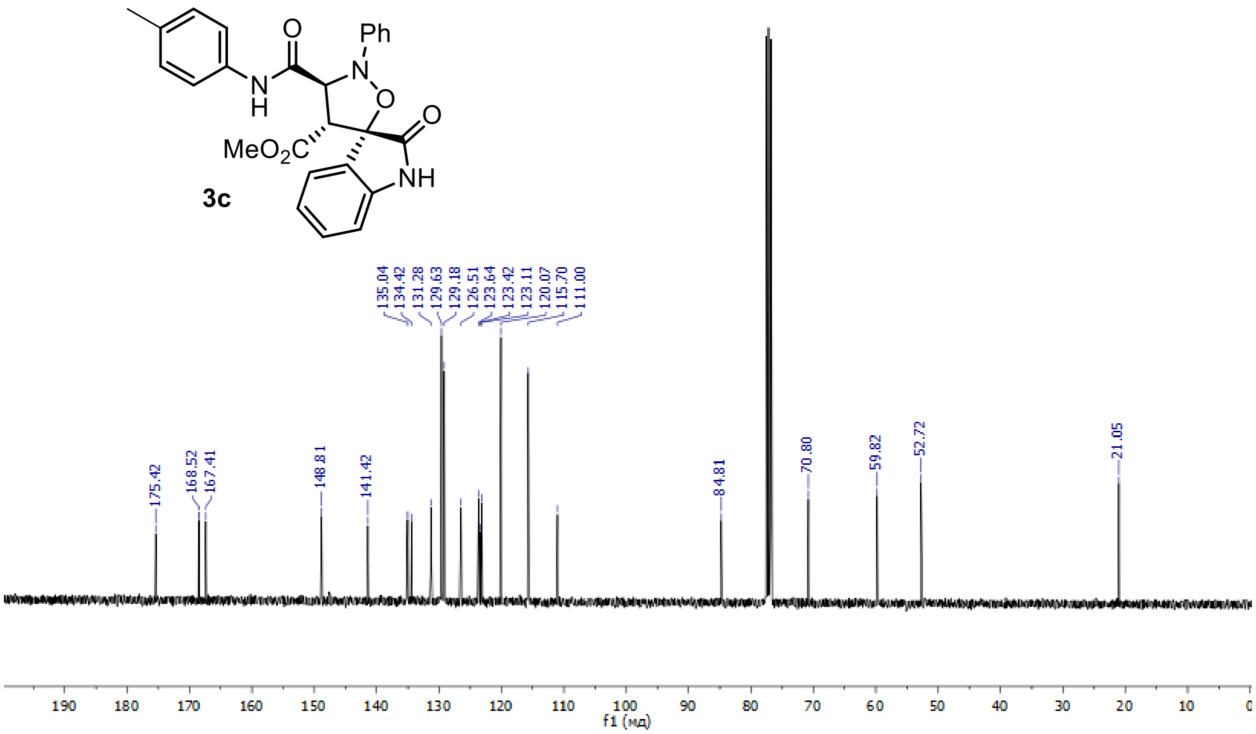

^{1}H NMR (400 MHz, DMSO-d6) spectrum of compound **3'c**

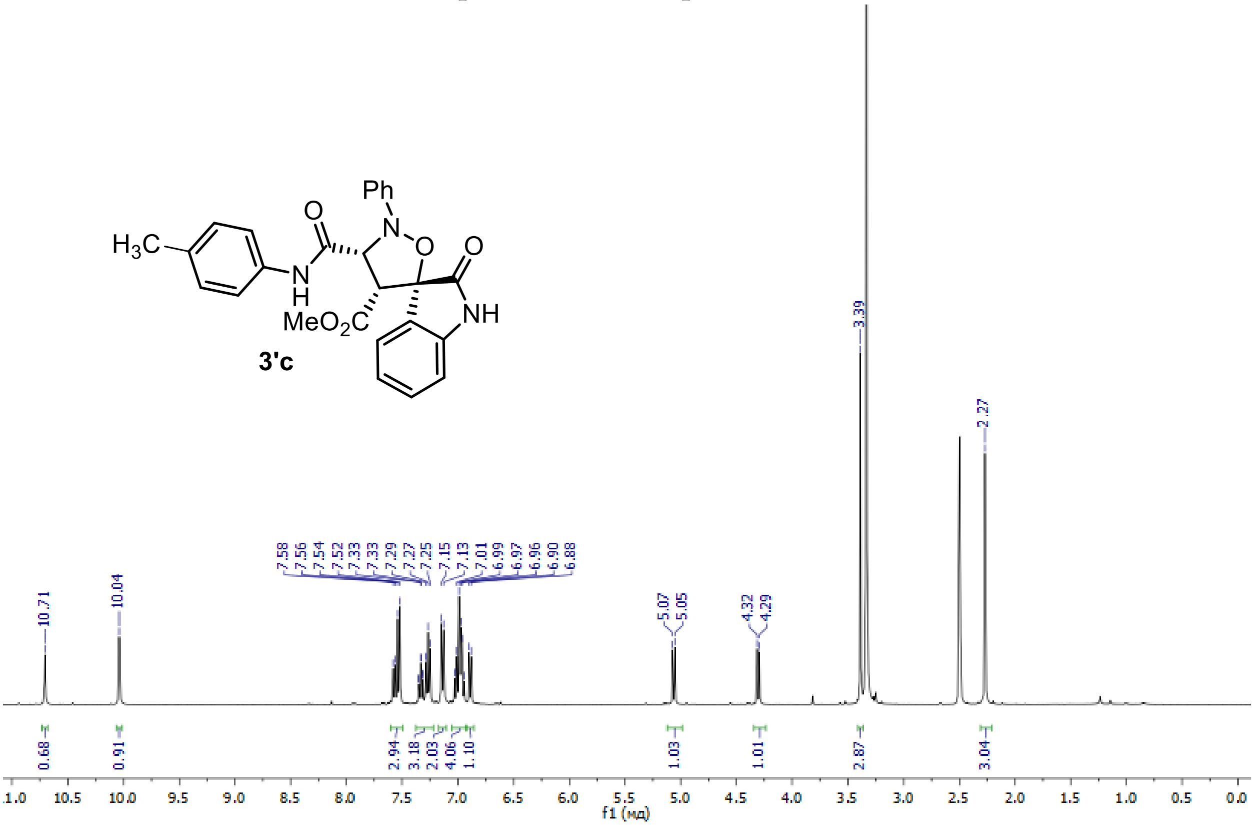

^{13}C NMR (100 MHz, DMSO-d6) spectrum of compound **3'c**

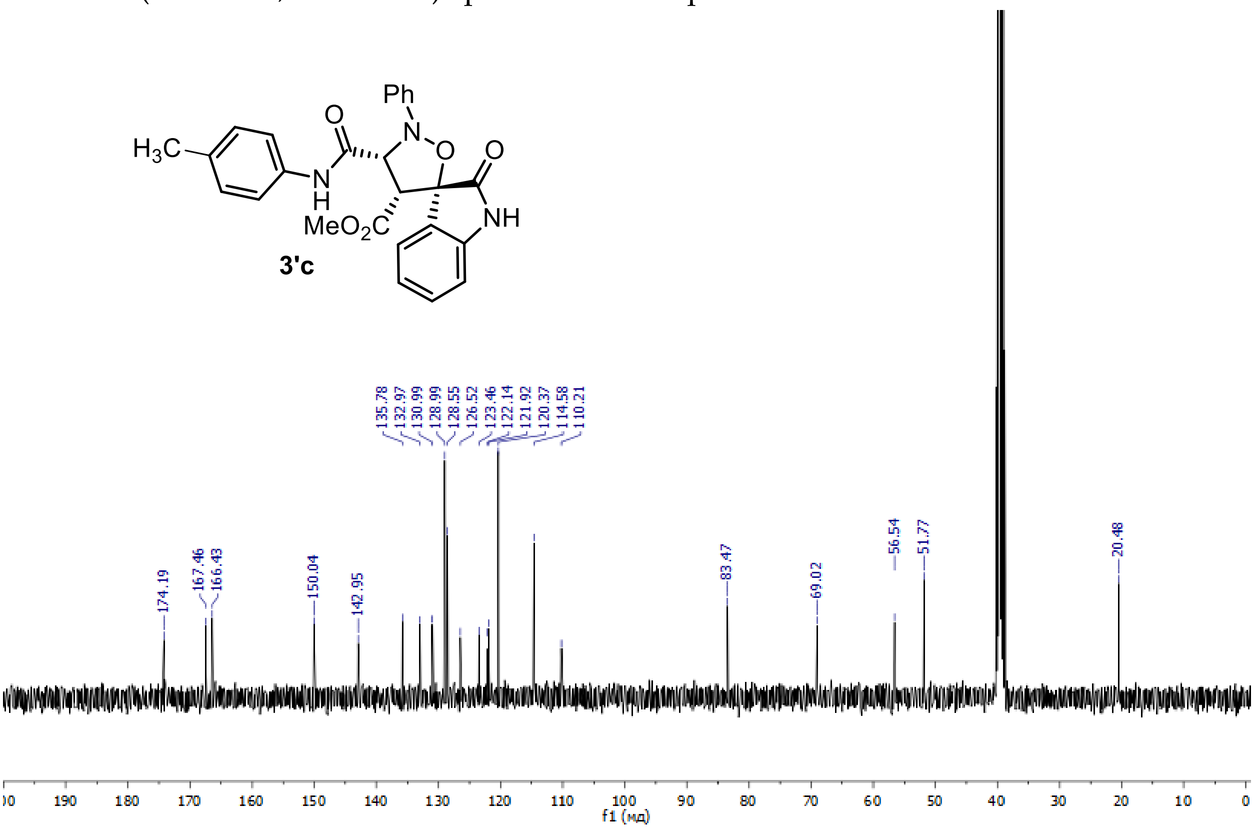

^{1}H NMR (500 MHz, CDCl3) spectrum of the mixture of compounds **3'c** and **4c**

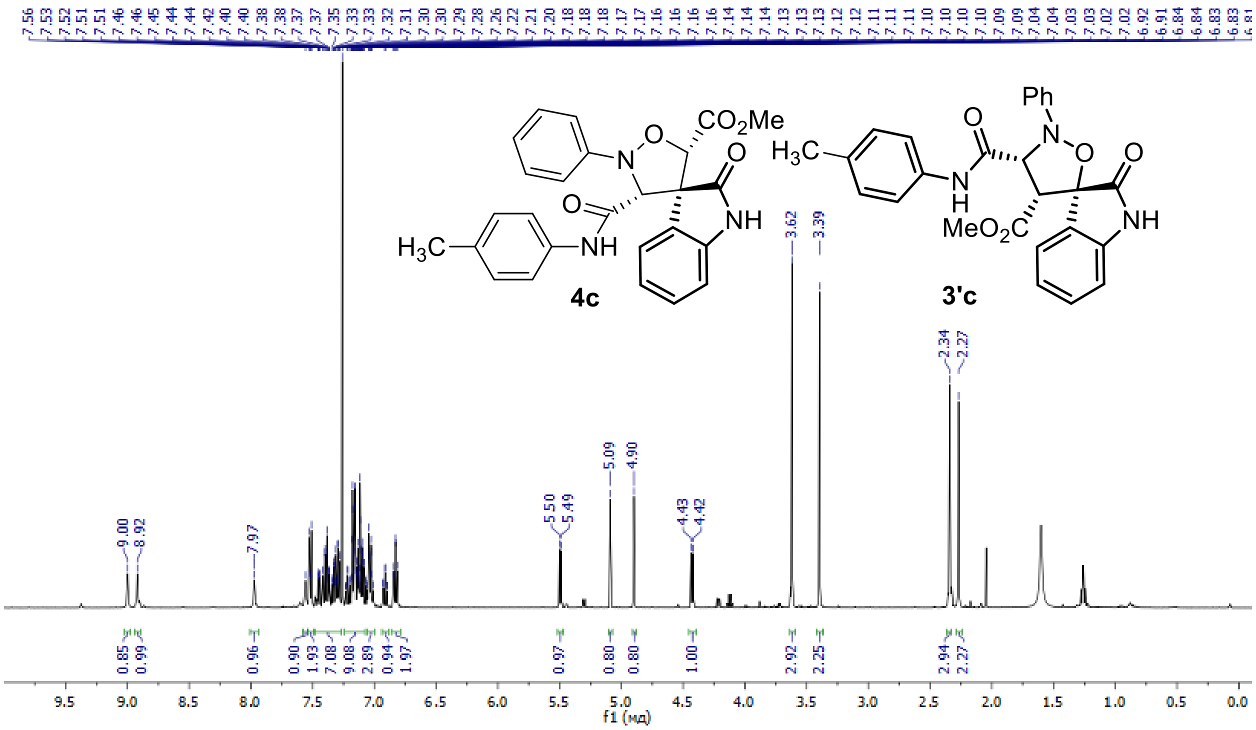

^{13}C NMR (125 MHz, CDCl3) spectrum of the mixture of compounds **3'c** and **4c**

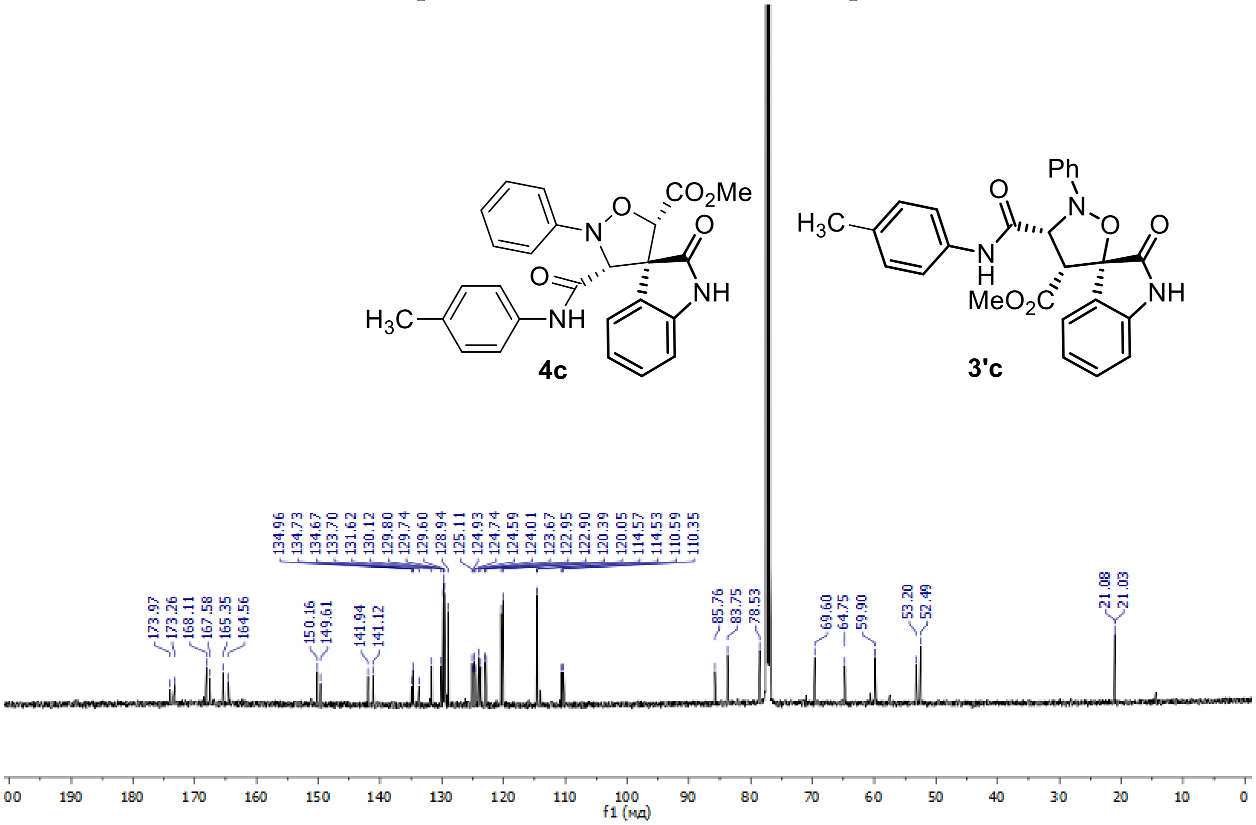

^{1}H NMR (400 MHz, $CDCl_3$) spectrum of compound **3d**

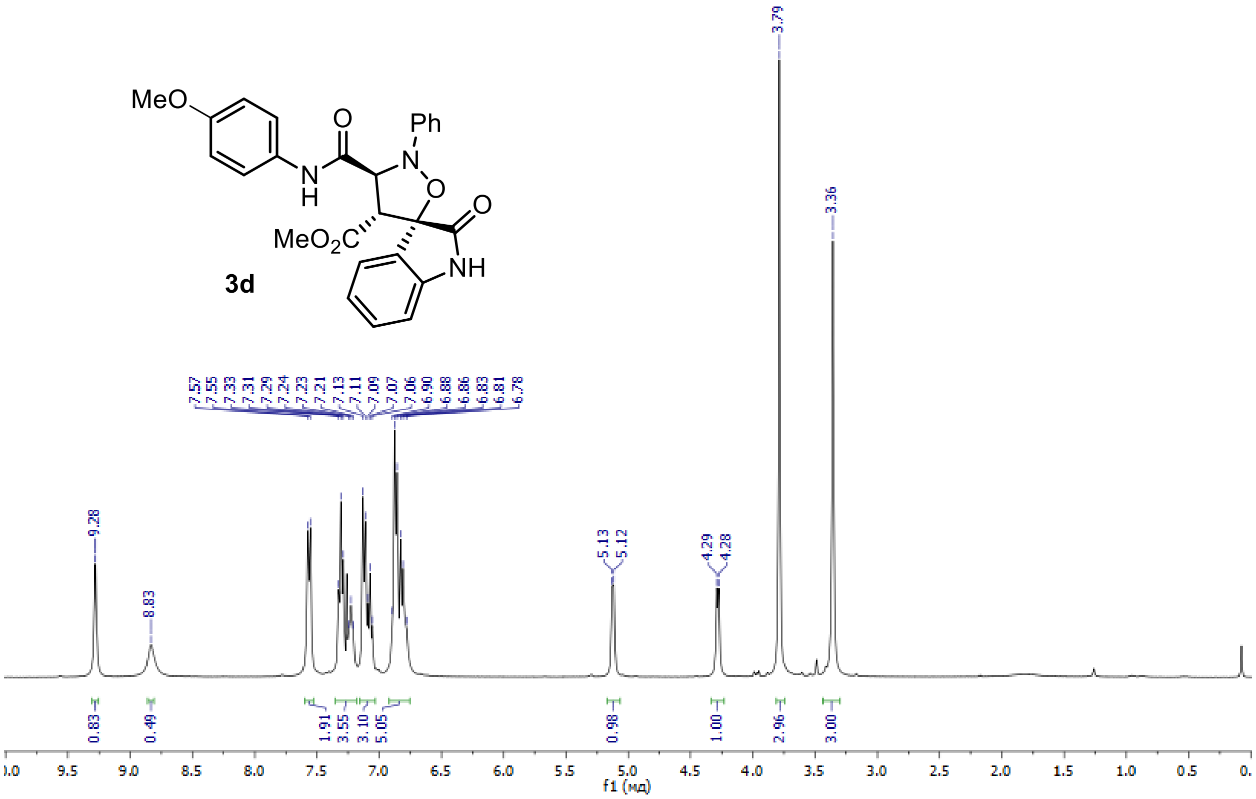

^{13}C NMR (100 MHz, $CDCl_3$) spectrum of compound **3d**

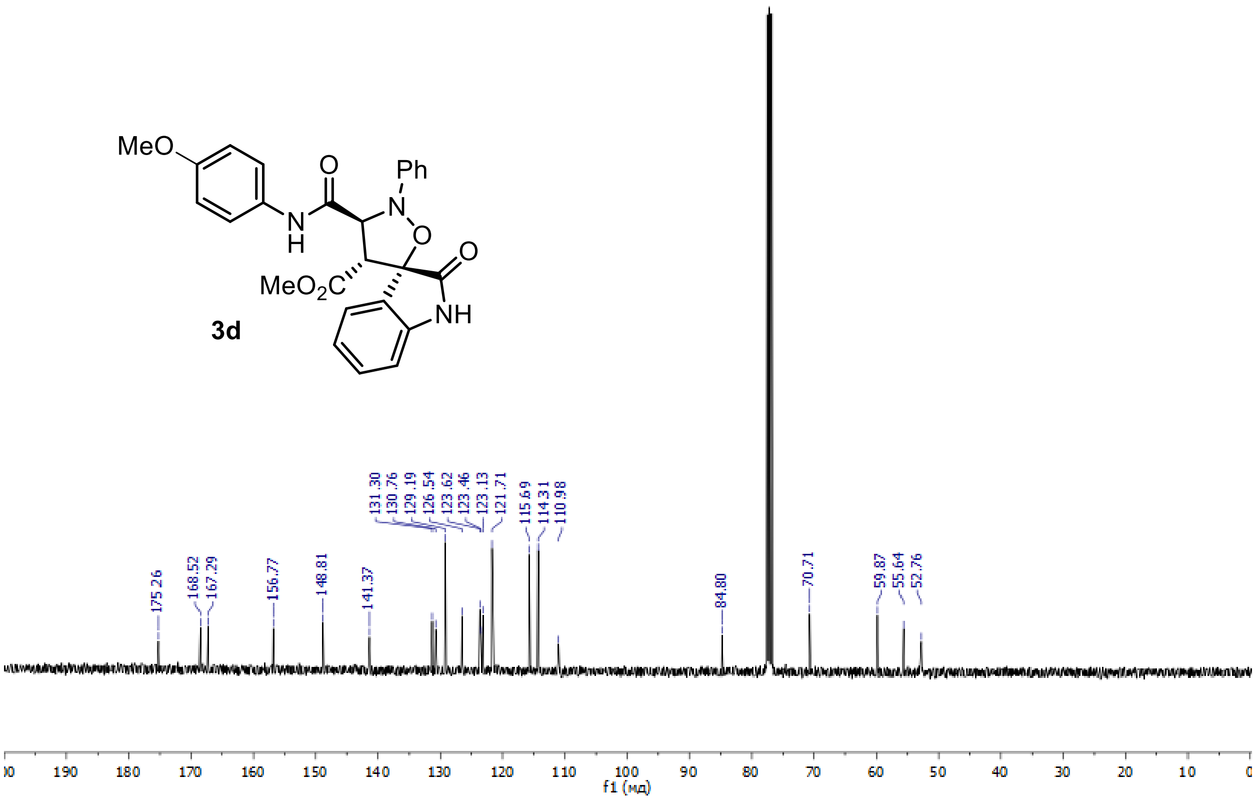

^{1}H NMR (500 MHz, $CDCl_3$) spectrum of compound **3'd**

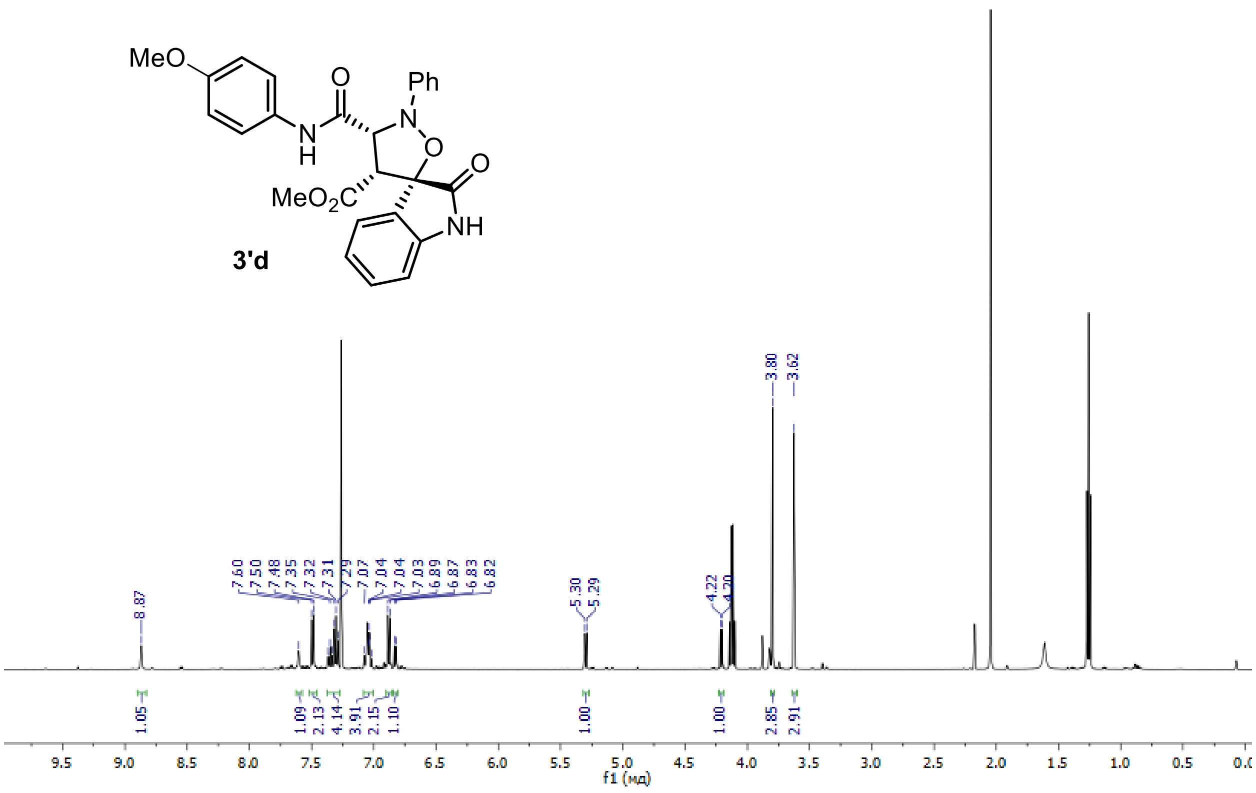

^{13}C NMR (125 MHz, $CDCl_3$) spectrum of compound **3'd**

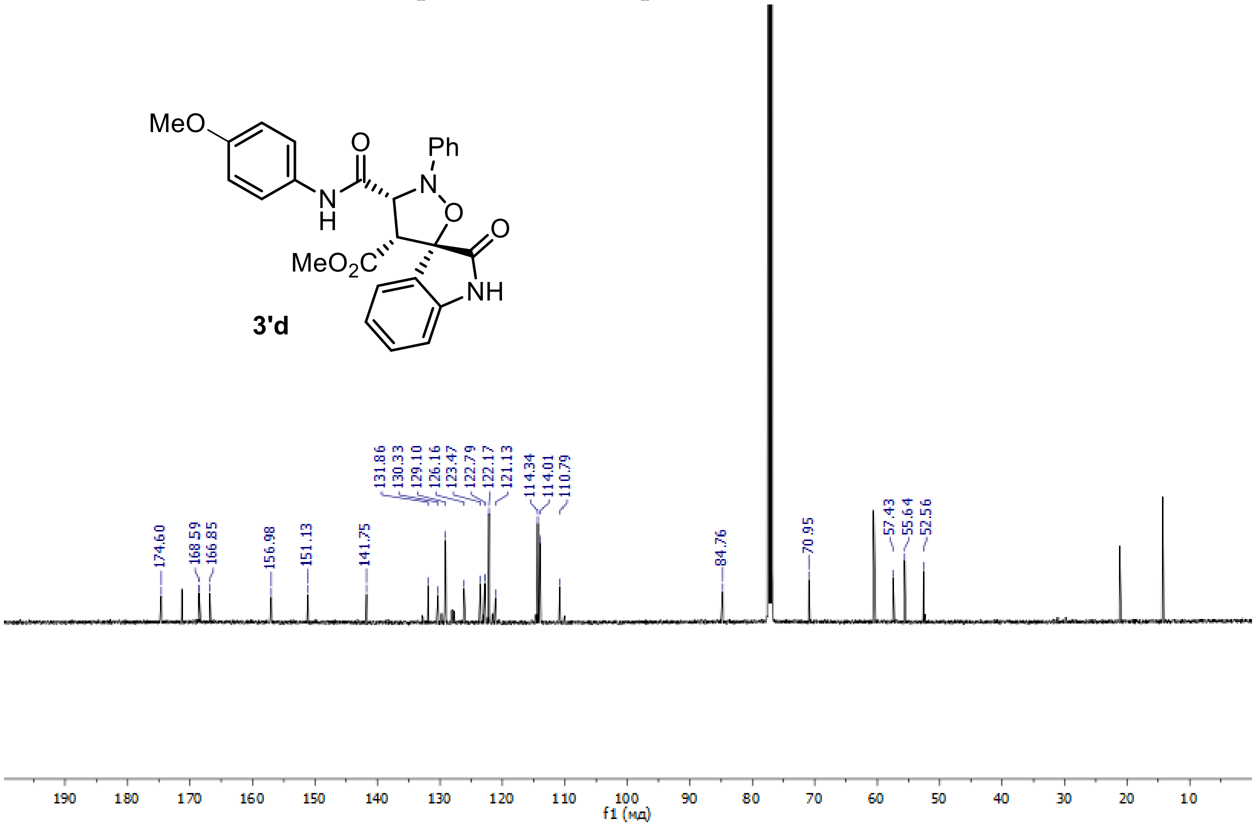

^{1}H NMR (400 MHz, CDCl3) spectrum of the mixture of compounds **3'd** and **4d**

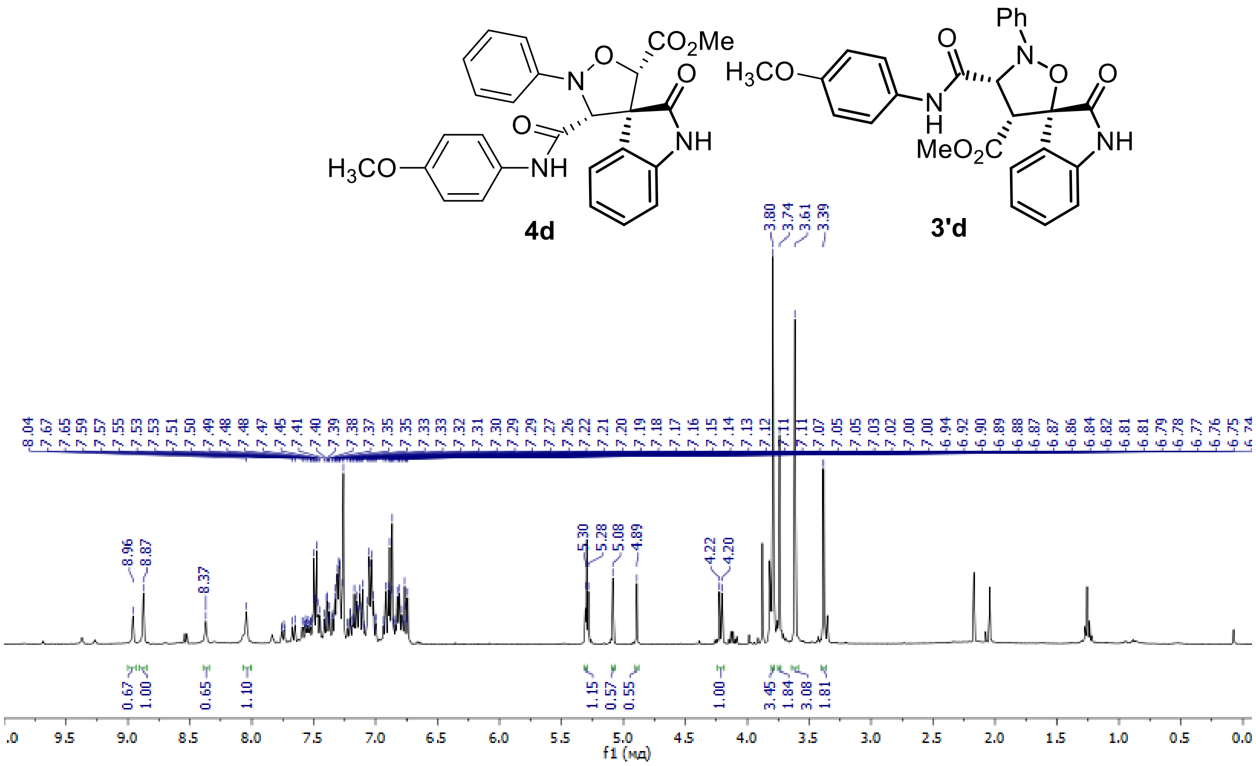

^{1}H NMR (400 MHz, $CDCl_3$) spectrum of compound **3e**

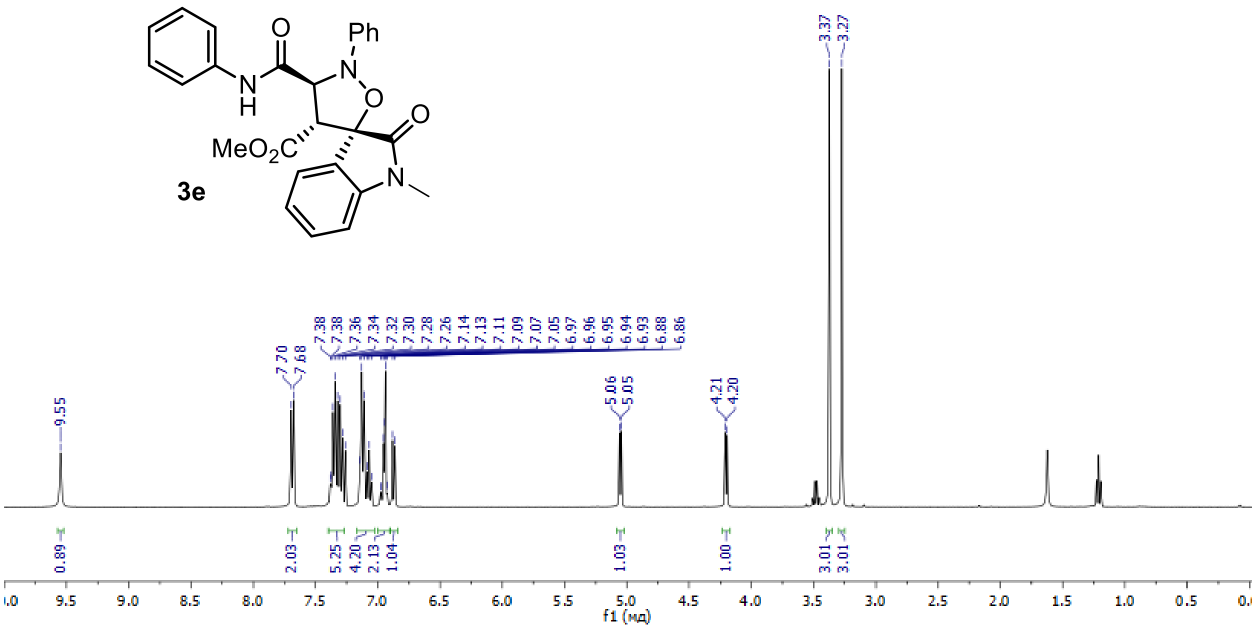

^{13}C NMR (100 MHz, $CDCl_3$) spectrum of compound **3e**

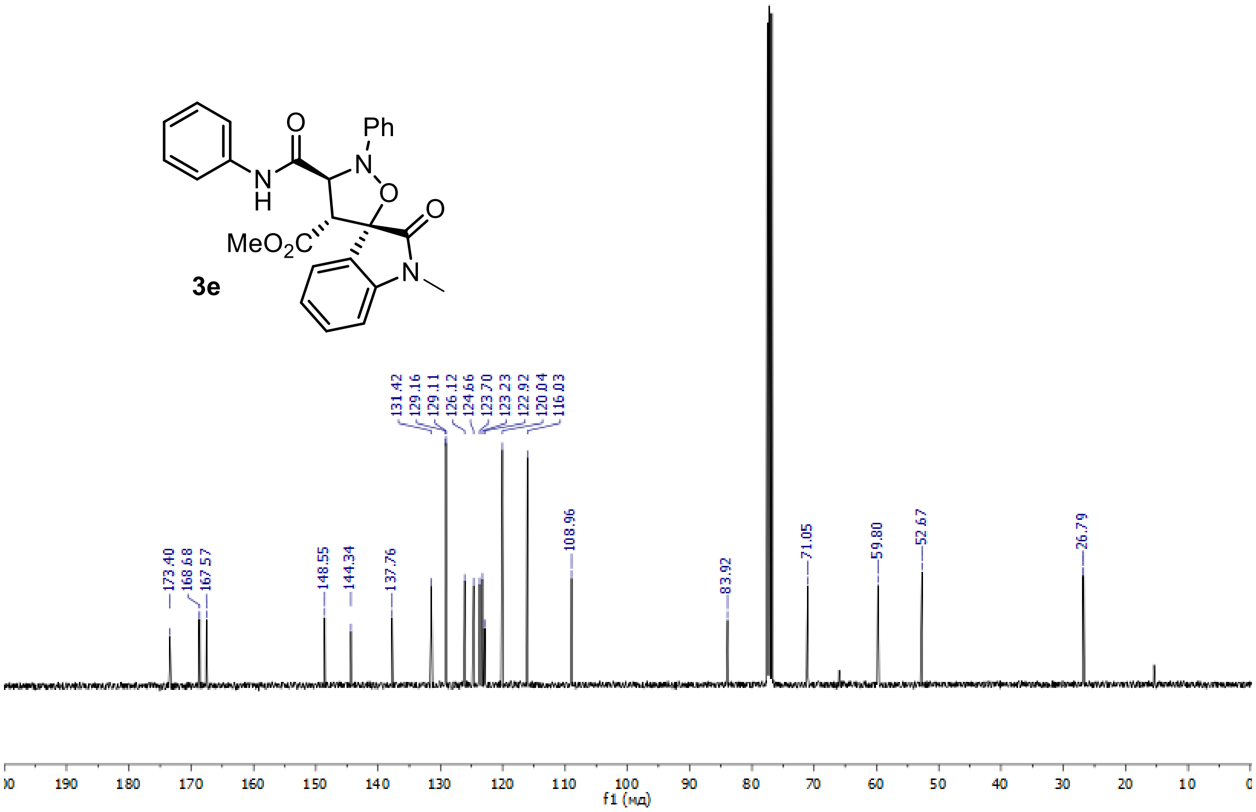

^{1}H NMR (400 MHz, $CDCl_3$) spectrum of compound **3'e**

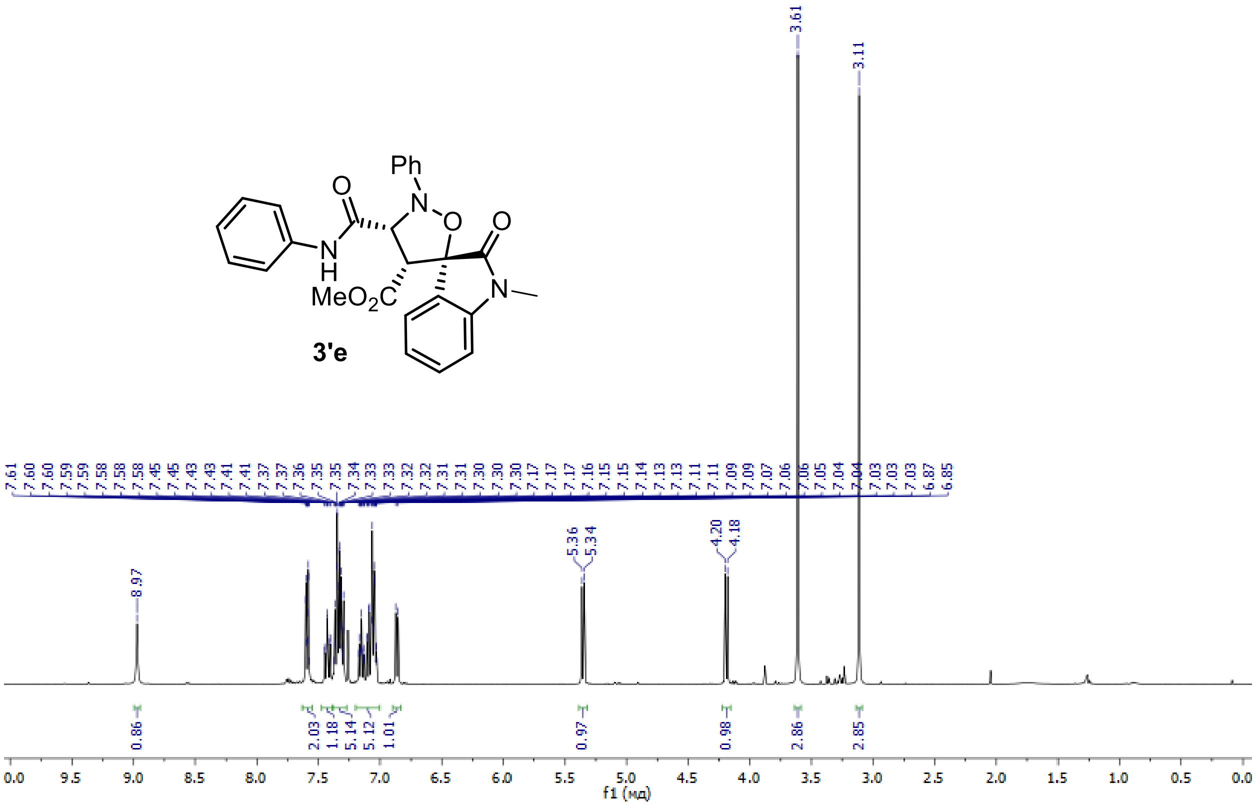

^{13}C NMR (100 MHz, $CDCl_3$) spectrum of compound **3'e**

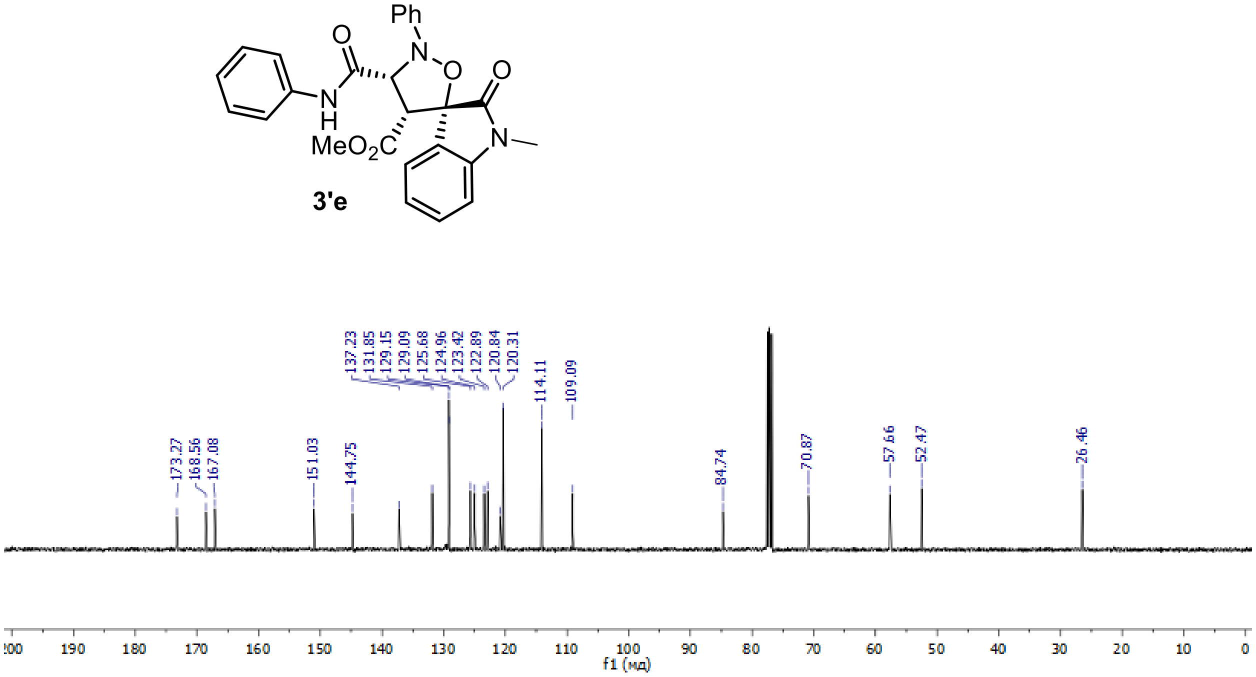

^{1}H NMR (400 MHz, $CDCl_3$) spectrum of the mixture of compounds **3'e** and **4e**

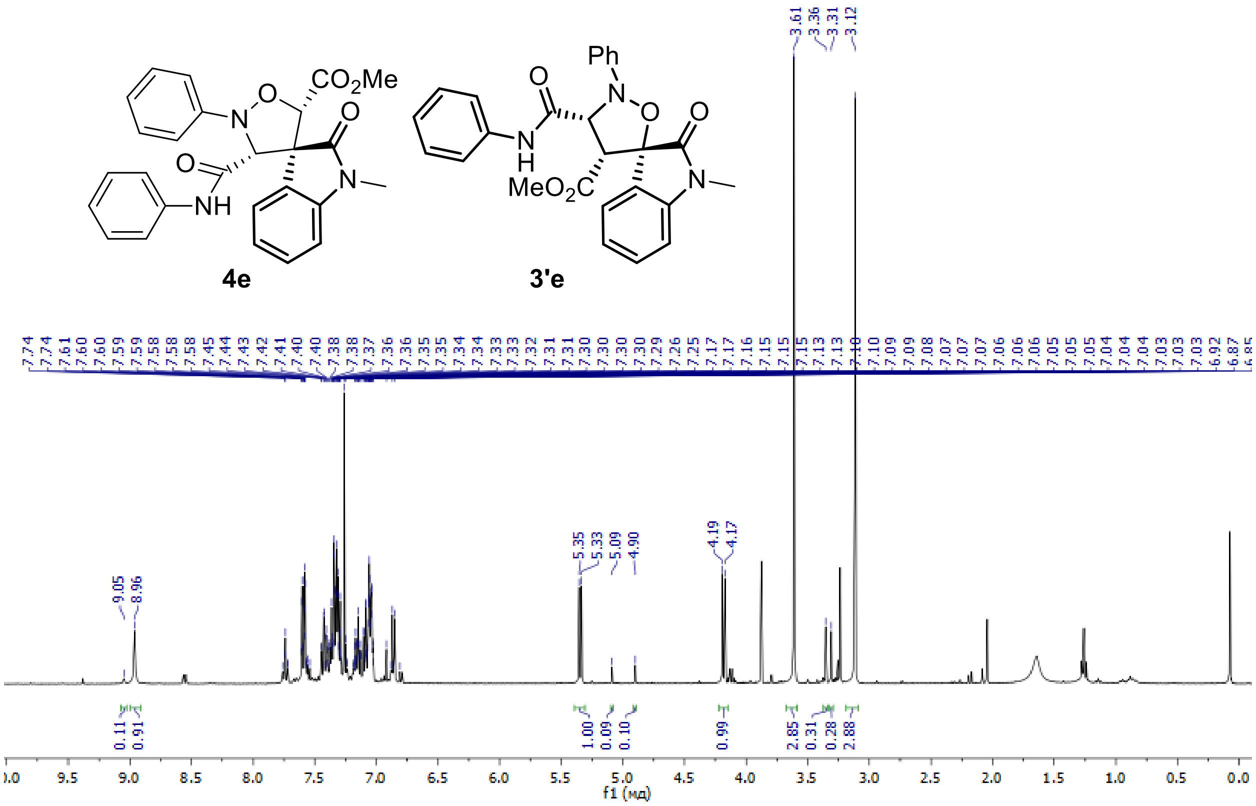

^{1}H NMR (400 MHz, CDCl3) spectrum of compound **3f**

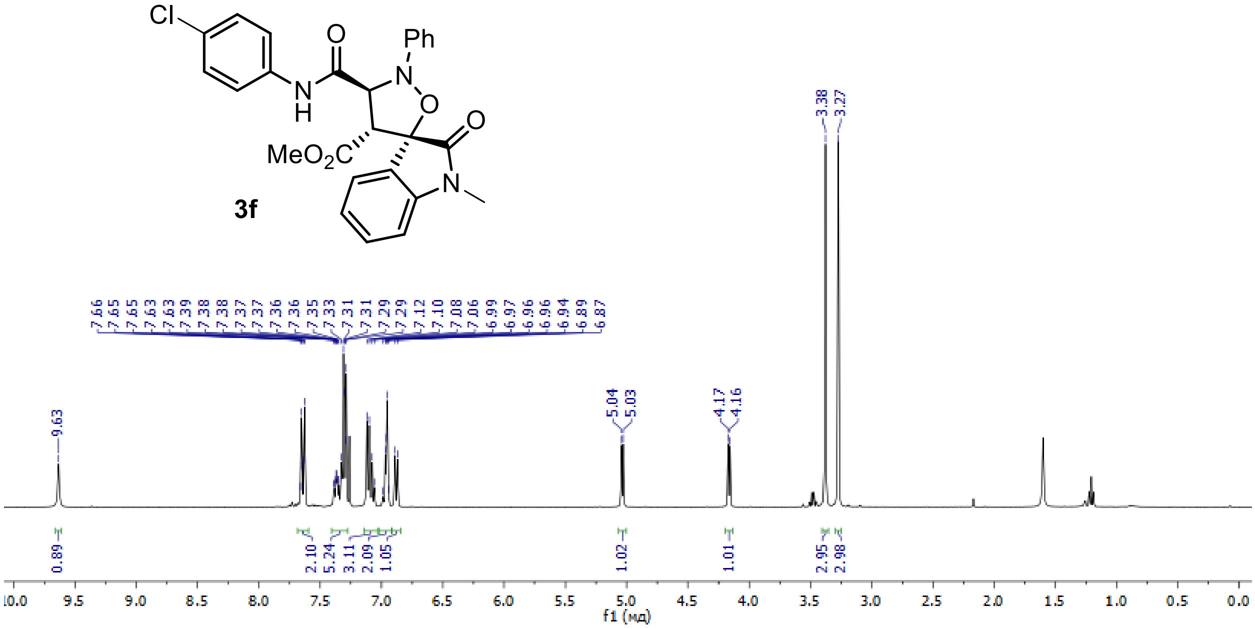

^{13}C NMR (100 MHz, CDCl3) spectrum of compound **3f**

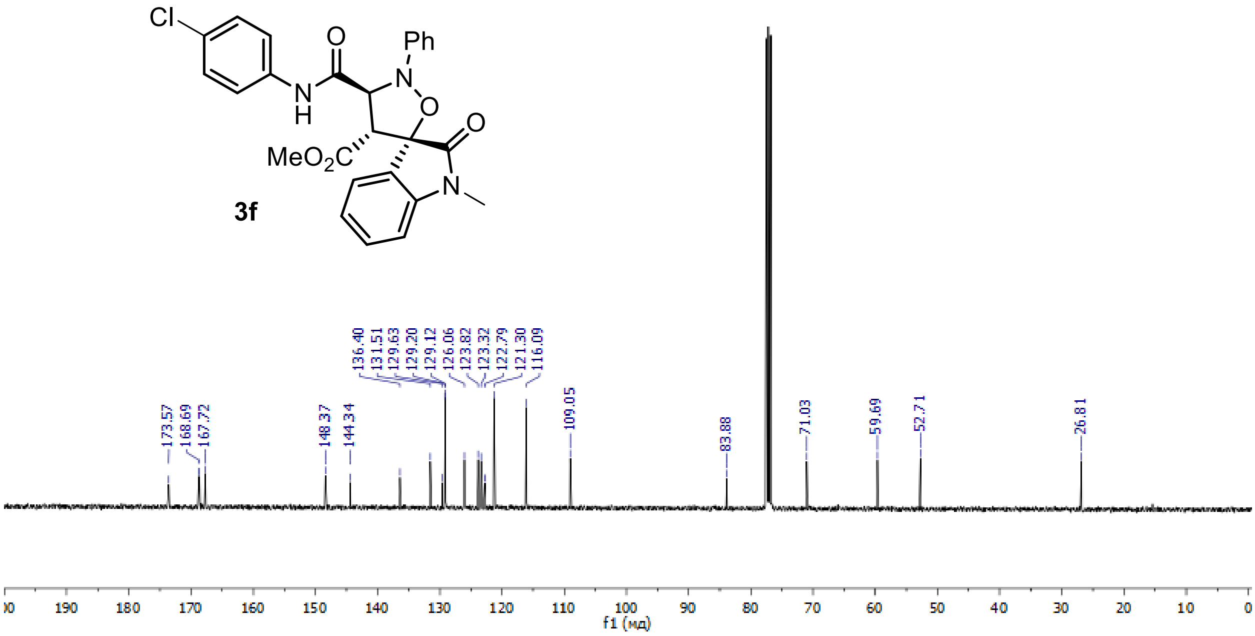

^{1}H NMR (400 MHz, $CDCl_3$) spectrum of compound **3'f**

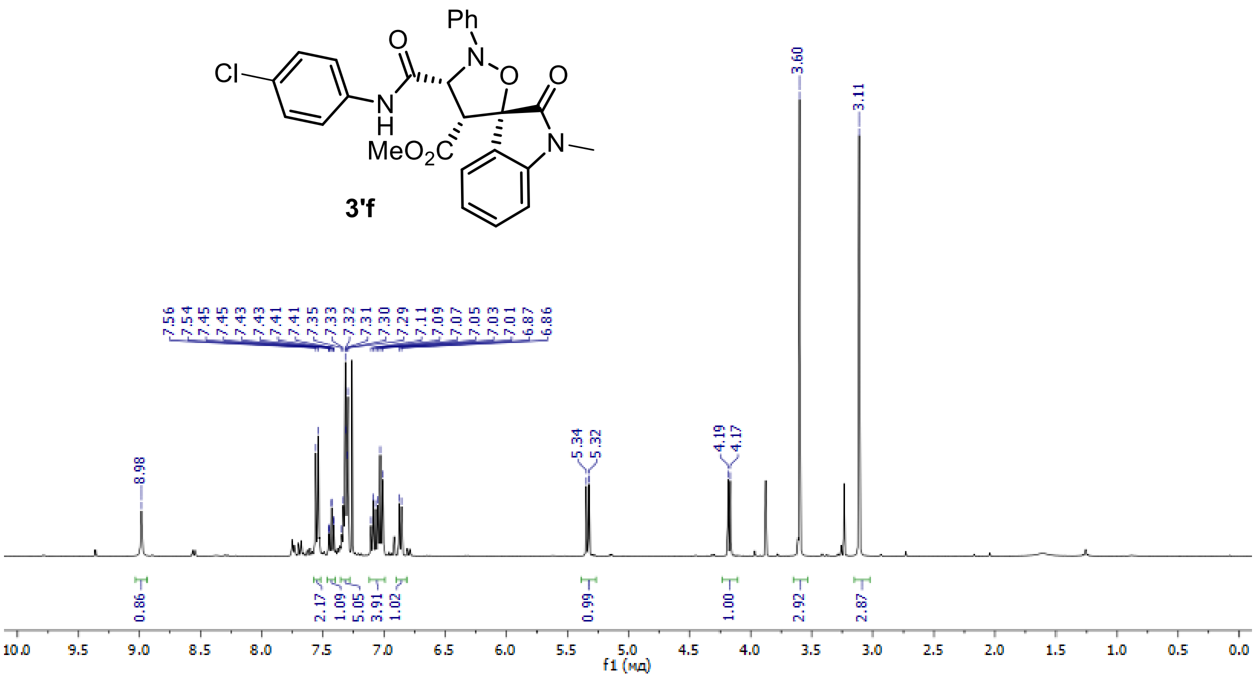

^{13}C NMR (100 MHz, $CDCl_3$) spectrum of compound **3'f**

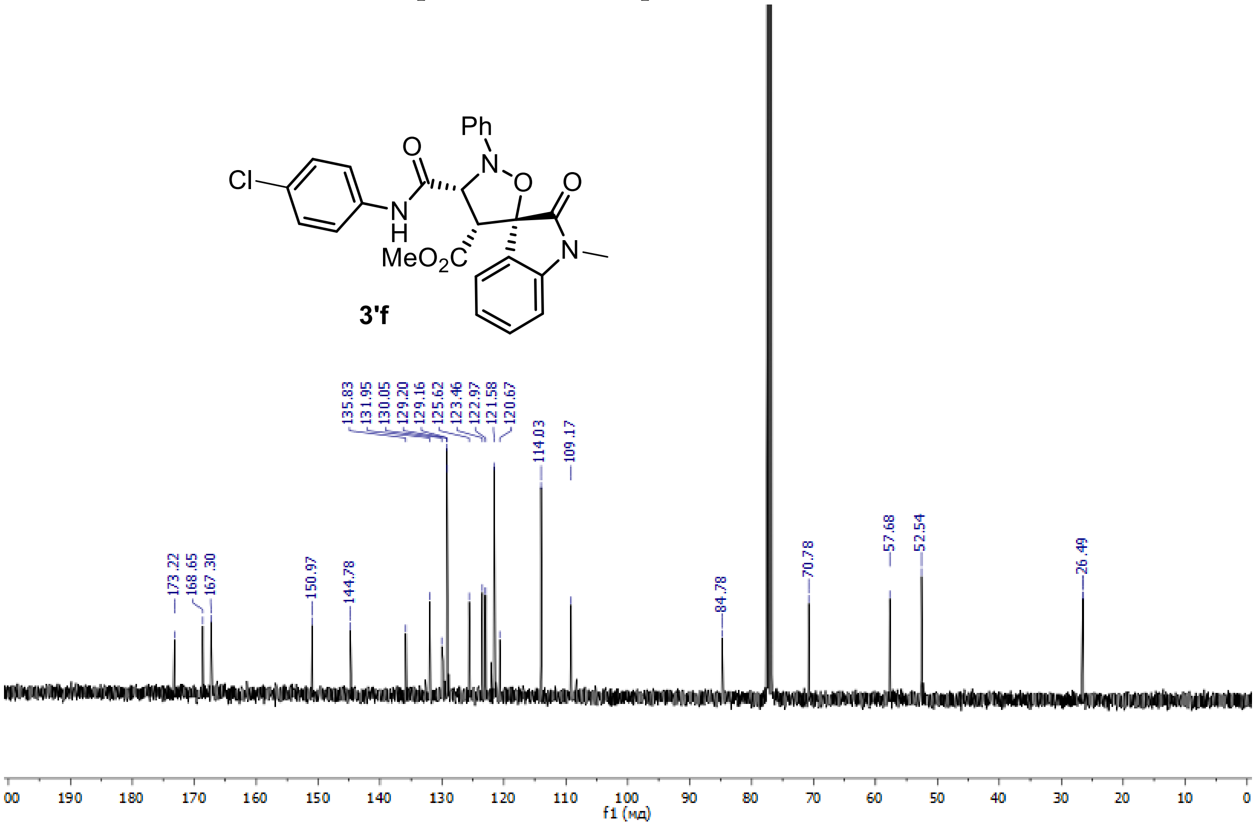

^{1}H NMR (400 MHz, CDCl3) spectrum of the mixture of compounds **3'f** and **4f**

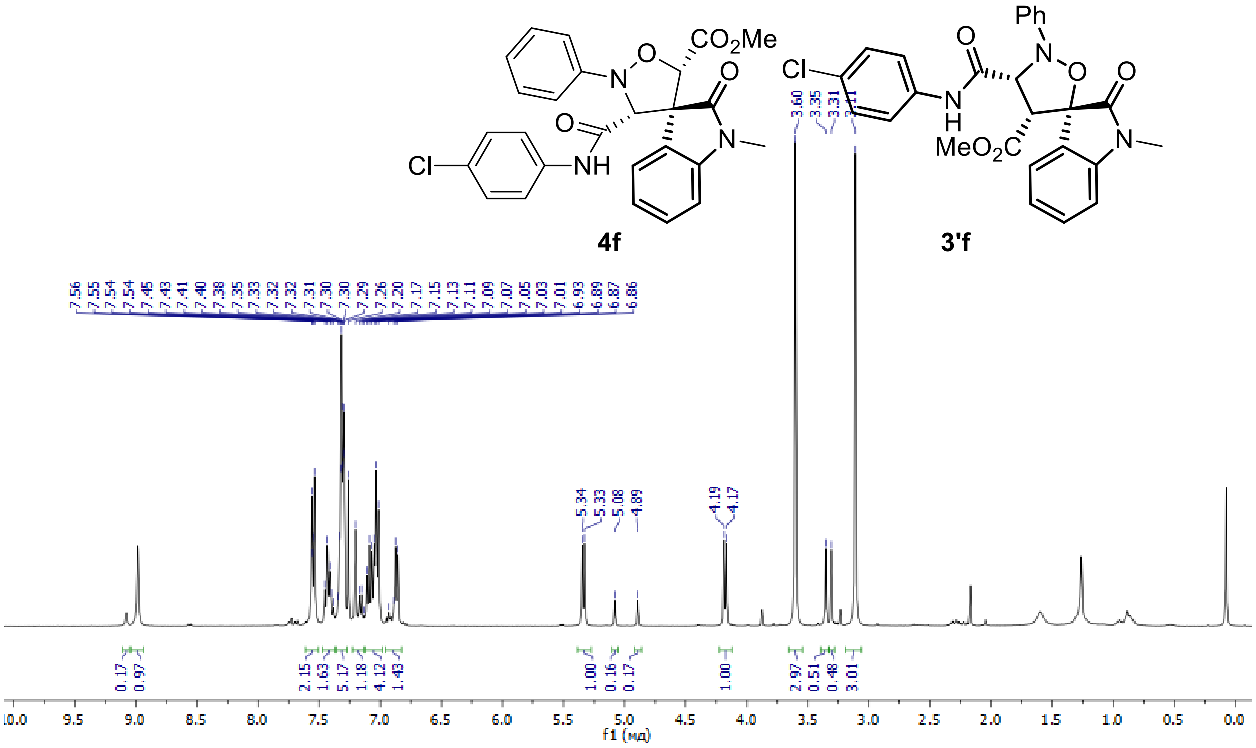

^{1}H NMR (400 MHz, $CDCl_3$) spectrum of compound **3g**

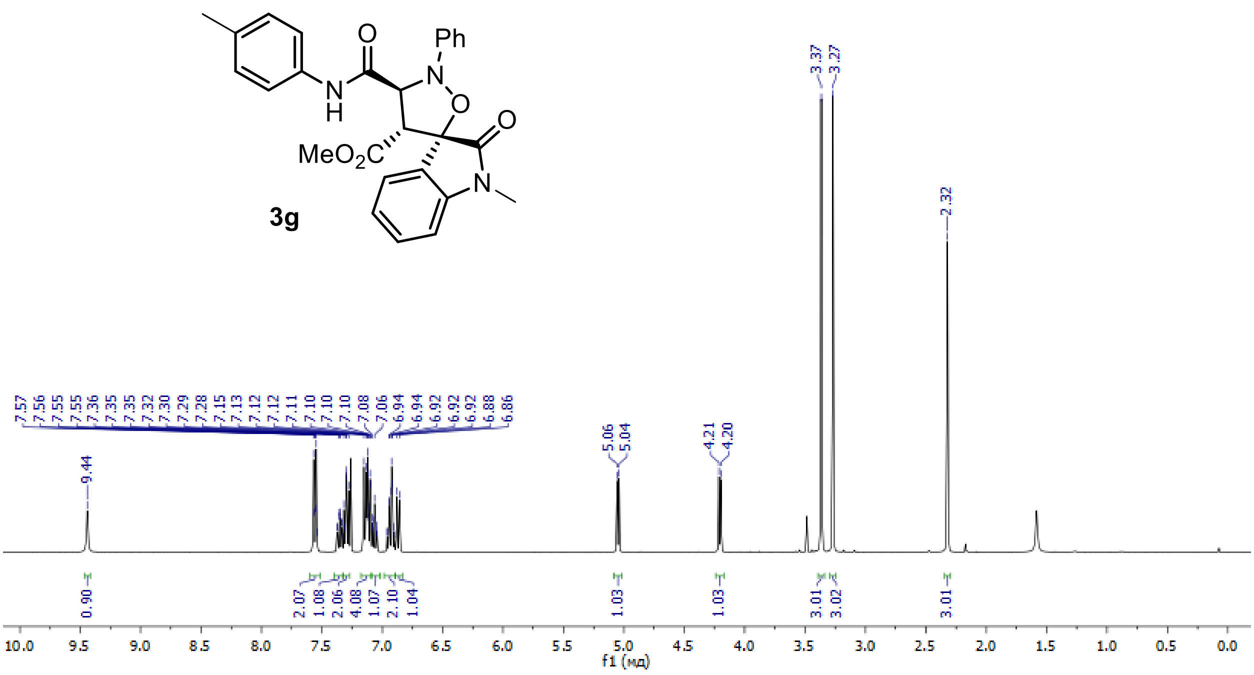

^{13}C NMR (100 MHz, $CDCl_3$) spectrum of compound **3g**

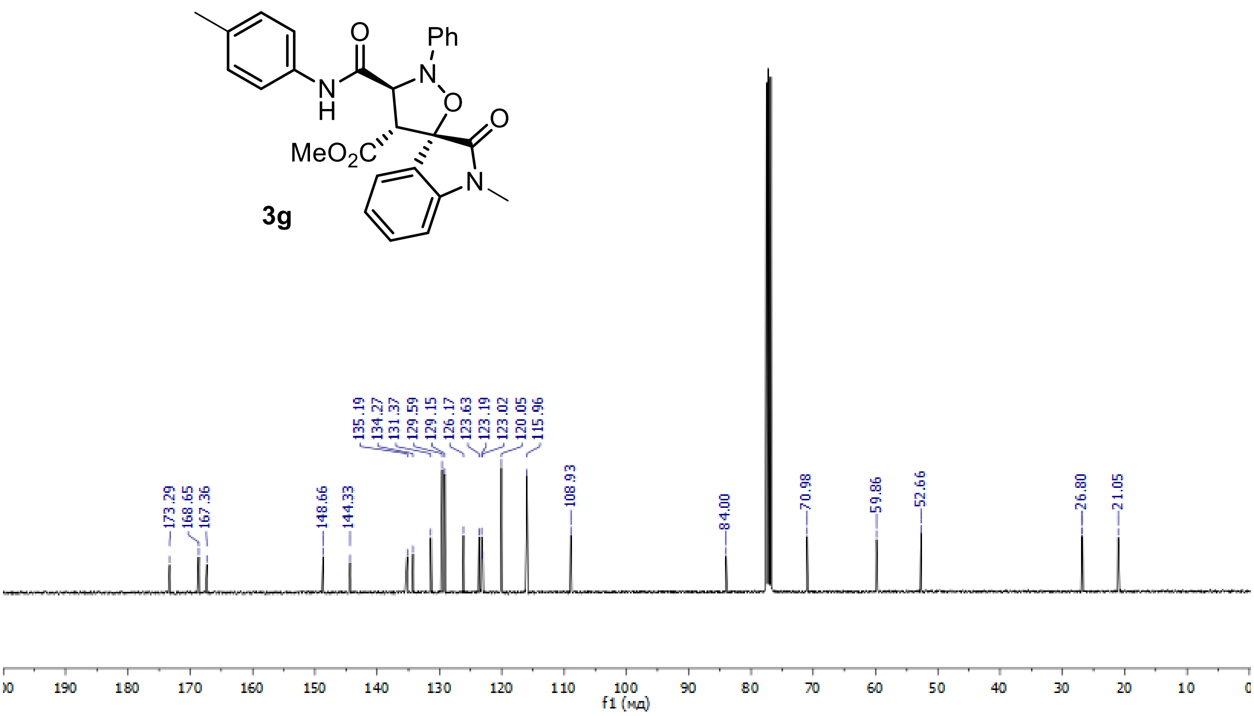

^{1}H NMR (400 MHz, $CDCl_3$) spectrum of the mixture of compounds **3'g** and **4g**

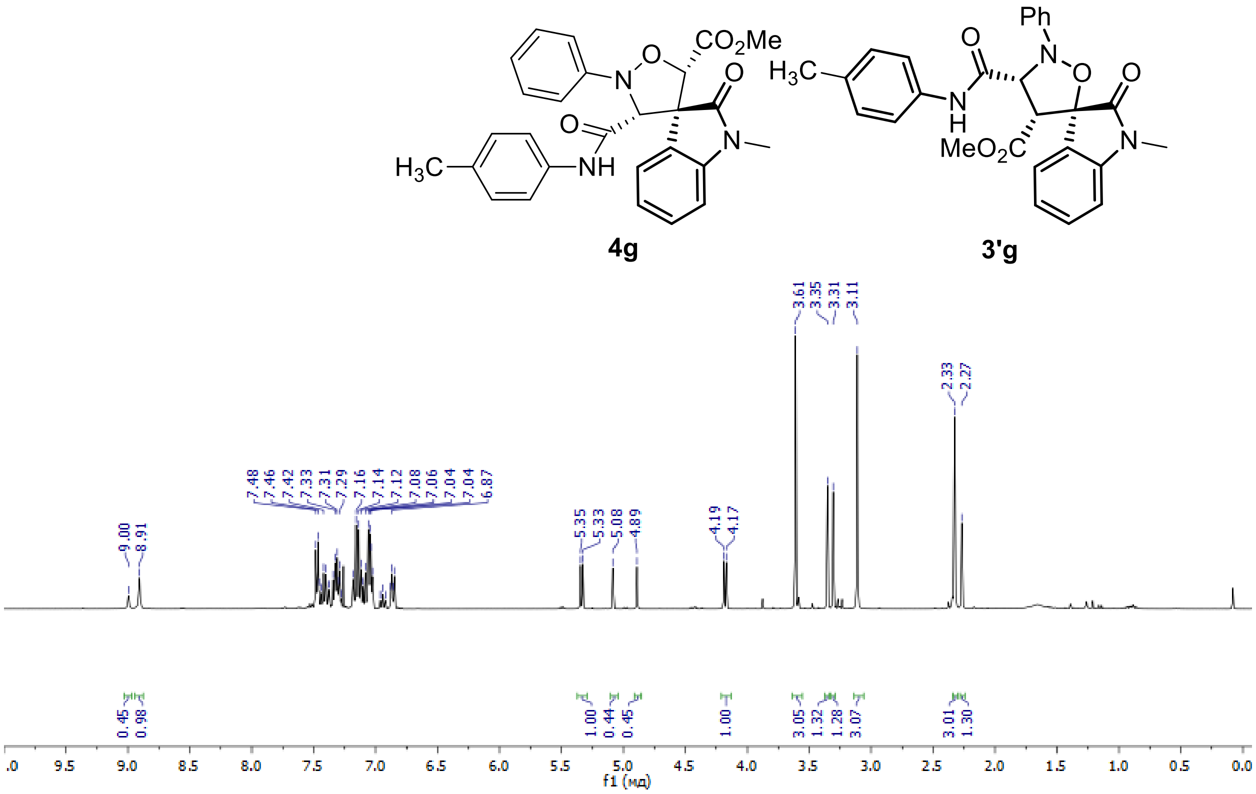

^{13}C NMR (100 MHz, $CDCl_3$) spectrum of the mixture of compounds **3'g** and **4g**

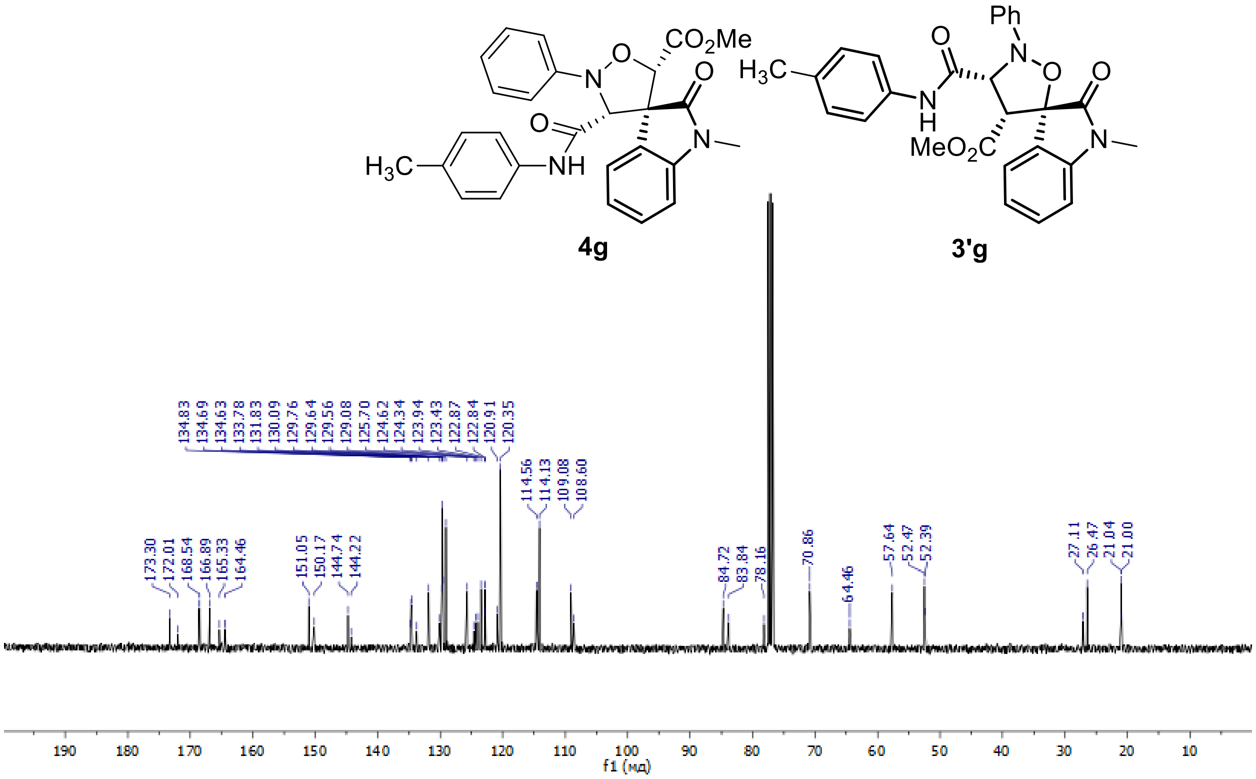

^{1}H NMR (400 MHz, $CDCl_3$) spectrum of compound **3h**

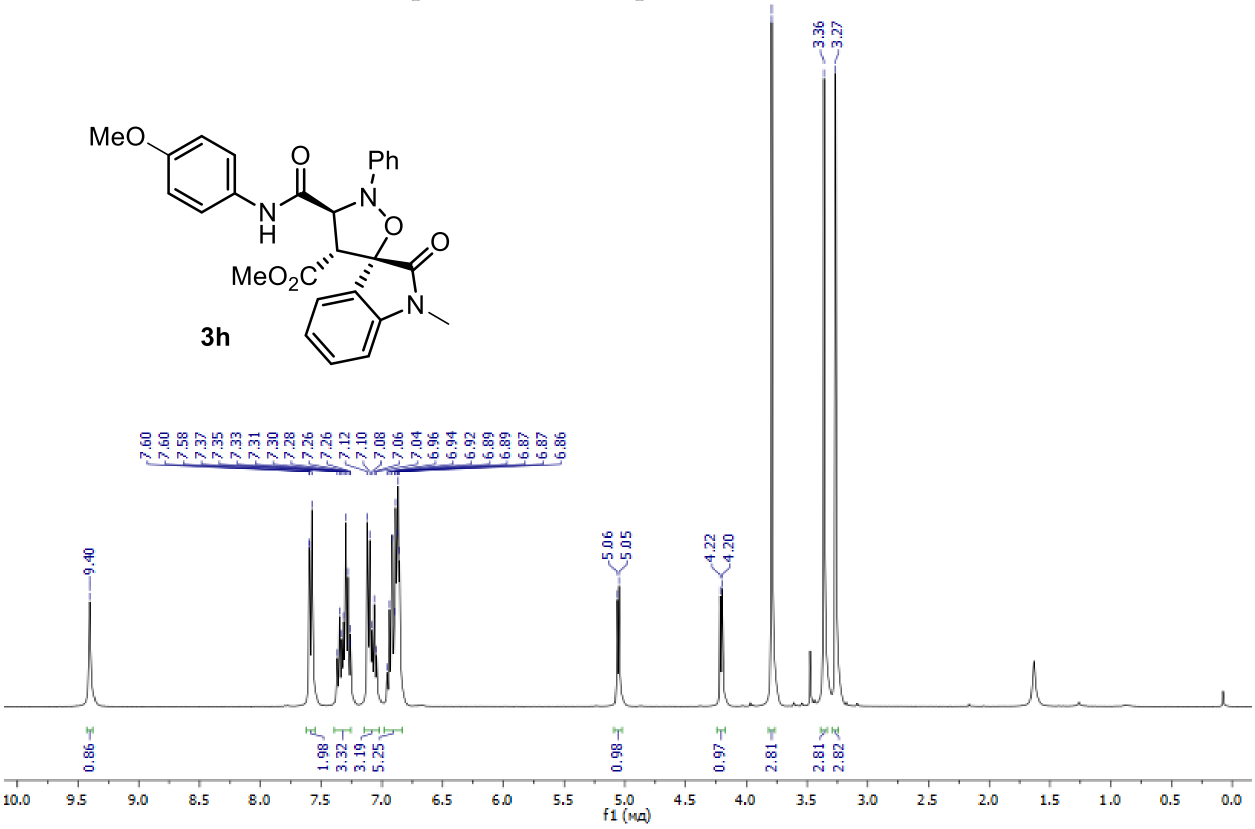

^{13}C NMR (100 MHz, $CDCl_3$) spectrum of compound **3h**

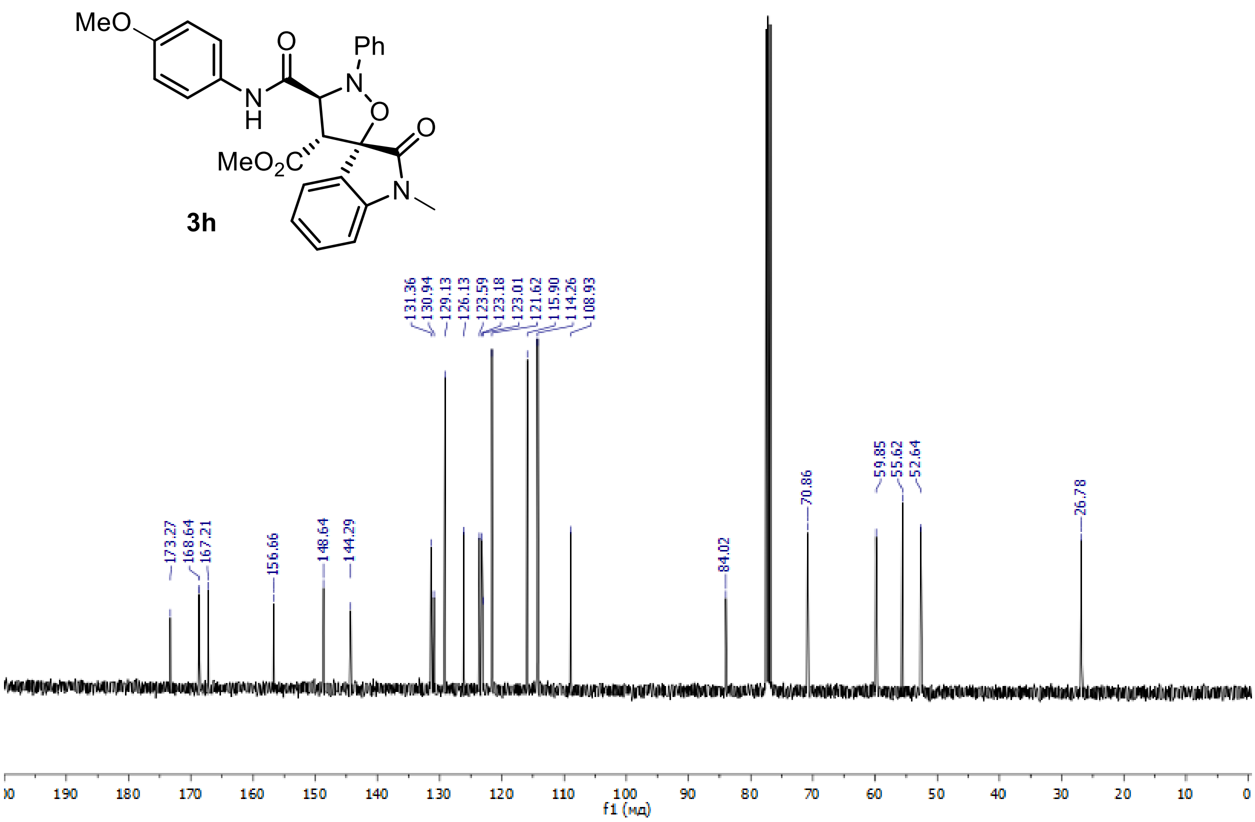

^{1}H NMR (400 MHz, $CDCl_3$) spectrum of compound **3'h**

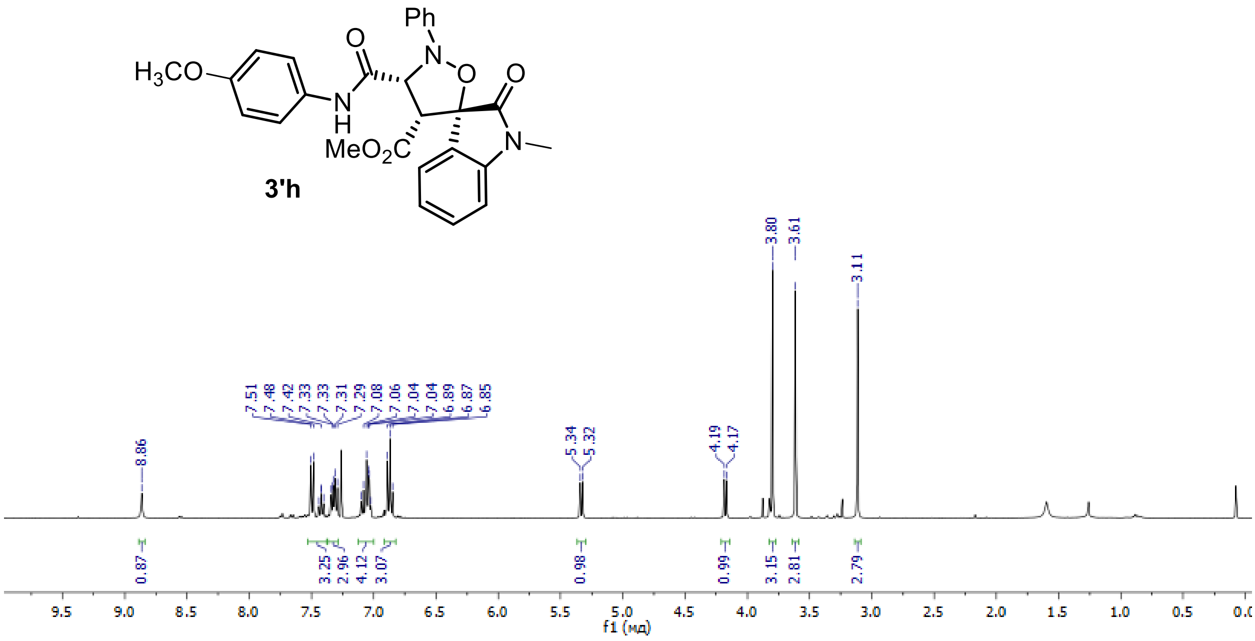

^{13}C NMR (100 MHz, $CDCl_3$) spectrum of compound **3'h**

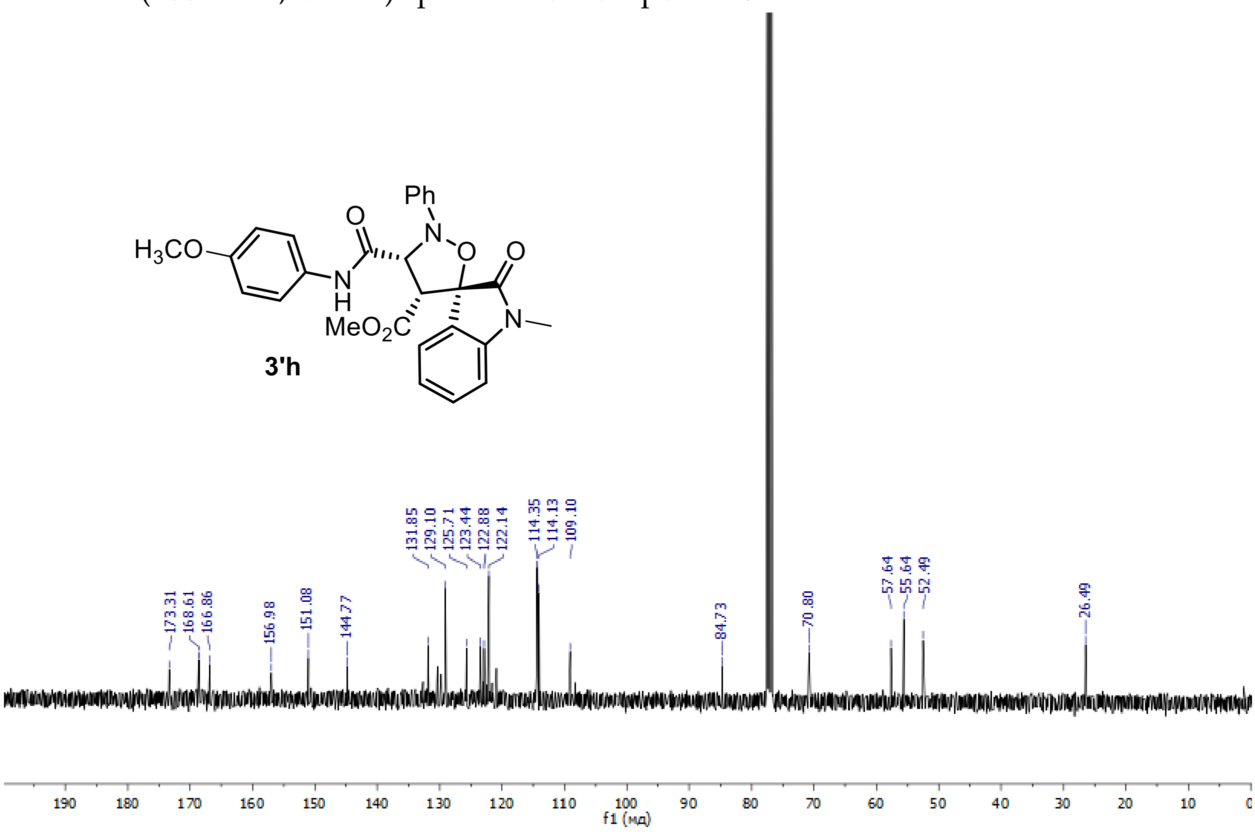

^{1}H NMR (400 MHz, $CDCl_3$) spectrum of the mixture of compounds **3'h** and **4h**

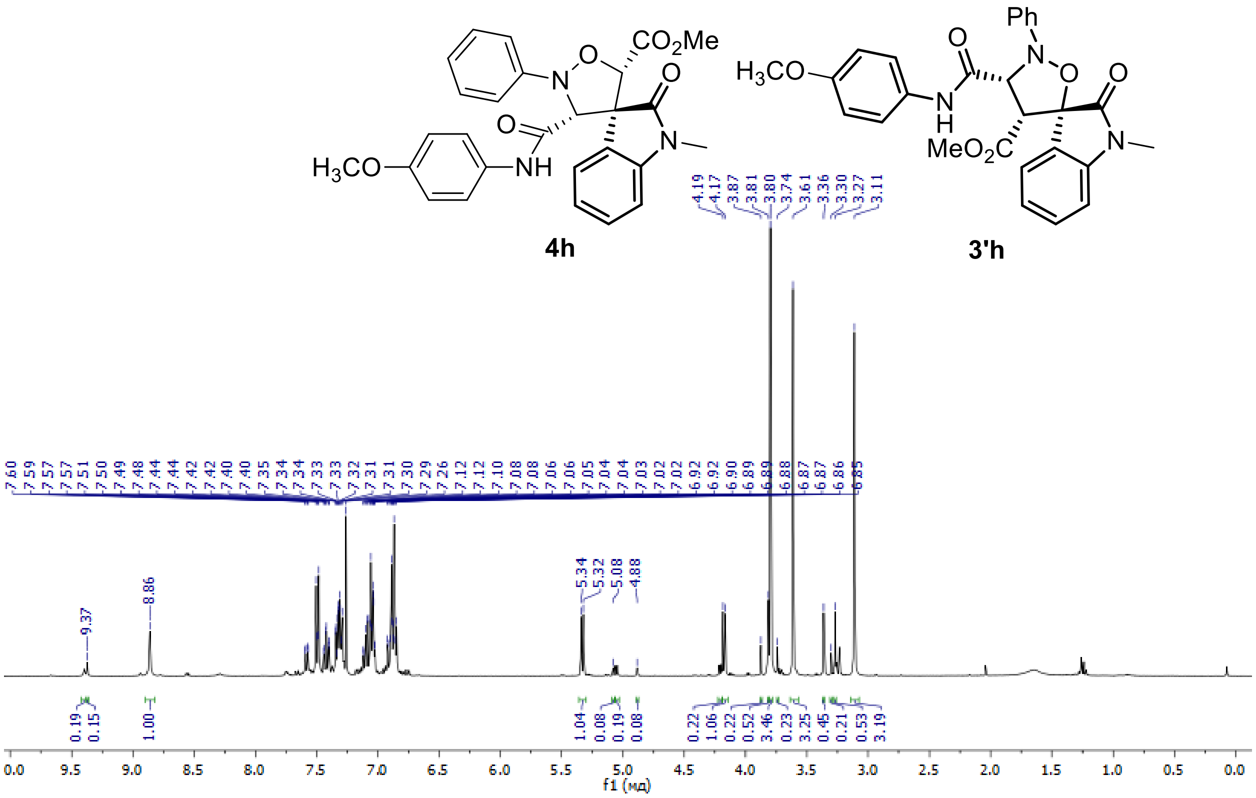

^{13}C NMR (100 MHz, $CDCl_3$) spectrum of the mixture of compounds **3'h** and **4h**

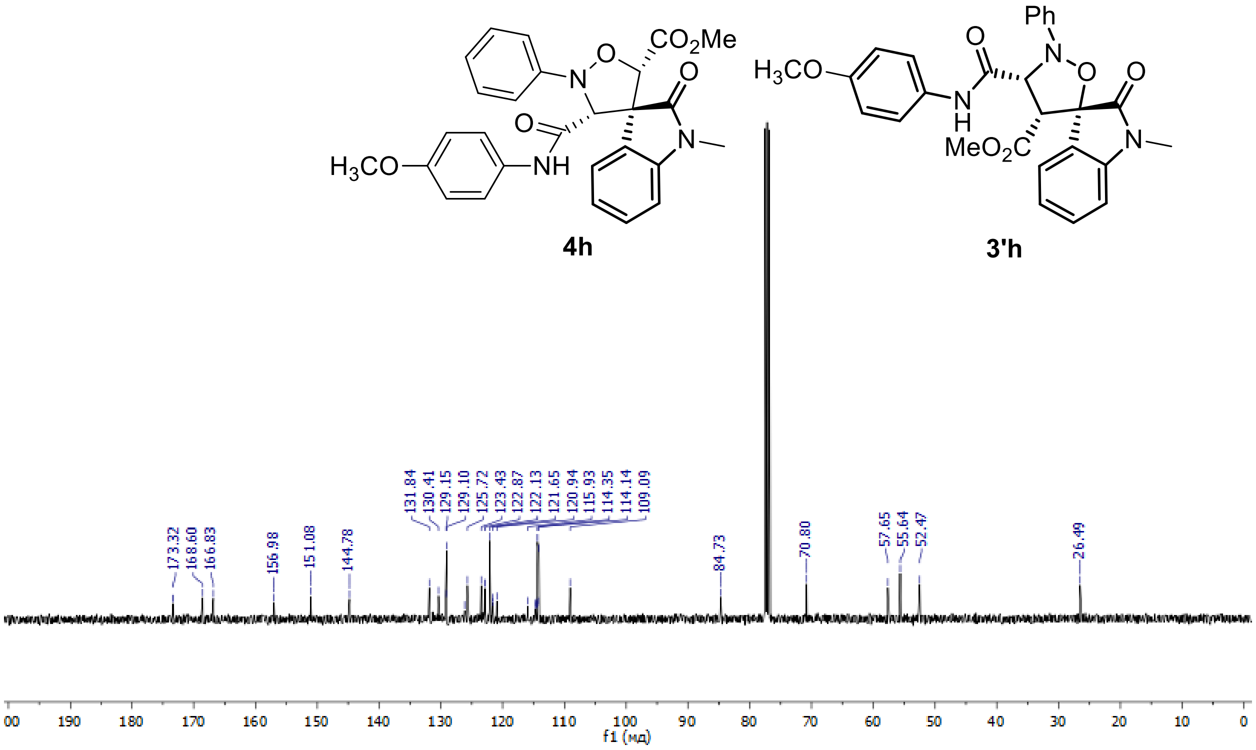

^{1}H NMR (400 MHz, $CDCl_3$) spectrum of compound **6a**

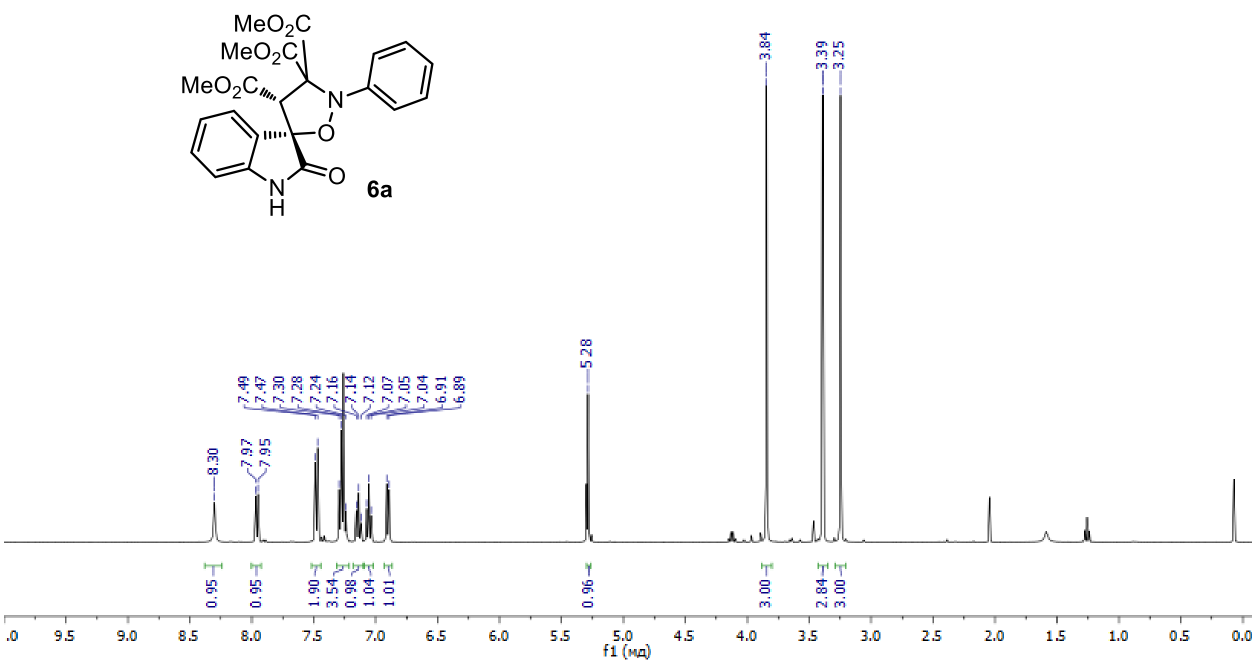

^{13}C NMR (100 MHz, $CDCl_3$) spectrum of compound **6a**

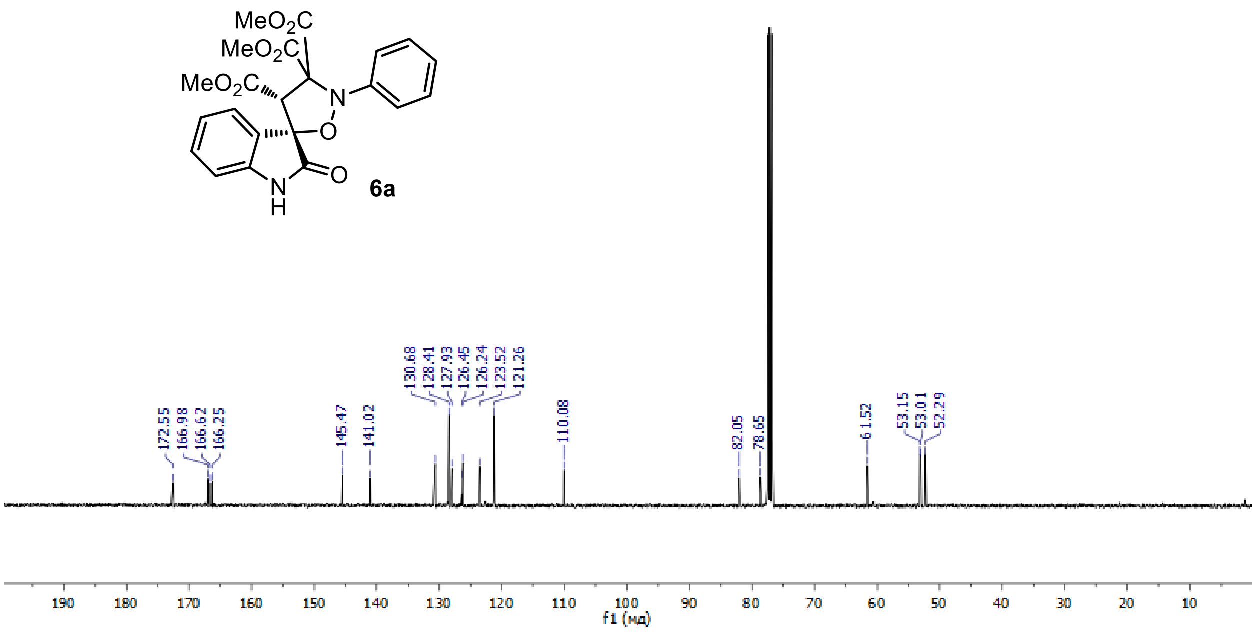

^{1}H NMR (400 MHz, $CDCl_3$) spectrum of compound **6b**

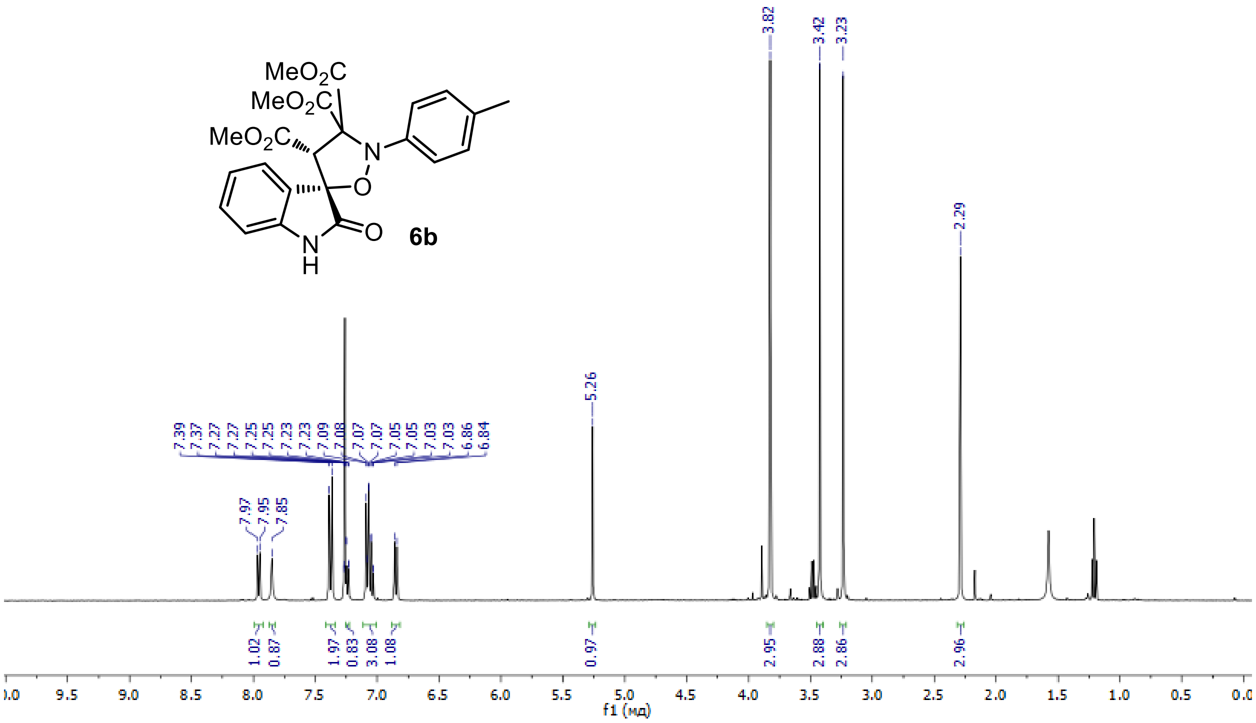

^{13}C NMR (100 MHz, $CDCl_3$) spectrum of compound **6b**

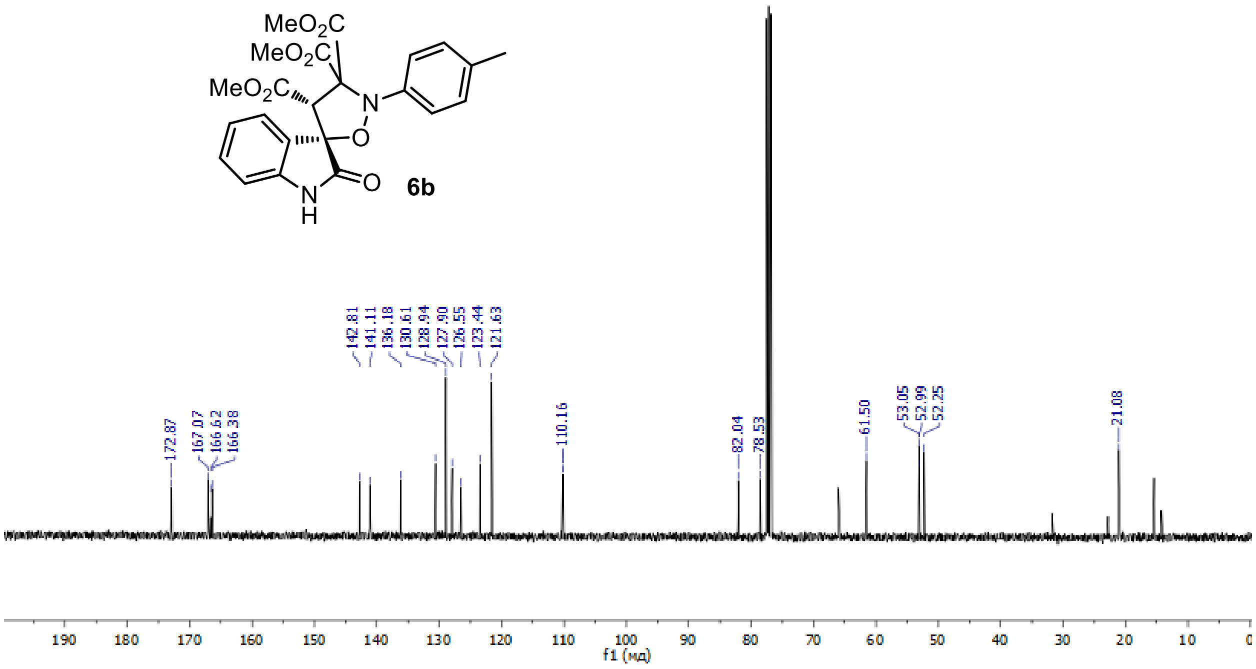

^{1}H NMR (400 MHz, $CDCl_3$) spectrum of compound **6c**

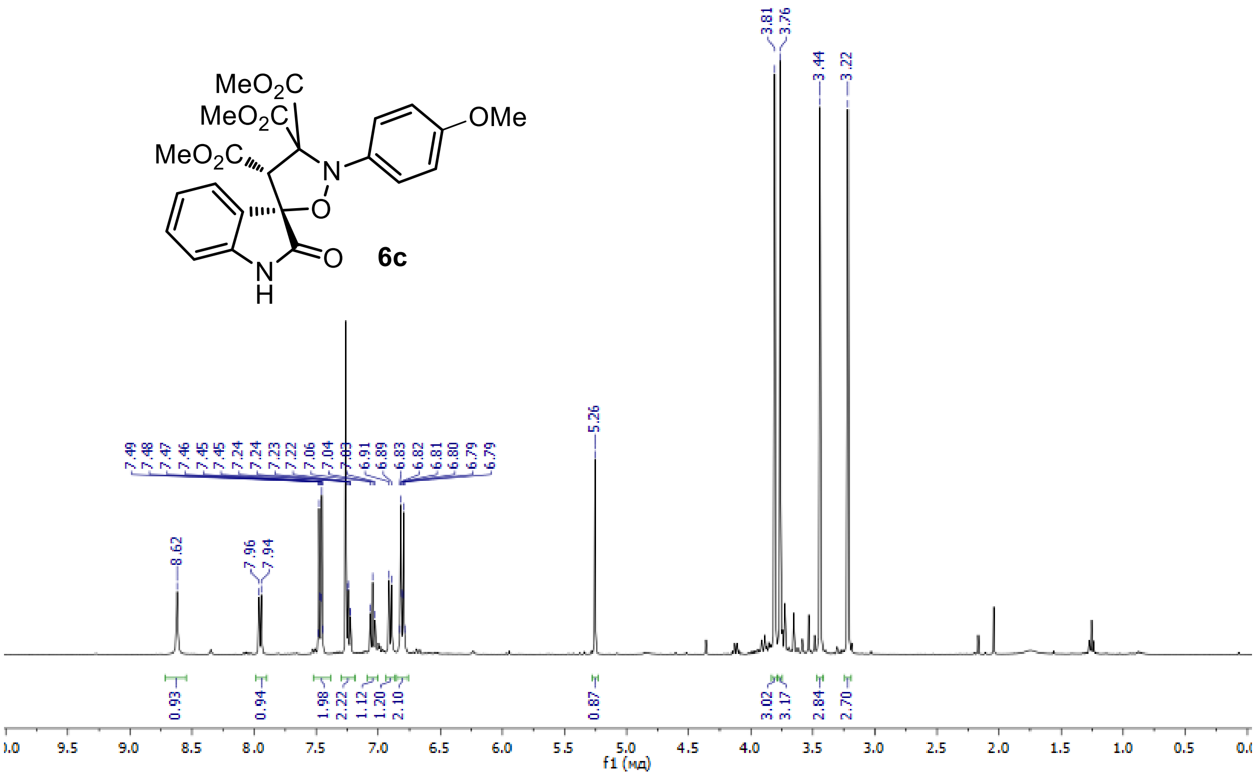

^{13}C NMR (100 MHz, $CDCl_3$) spectrum of compound **6c**

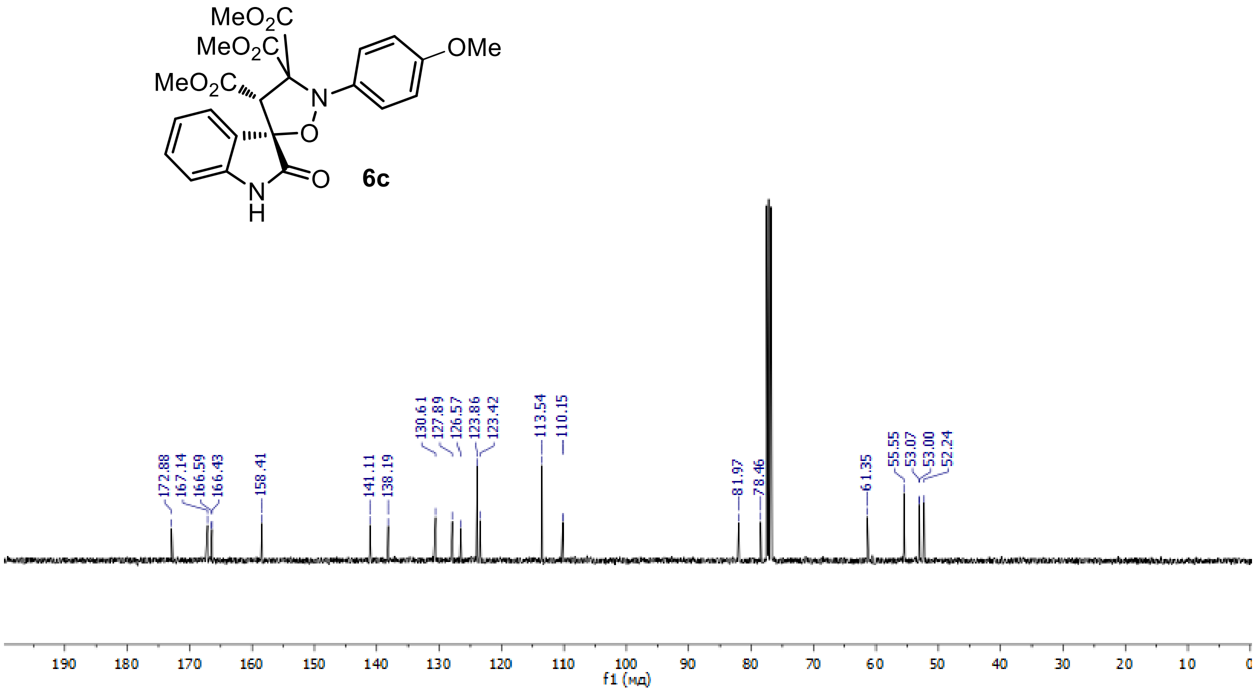

^{1}H NMR (400 MHz, $CDCl_3$) spectrum of compound **6d**

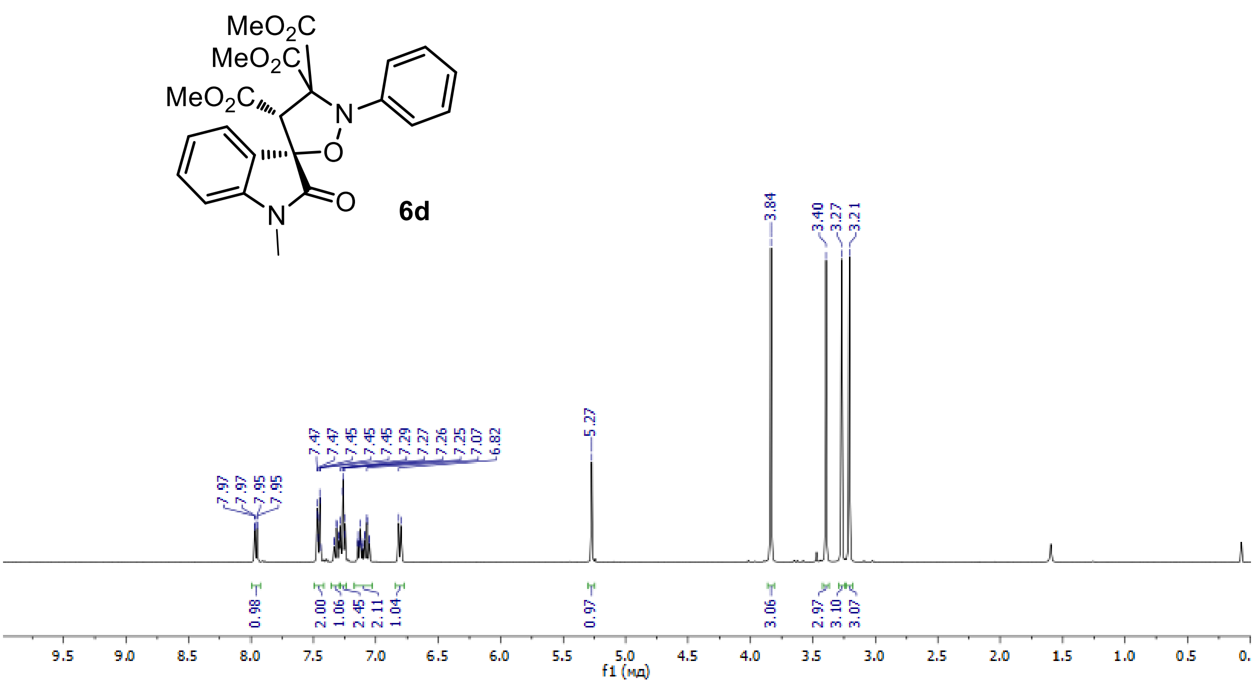

^{13}C NMR (100 MHz, $CDCl_3$) spectrum of compound **6d**

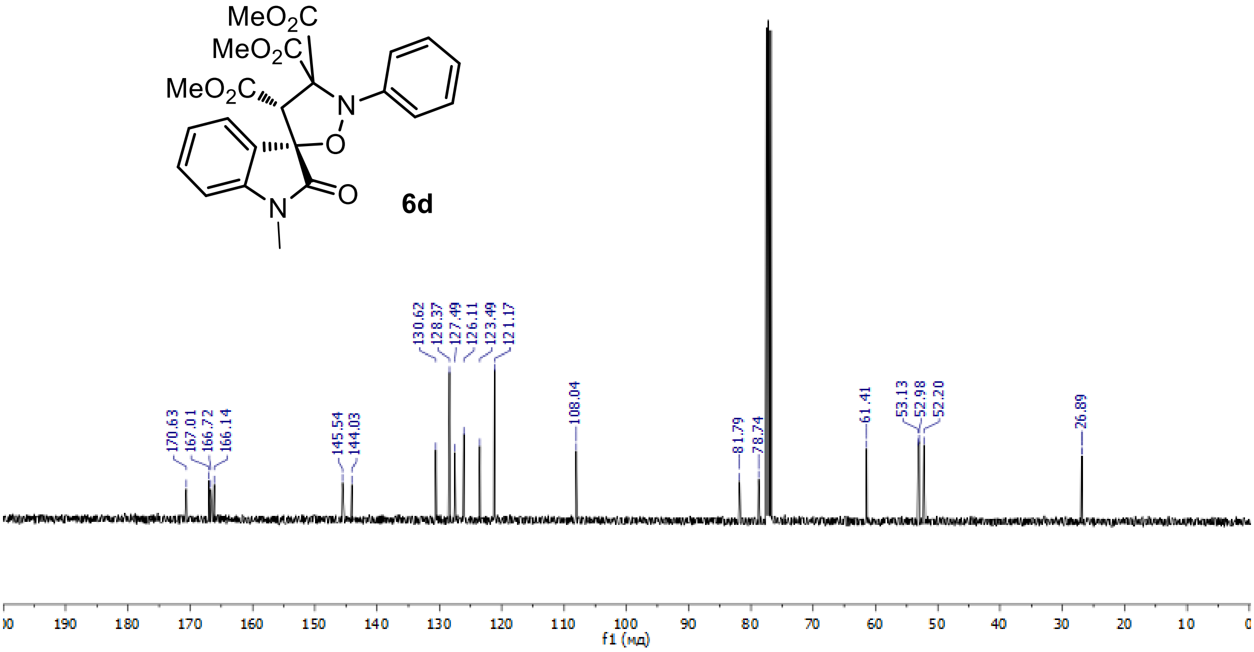

^{1}H NMR (400 MHz, $CDCl_3$) spectrum of compound **6e**

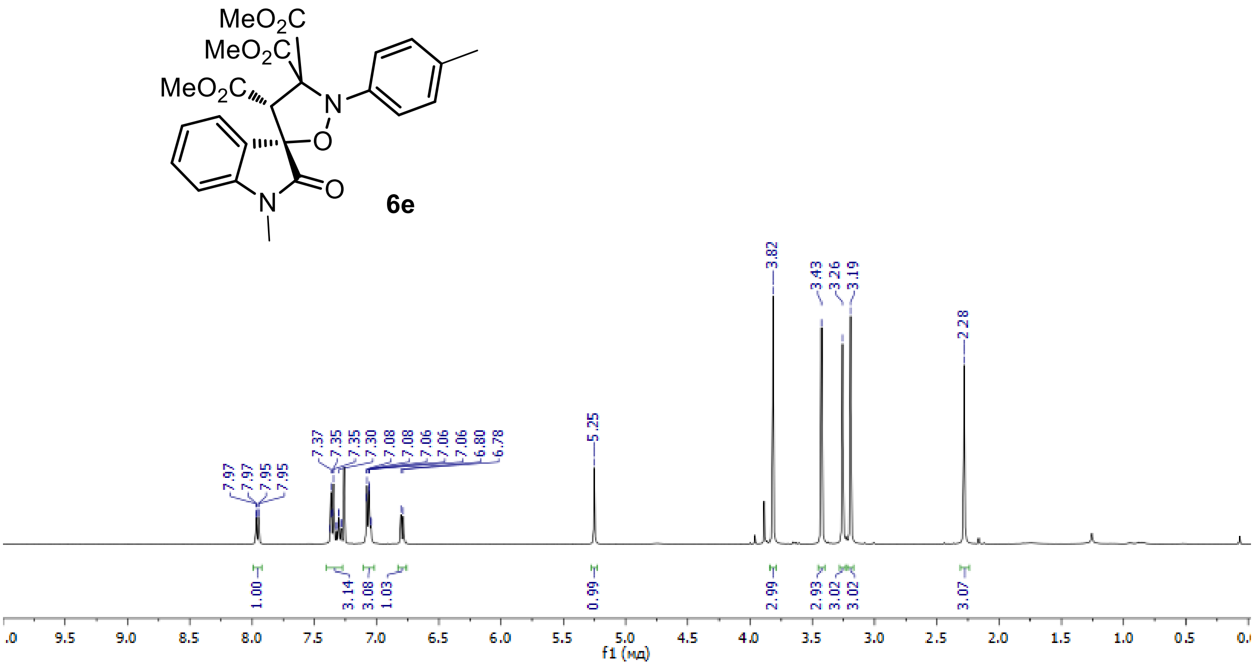

^{13}C NMR (100 MHz, $CDCl_3$) spectrum of compound **6e**

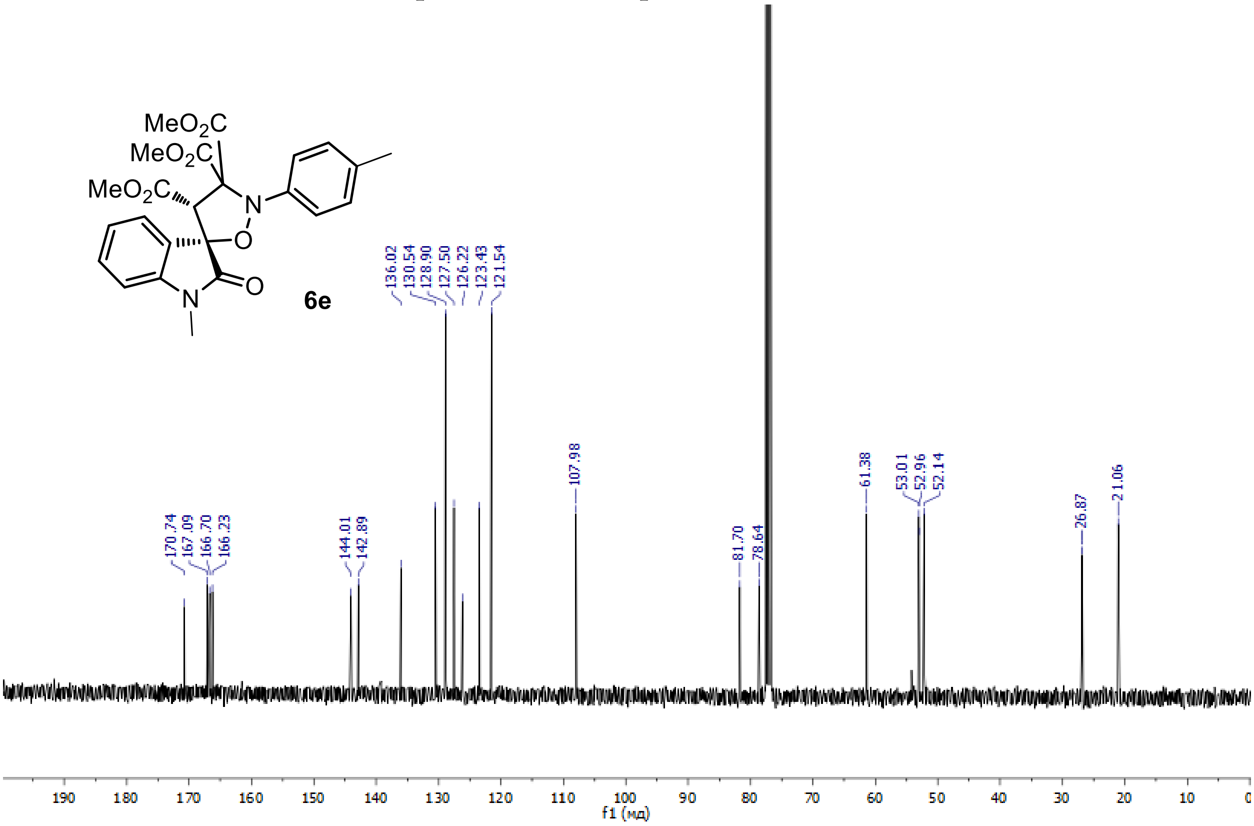

^{1}H NMR (400 MHz, $CDCl_3$) spectrum of compound **6f**

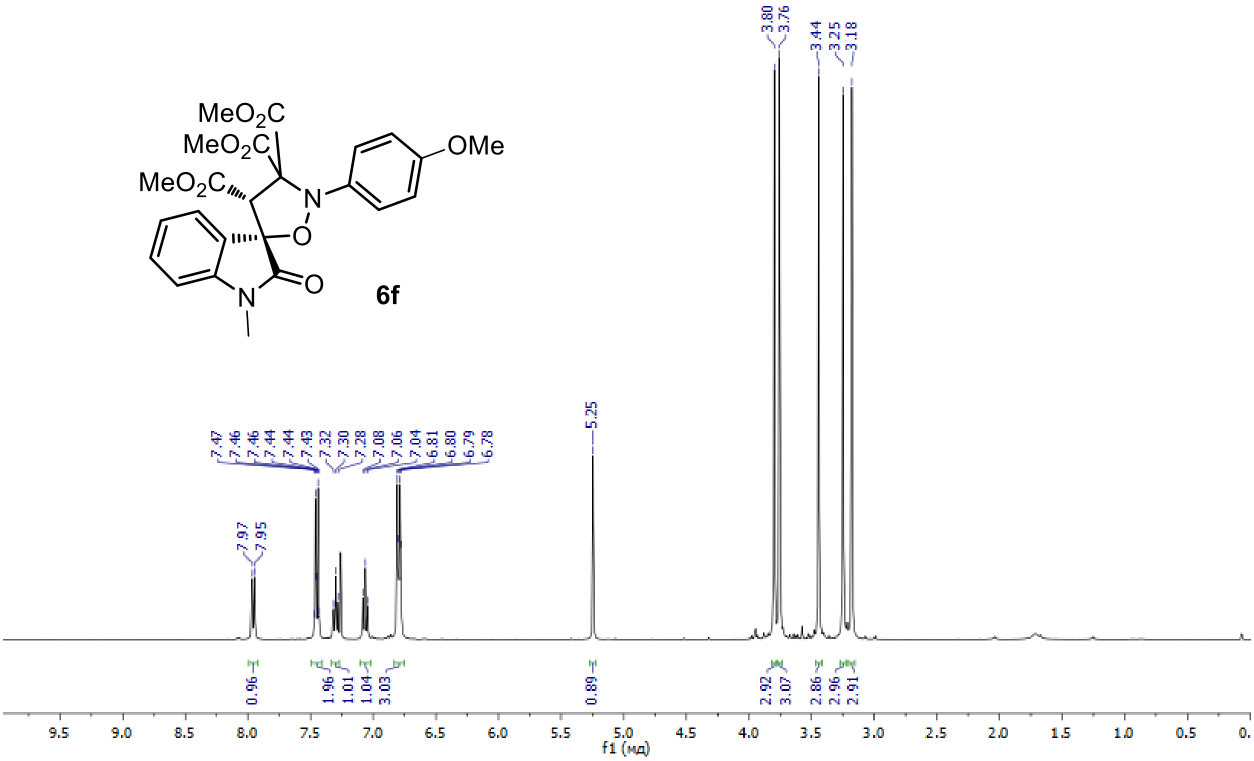

^{13}C NMR (100 MHz, $CDCl_3$) spectrum of compound **6f**

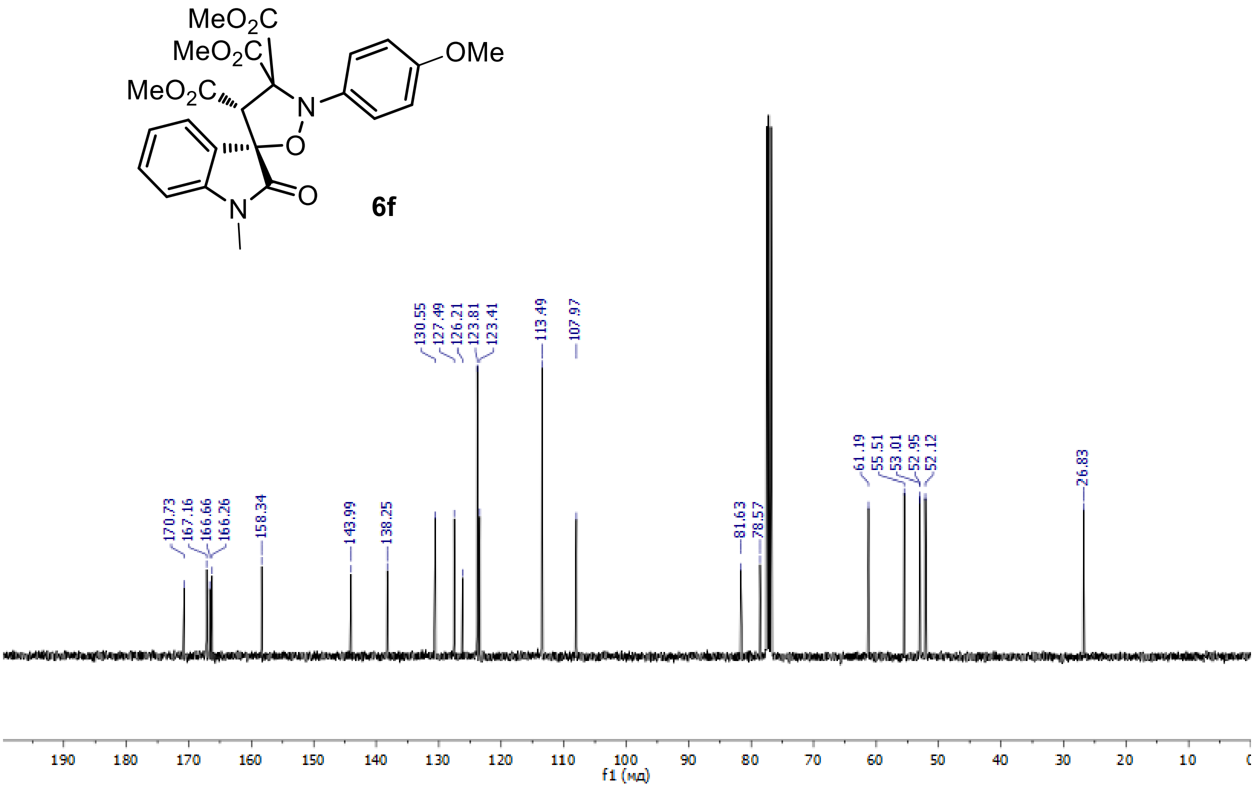

^{1}H NMR (400 MHz, C_6D_6) spectrum of compound **7a**

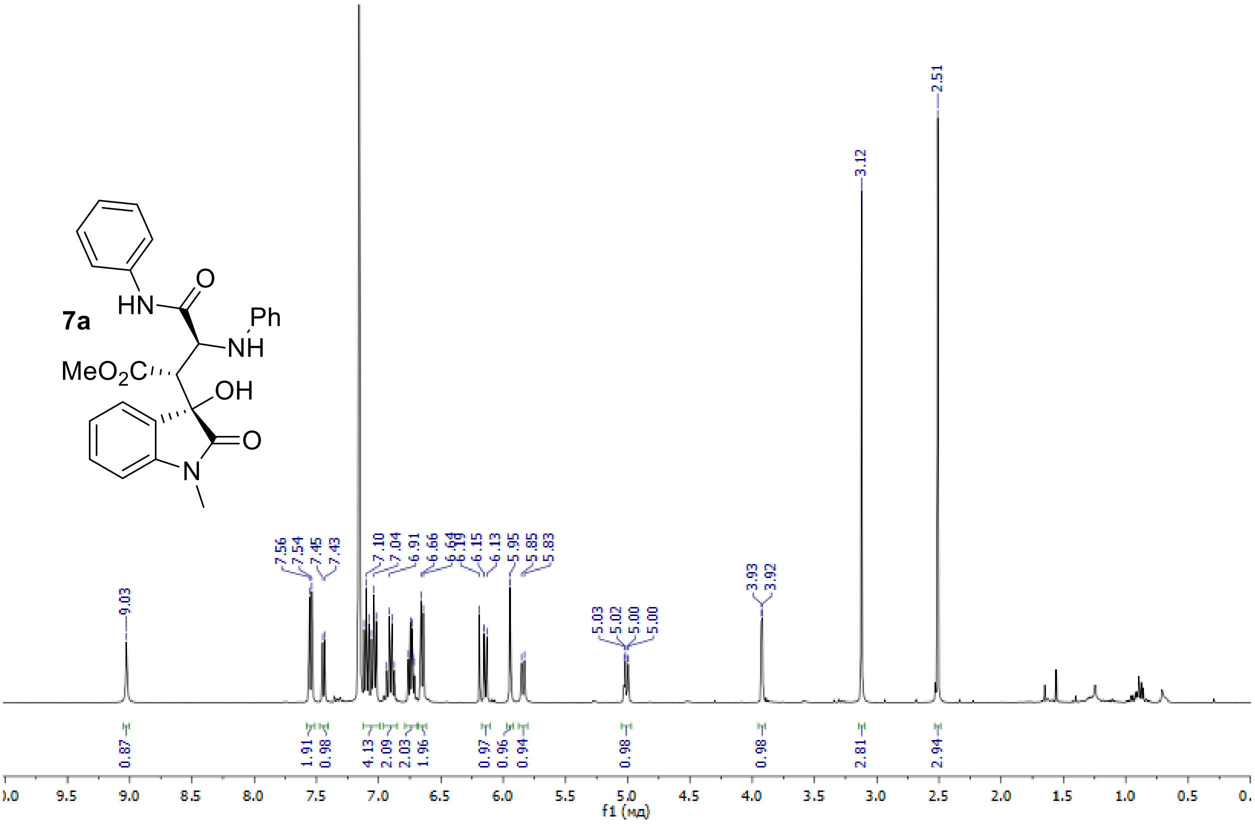

^{13}C NMR (100 MHz, C_6D_6) spectrum of compound **7a**

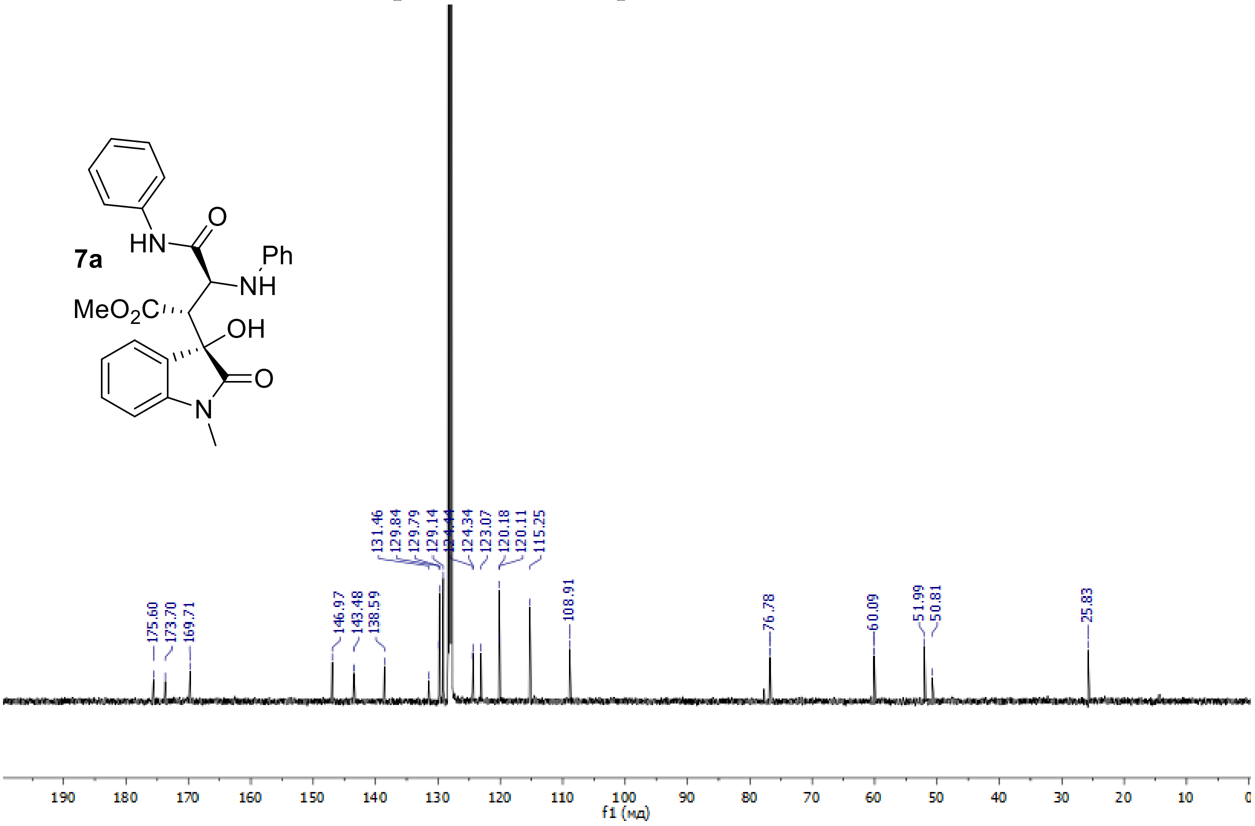

^{1}H NMR (400 MHz, C_6D_6) spectrum of compound **7'a**

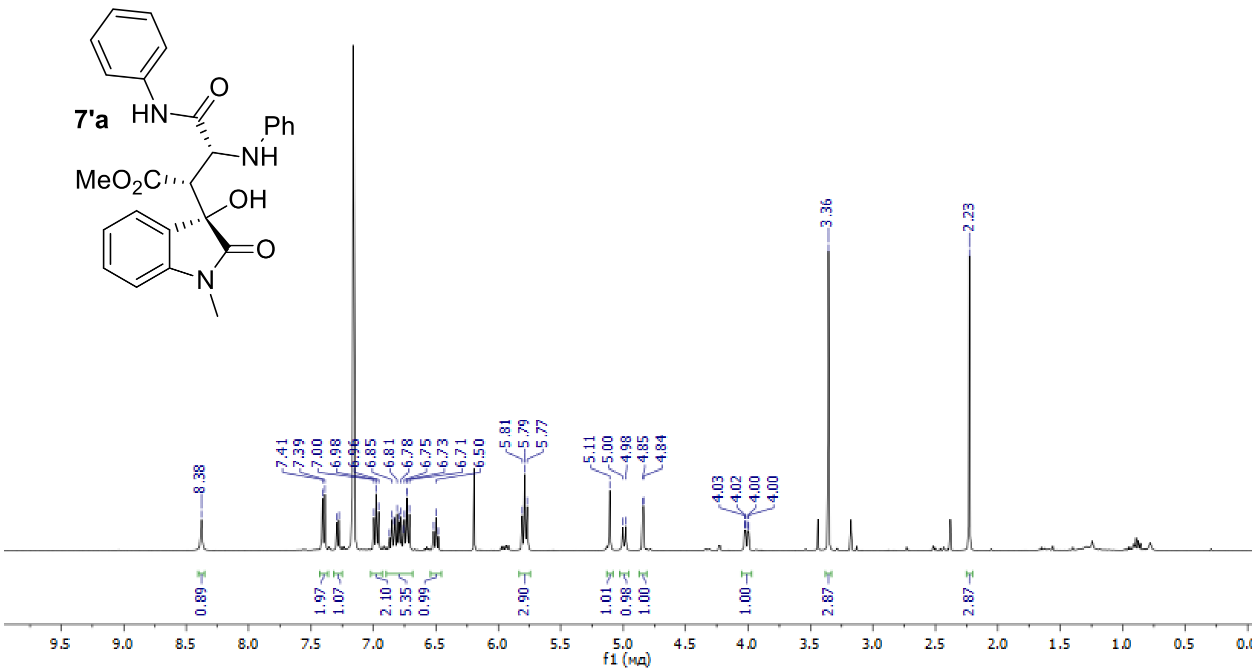

^{13}C NMR (100 MHz, C_6D_6) spectrum of compound **7'a**

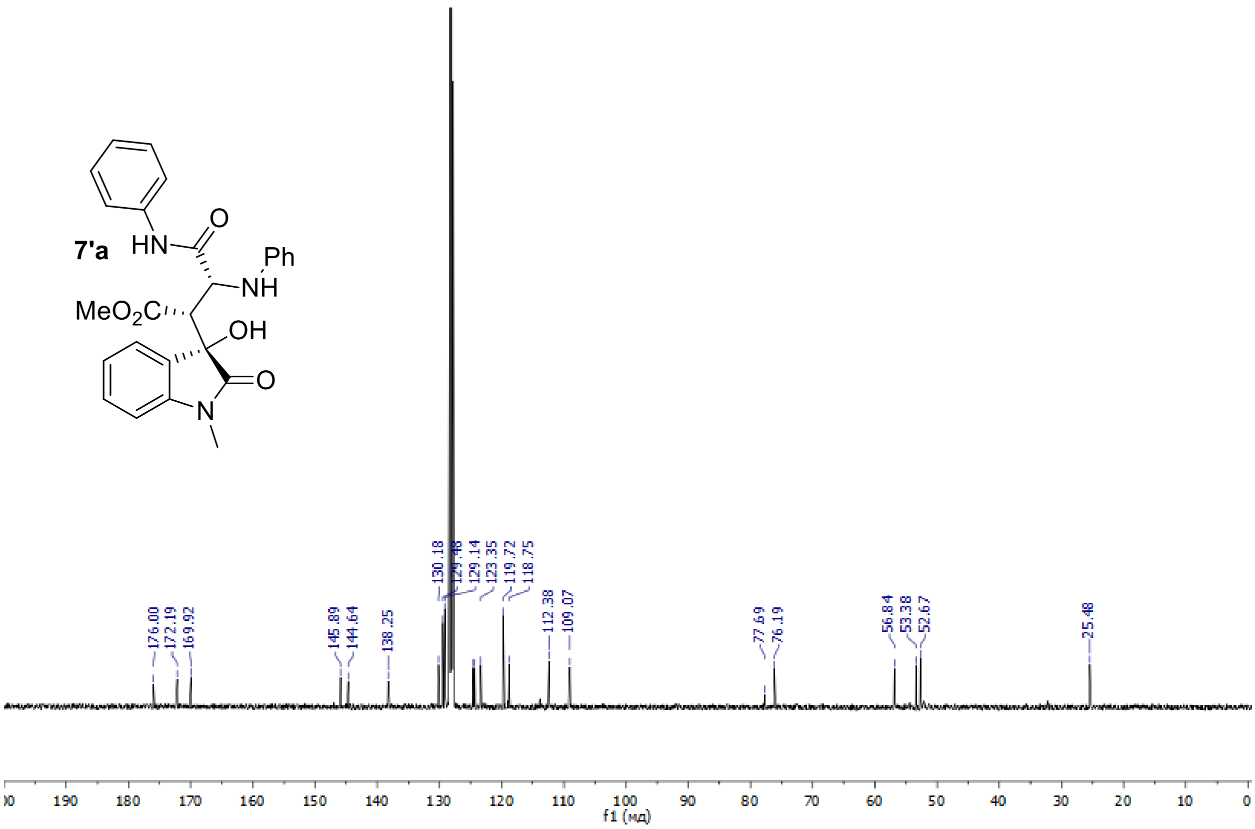

^{1}H NMR (400 MHz, C_6D_6) spectrum of compound **7b**

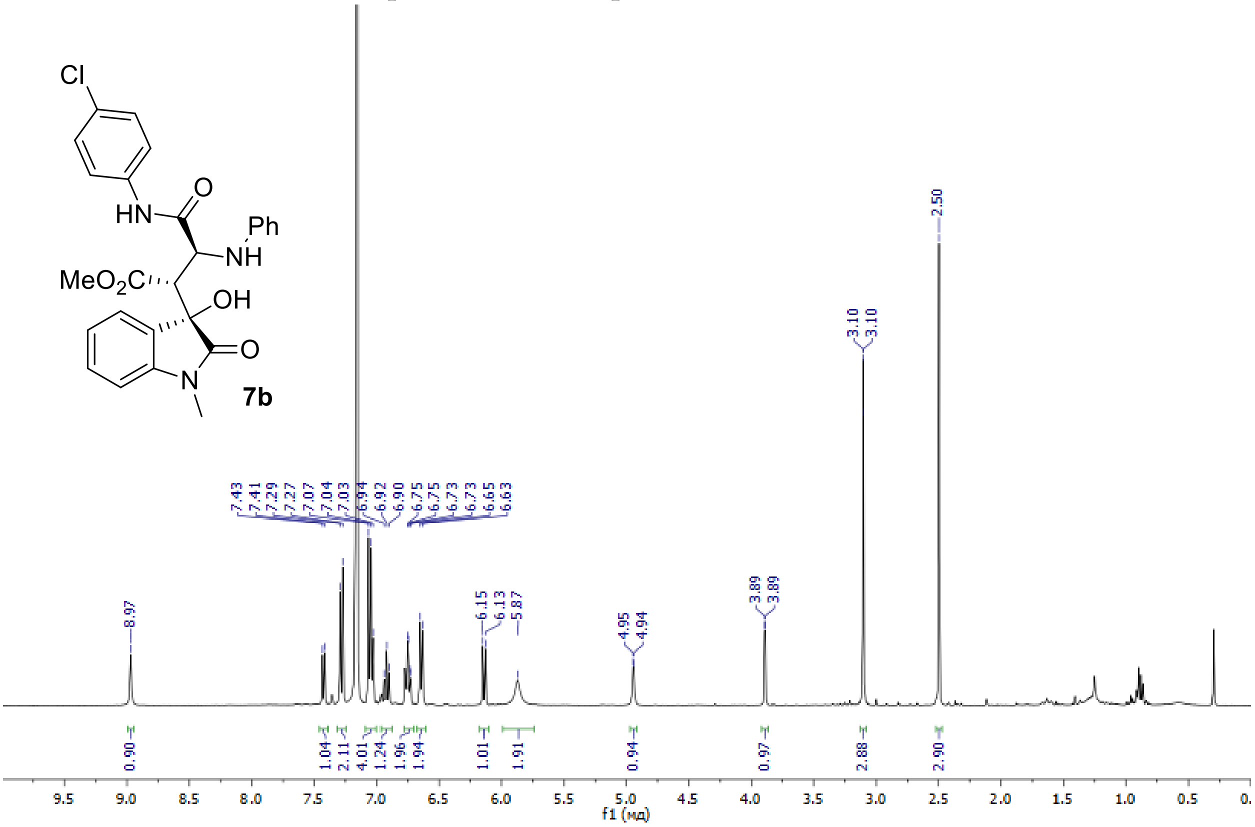

^{13}C NMR (100 MHz, C_6D_6) spectrum of compound **7b**

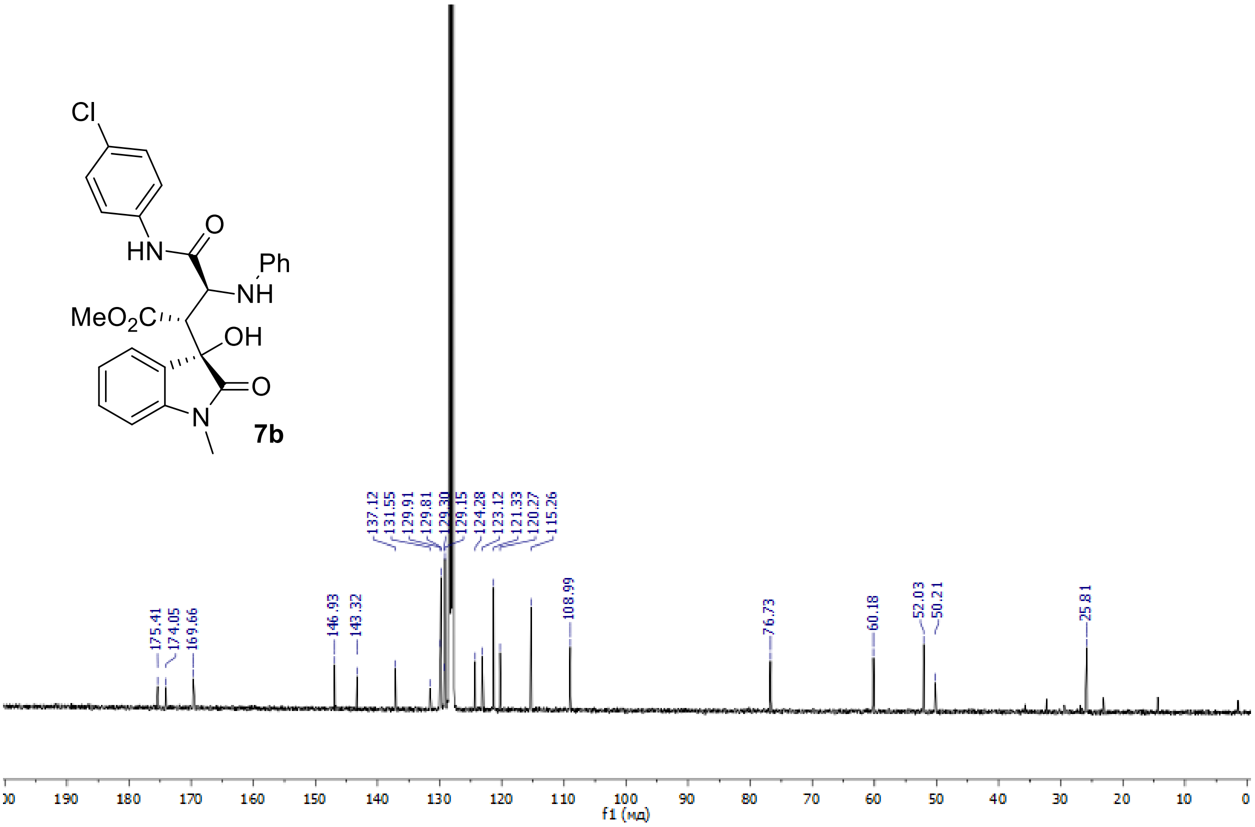

^{1}H NMR (400 MHz, C_6D_6) spectrum of compound **8**

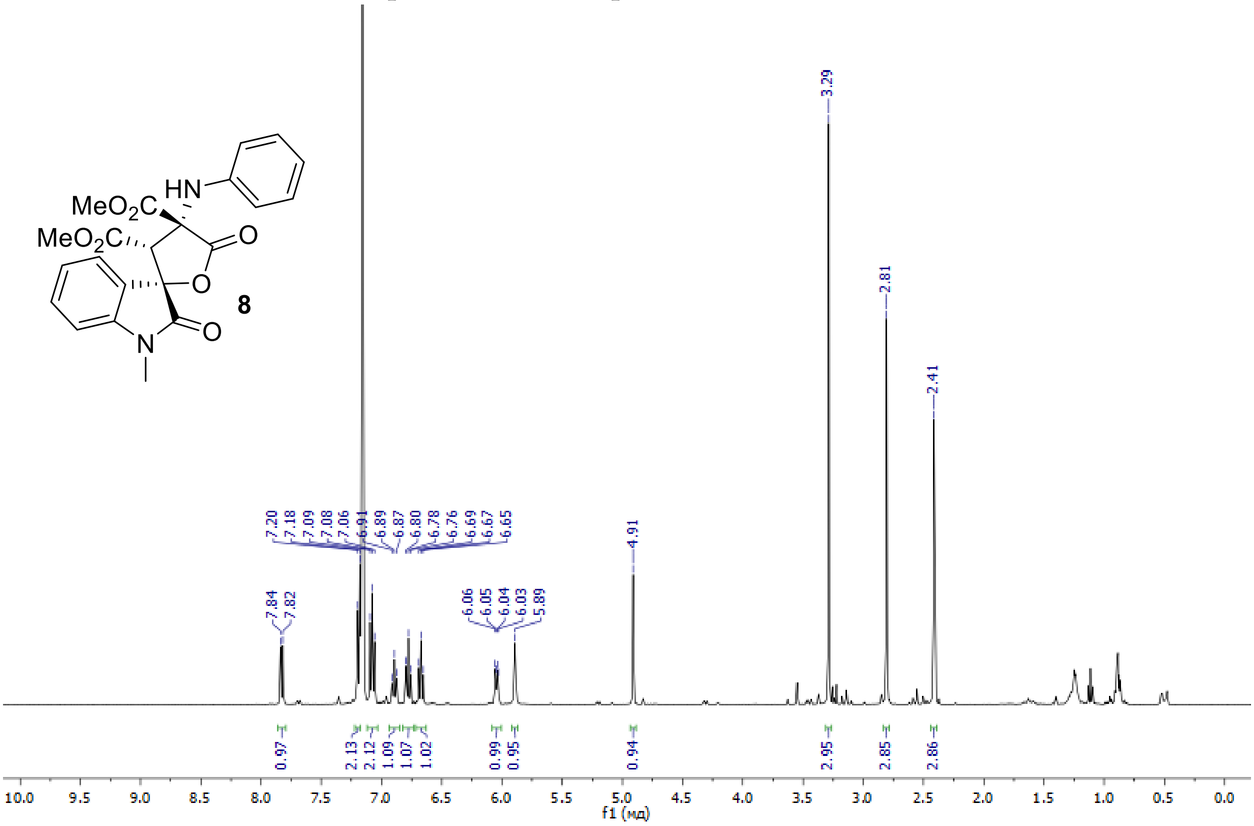

^{13}C NMR (100 MHz, C_6D_6) spectrum of compound **8**

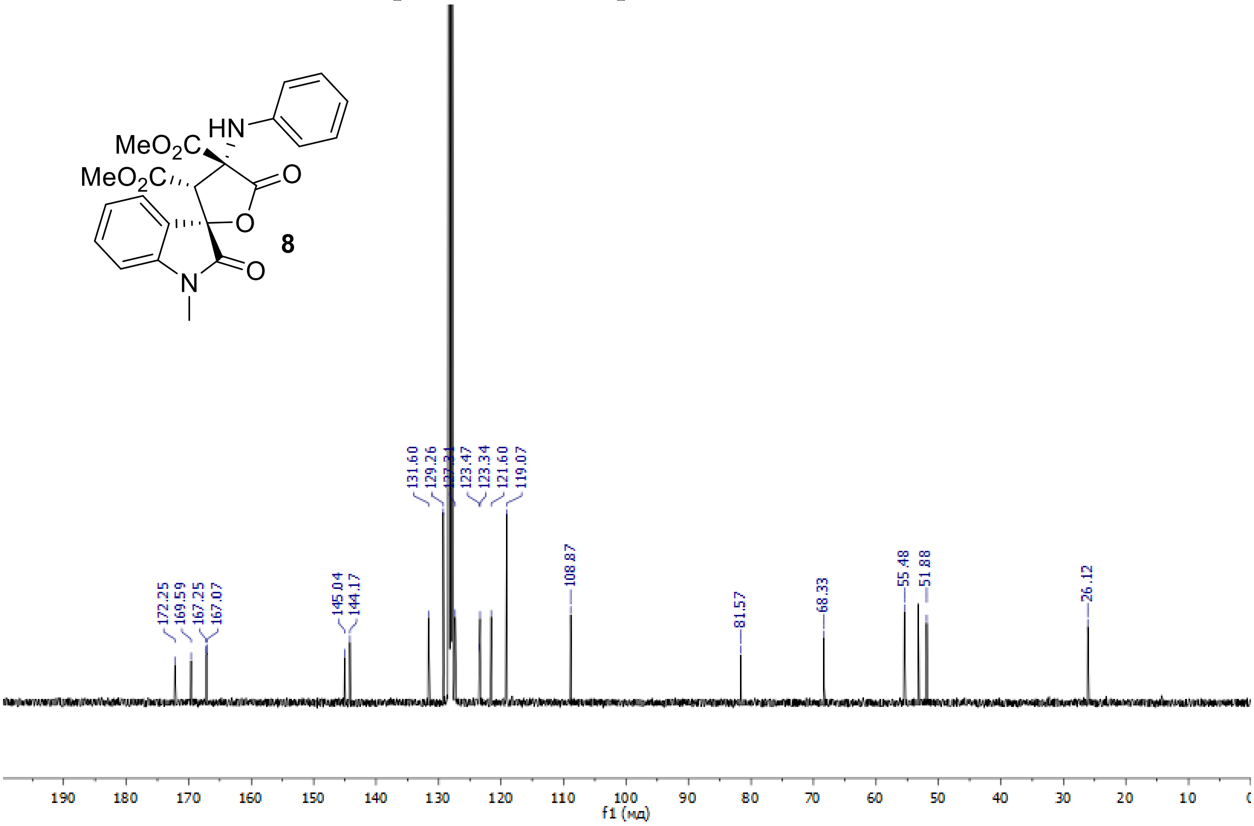

Figure S1. ^{1}H-^{1}H NOESY of **3h**

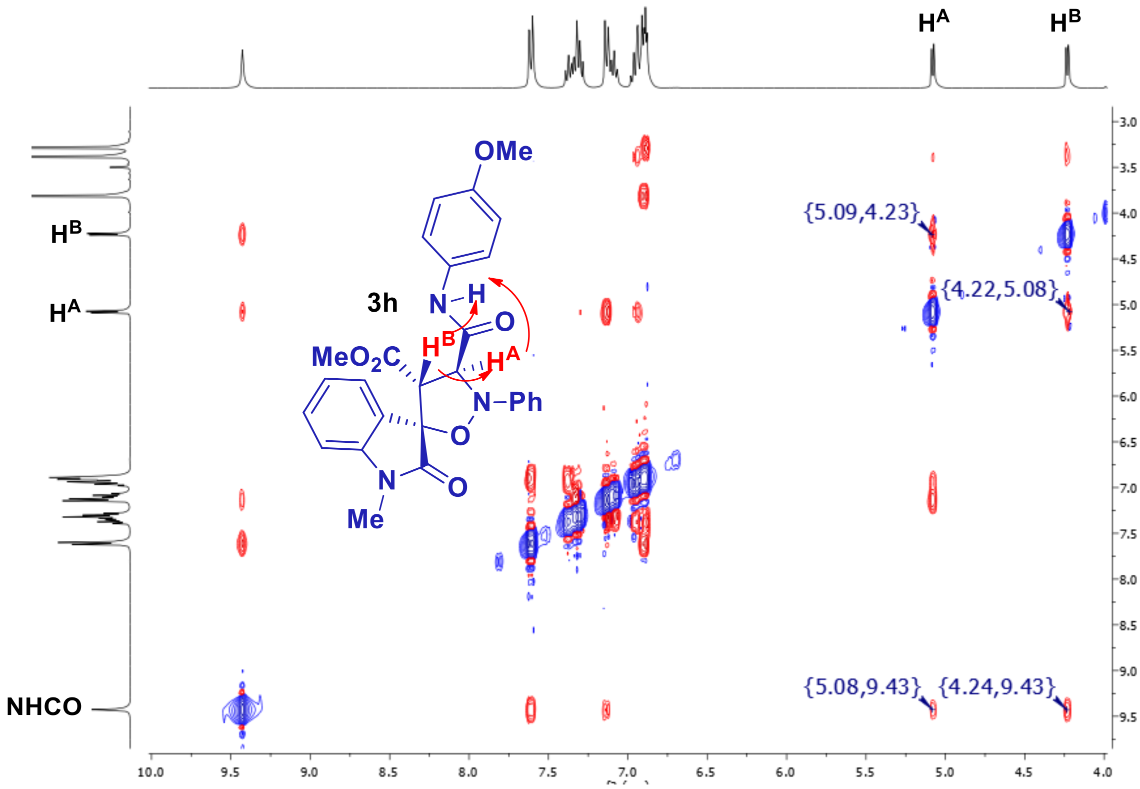

Figure S2. ^{1}H-^{1}H NOESY of **3′h**

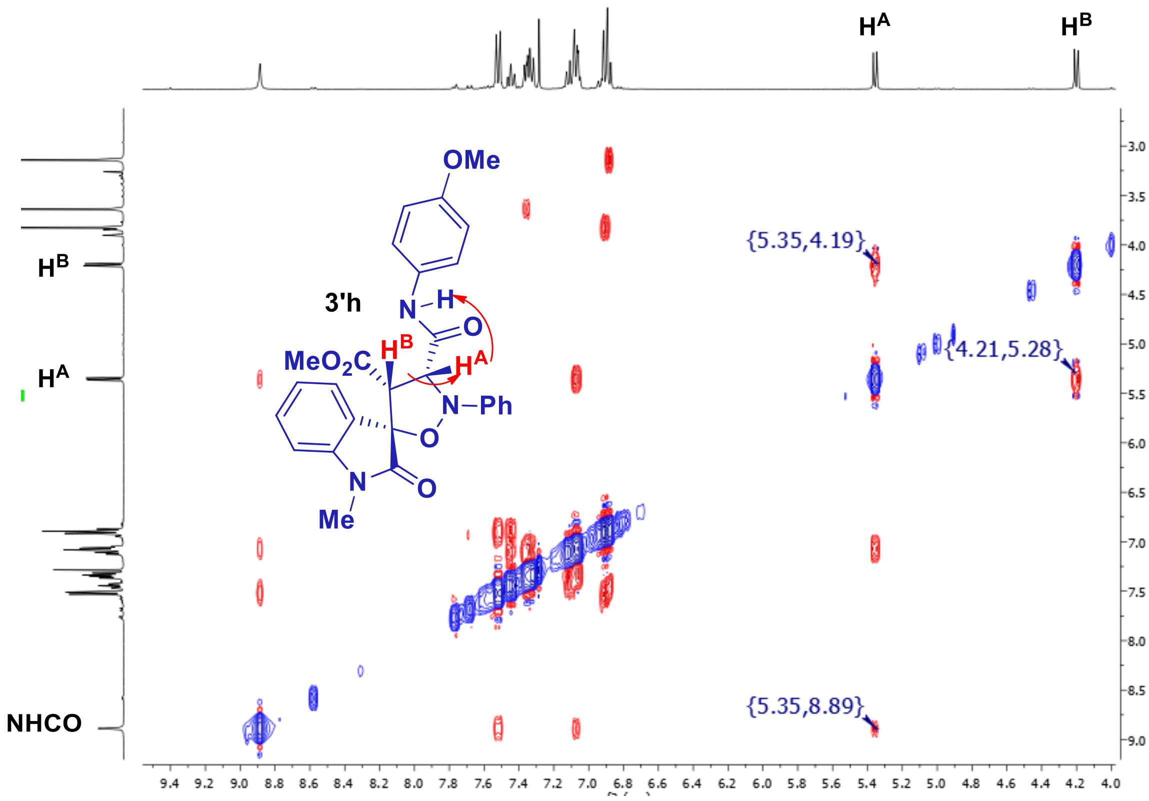

Figure S3. ^{1}H-^{1}H NOESY of **7a**

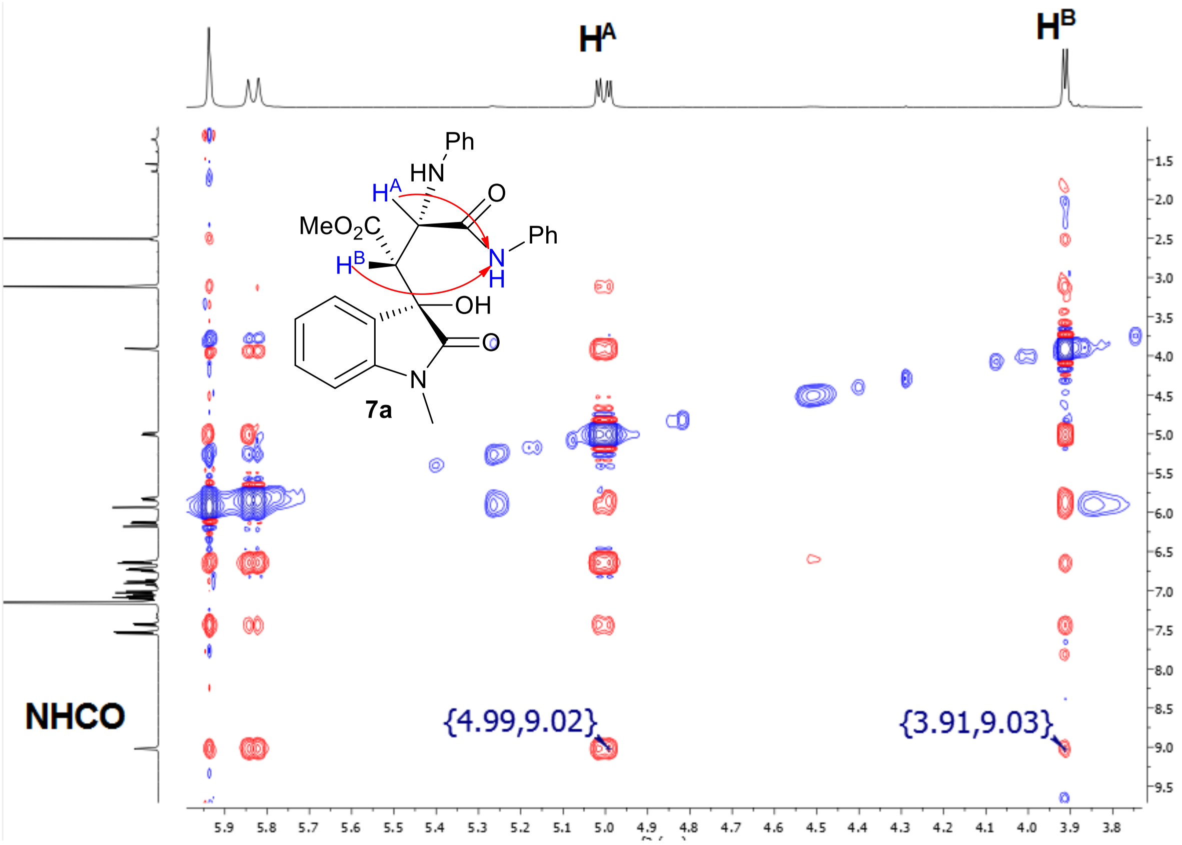

Figure S4. ^{1}H-^{1}H NOESY of **7'a**

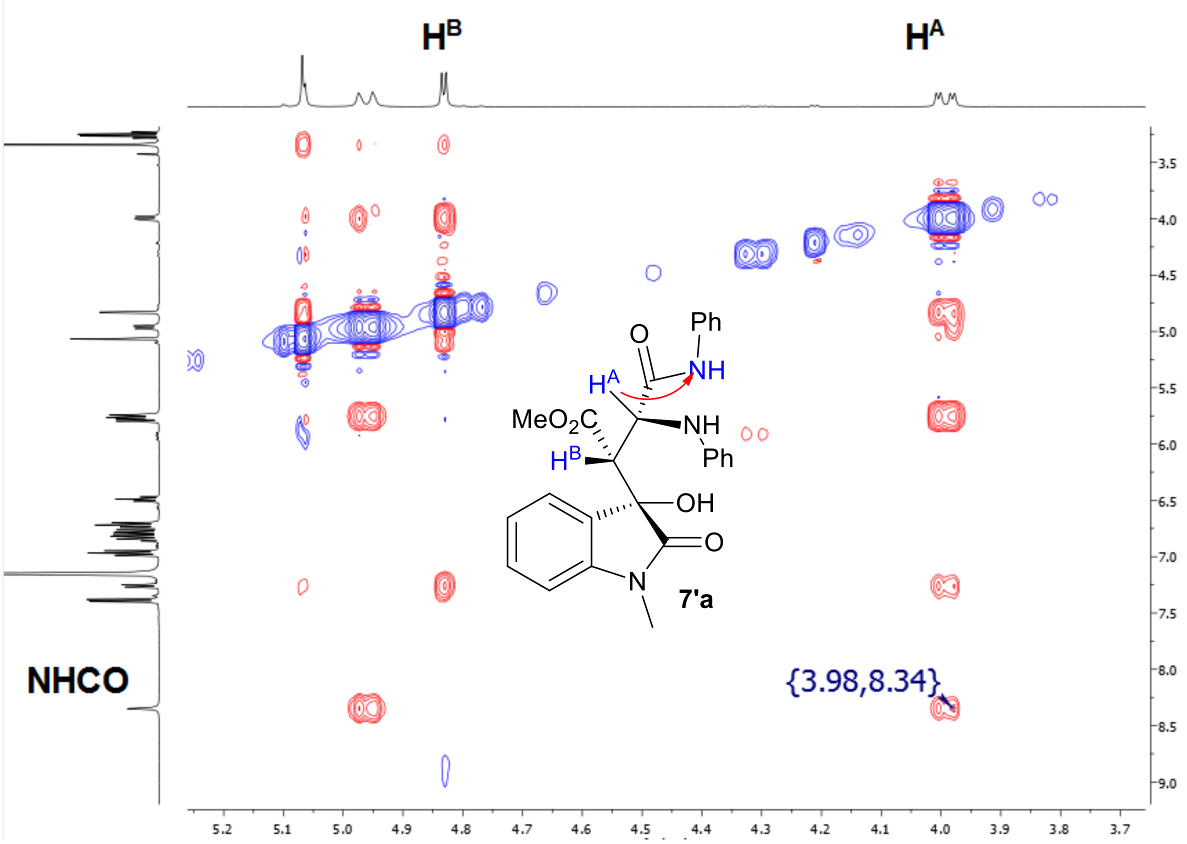

Figure S5. ^{1}H-^{1}H NOESY of **8**

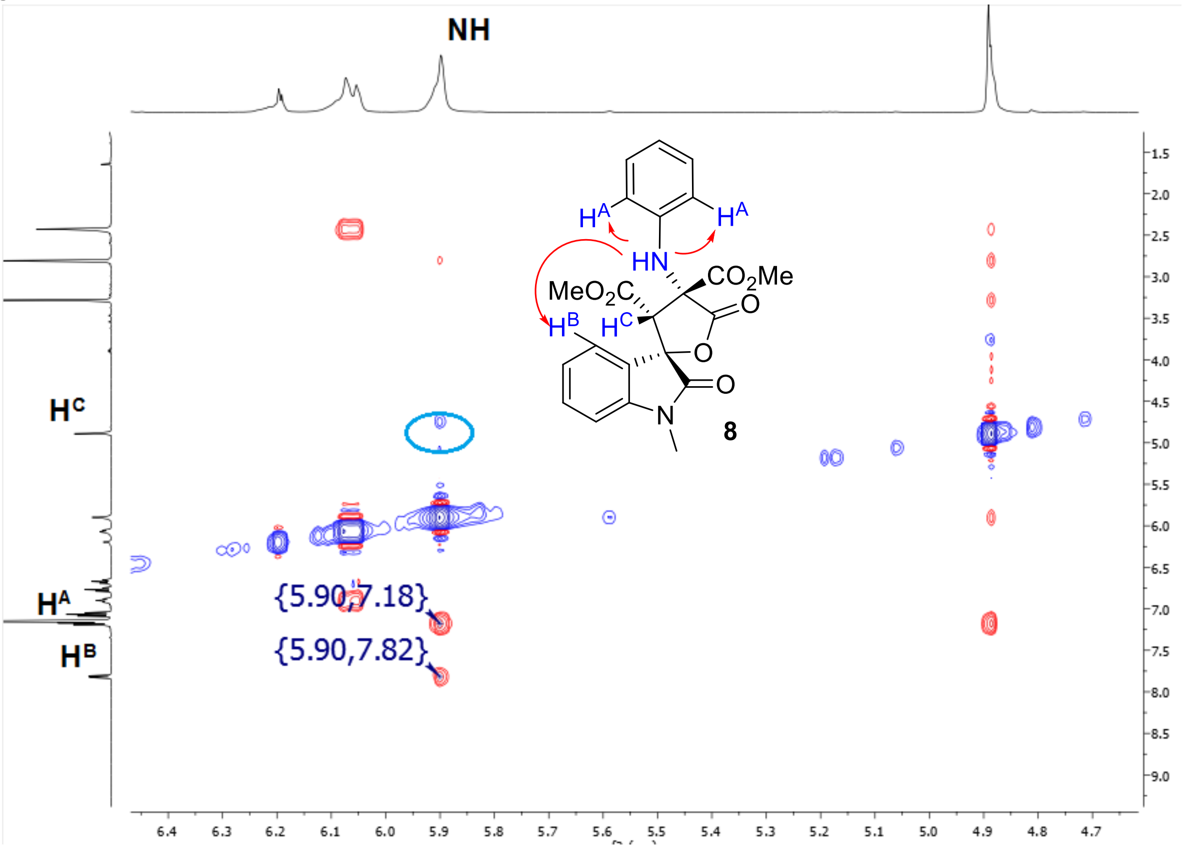

Figure S6. ^{1}H-^{1}H NOESY of the mixture of **3′c** and **4c**

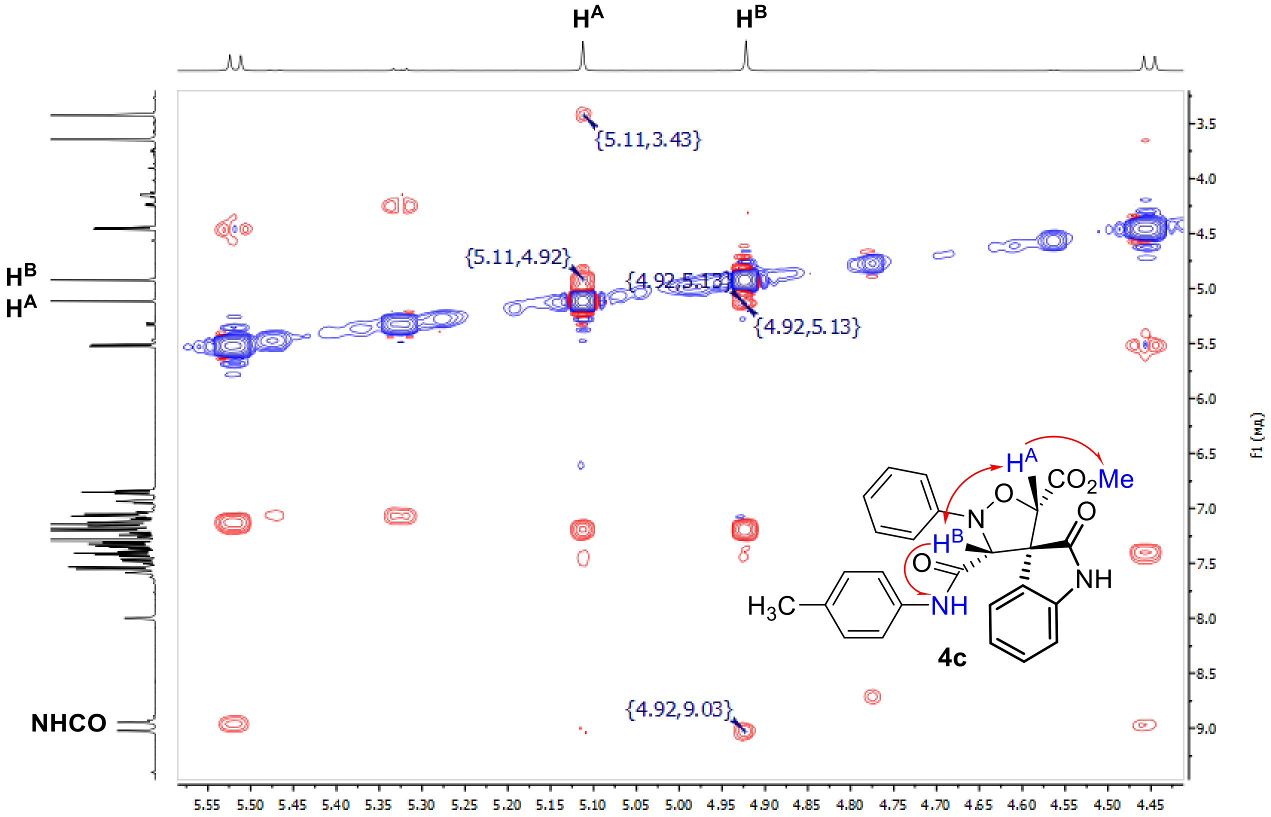

Supplement: Supplementary file 1 [file ijms-23-12639-s001.zip › ijms-1976480-supplementary.pdf]
